# Music mindfulness acutely modulates autonomic activity and improves psychological state in anxiety and depression

**DOI:** 10.3389/fnins.2025.1554156

**Published:** 2025-04-08

**Authors:** Christine Ramirez, Gertrude Asumpaame Alayine, Cyril Selase Kwaku Akafia, Kamsiyonna Adichie, Dash Watts, Yizza Galdamez, Lisa Harding, AZA Stephen Allsop

**Affiliations:** ^1^AZA Lab, Department of Psychiatry, Yale University, New Haven, CT, United States; ^2^Department of Psychiatry and Behavioral Sciences, Center for Collective Healing, Howard University, Washington, DC, United States; ^3^Depression MD, Mood Disorder Institute, Milton, CT, United States

**Keywords:** music, mindfulness, heart rate variability, electroencephalography, depression, anxiety, stress

## Abstract

**Introduction:**

Anxiety and depression reduce autonomic system activity, as measured by Heart Rate Variability (HRV), and exacerbate cardiac morbidity. Both music and mindfulness have been shown to increase HRV, and clinical approaches incorporating these interventions show promise as effective treatments for symptoms of anxiety and depression. Music mindfulness, which combines music listening with mindfulness activities, may provide unique and synergistic therapeutic benefits for stress management. However, to date, no studies have evaluated the physiological mechanisms underlying a community-based music mindfulness paradigm.

**Methods:**

We used wearable technology to record electrocardiography and electroencephalography signals from participants with moderate symptoms of anxiety and depression during a community-based music mindfulness paradigm. We also assessed the impact of our music mindfulness session on participant’s psychological state.

**Results and discussion:**

We found that music mindfulness sessions acutely enhanced multiple measures of HRV and altered EEG power spectral density across various frequency bands in frontotemporal electrodes. Both live and virtual music mindfulness sessions also acutely reduced stress and altered participants’ state of consciousness; however, only live sessions fostered social connection. Additionally, the physiological and psychological effects of music mindfulness varied based on participants’ self-reported sex. Overall, our findings demonstrate that music mindfulness effectively engages autonomic and frontotemporal neural mechanisms, which may contribute to the treatment of anxiety and depression symptoms.

## Introduction

Music-based interventions (MBIs) are promising community-based therapies for stress-related conditions such as anxiety and depression (Fancourt et al., 2016; Bradt et al., 2013; Nilsson, 2008; Aalbers et al., 2017; Maratos et al., 2008; Chu et al., 2014; Chen et al., 2022; Cheever et al., 2018; Collins and Fleming, 2017; Smoller, 2016). MBIs also modulate neural networks that are important for emotional regulation and cognitive function (Blood and Zatorre, 2001; Daly et al., 2014; Gosselin et al., 2007; Salimpoor et al., 2011; Koelsch et al., 2021; Cheung et al., 2019; Faber et al., 2024). While music alone can effectively alleviate symptoms of stress, it also serves as a preferred and effective complement that enhances meditation and mindfulness practices (Dvorak and Hernandez-Ruiz, 2021; Hernandez-Ruiz and Dvorak, 2021). Various mindfulness practices have been tailored for therapeutic use and have demonstrated positive effects on anxiety, depression, and chronic pain (Hofmann and Gómez, 2017; Goldin and Gross, 2010; Eckhardt and Dinsmore, 2012; Hilton et al., 2017). Brief daily meditation practices improve mood and cognitive function in new meditators (Basso et al., 2019; Moore et al., 2012), while online mindfulness interventions have also exhibited positive outcomes for stress, mood, and cognition (Finnerty et al., 2023; Kirk and Axelsen, 2020).

Music mindfulness combines elements of music listening with mindfulness practices and may provide synergistic therapeutic benefits for stress-related conditions (Eckhardt and Dinsmore, 2012; Soo et al., 2016; Lesiuk, 2015; Vidyarthi et al., 2012) (Table 1). However, advancing music mindfulness approaches requires a better understanding of its mechanisms of action on physiological and psychological states (Chen et al., 2024). Music modulates both the parasympathetic and sympathetic branches of the autonomic nervous system (McCrary and Altenmüller, 2021; McPherson et al., 2019; Xiao et al., 2023; Kume et al., 2017; Nakashima et al., 2013). Heart rate variability (HRV) reflects fluctuations in heart rate and serves as an index for autonomic nervous system activity (Tang et al., 2009; Nijjar et al., 2014; Peng et al., 2004). Importantly, reduced HRV is commonly found in various psychiatric conditions, such as anxiety, depression, and PTSD (Kume et al., 2017; Berntson and Cacioppo, 2004; Ramesh et al., 2023; Moon et al., 2013). Moreover, reduced HRV is a significant mechanism contributing to increased cardiac morbidity in psychiatric conditions (Chalmers et al., 2014). While music and mindfulness can independently enhance HRV, this is not always consistent, and it remains unclear whether music combined with meditation can yield synergistic effects on autonomic physiology (Kirk and Axelsen, 2020; Ellis and Thayer, 2010; Koelsch and Jäncke, 2015; Christodoulou et al., 2020; Krygier et al., 2013; Mitcheff et al., 2021; Kirk et al., 2022; Lee and Bhattacharya, 2013).

**Table 1:** Summary table of music mindfulness studies.

Title	Author-year	Subjects	Major result	Limitations
Comparison of music stimuli to support mindfulness meditation	Dvorak and Hernandez-Ruiz (2021)	*n* = 57	Participants ranked the Melody and Harmony stimuli as the most useful and most preferred.	No HR, no EEG, no clinical application, and no live music
Imaging-guided core-needle breast biopsy: impact of meditation and music interventions on patient anxiety, pain, and fatigue	Soo et al. (2016)	*n* = 121	Meditation significantly lowered pain during imaging-guided breast biopsy; meditation and music reduced patient anxiety and fatigue compared to standard care.	No HR, no EEG, and did not combine music and mindfulness
The effect of mindfulness-based music therapy on attention and mood in women receiving adjuvant chemotherapy for breast cancer: a pilot study	Lesiuk (2015)	*n* = 15	Attention improved significantly over time. The mood state of fatigue decreased significantly more than the other mood states.	No HR and no EEG
Effect of music on a mindfulness experience: an online study	Hernandez-Ruiz et al. (2021)	*n* = 54	Results indicated no significant interactions with the AIMS and PSS-10 scores and no significant differences in music stimuli in the mindfulness meditation.	No HR, no EEG, and no clinical application
Effects of mindfulness meditation on musical aesthetic emotion processing	Liu et al. (2021b)	*n* = 67	Maintaining a state of mindfulness while listening to music enhanced body awareness and led to experiencing a faster passage of musical time.	No HR, no EEG, no clinical application, and no live musician
Exploring the feasibility of a mindfulness-music therapy intervention to improve anxiety and stress in adolescents and young adults with cancer	Knoerl et al. (2022)	*n* = 37	Participation in the mindfulness-based music therapy sessions resulted in significant pre-to-post test improvements in perceived stress.	No HR and no EEG
Mindfulness, attention, and flow during music listening: an empirical investigation	Diaz (2013)	*n* = 132	Heightened attention during music listening compared to baseline.	No HR, no EEG, no clinical application, and no live musician
Participants’ experiences of music, mindful music, and audiobook listening interventions for people recovering from stroke	Baylan et al. (2018)	*n* = 56	Mindful music listening was most strongly associated with relaxation and concentration, improved attentional control, and emotion regulation.	No HR, no EEG, and no live musician
Effects of mindfulness-based stress reduction combined with music therapy on pain, anxiety, and sleep quality in patients with osteosarcoma	H. Liu et al. (2019)	*n* = 121	8 weeks of the combined Mindfulness Based Stress Reduction/Music Therapy intervention reduced pain and anxiety scores and improved the quality of sleep.	No HR and no EEG
Regulation of mindfulness-based music listening on negative emotions related to COVID-19: an ERP study	Liu et al. (2021a)	*n* = 85	ERP results showed negative mood states elicited greater N2, N3, and LPC amplitudes and smaller P3 amplitudes.	No HR, no spectral data, and no live musician
An eight-week Zen meditation and music programme for mindfulness and happiness: qualitative content analysis	Hwang et al. (2023)	*n* = 9	Significant improvements in subjective happiness and mindfulness. Additionally, it enhanced participants’ capacity to handle emotional challenges and effectively manage stress.	No HR, no EEG, and no quantitative analysis
Emotion regulation through music and mindfulness are associated with positive solitude differently in the second half of life	Bachman et al. (2022)	*n* = 123	In the second half of life, the Positive Solitude skill is associated with the skills of emotion regulation through music listening and mindfulness.	No HR, no EEG, and only self-report,
Sitting Meditation (Mindfulness) and Music Meditation Effects on Overall Anxiety and Test Anxiety in a College Student Population	Lothes et al. (2022)	n = 31	Participants in the sitting meditation condition showed significant within-group reductions in test anxiety, overall anxiety, and mindfulness from start to finish. The music meditation group showed no changes in test anxiety. But had decreased anxiety scores and overall increased mindfulness.	No HR and no EEG
Clinical effectiveness of mindfulness-based music therapy on improving emotional regulation in blind older women: a randomized controlled trial	Chan et al. (2023)	*n* = 92	The Music-Based Music Therapy group improved emotional awareness and appeared to lower depression scores.	No HR and no EEG
Effects and feasibility of a mindfulness-based Guqin music intervention during pregnancy on postpartum anxiety and depression: a pilot randomized controlled trial	Qi et al. (2023)	*n* = 87	Mindfulness-based Guqin music intervention contributes to a decrease in postpartum anxiety and depression, potentially by enhancing mindful attention and awareness.	No HR and no EEG
A digital music-based mindfulness intervention for black Americans with elevated race-based anxiety: a multiple-baseline pilot study	Jones et al. (2023)	*n* = 5	A digital music-based mindfulness intervention can decrease race-based anxiety in Black Americans.	No HR, no EEG, feasibility study, and small sample size
A virtual music mindfulness tool for individuals of African descent during COVID-19	Igbinobaro et al. (2024)	n = 10	A correlation between increased time engaged in listening to music mindfulness platforms and decreased perceived stress.	No HR, no EEG, small sample size, and pre-print
Mindful music – a pilot study of the effects of mindfulness-based music on staff members of the University of Limerick	Alqatari et al. (2022)	*n* = 63	The three most common feeling states recorded after the intervention were relaxed, hopeful, and positive. Live music and live facilitated sessions (online or in person) were preferred to recorded sessions.	No HR, no EEG
Exploring young adults’ perspectives of participation in a mindfulness-based music therapy intervention before and during the COVID-19 pandemic	Phillips et al. (2023)	*n* = 16	Participants experienced a sense of relaxation in response to intervention participation. Virtual group participants reported that practicing music and mindfulness together was synergistic, and in-person intervention delivery was preferred to virtual delivery.	No HR, no EEG, no quantitative analysis
Effects of monochord music on heart rate variability and self-reports of relaxation in healthy adults	Gäbel et al. (2017)	*n* = 70	Participants in both receptive live music and prerecorded relaxation exercise groups showed psychophysiological changes indicative of greater relaxation over the course of the interventions. However, differences between groups were only marginal.	No EEG and no clinical application
Effects of three genres of focus music on heart rate variability and sustained attention	Kirk et al. (2022)	*n* = 120	There were performance differences across active music groups on the sustained attention to response task (SART) compared to the no-music control group. The study showed increased HRV response in the three active music groups compared to the no-music control group.	No EEG
Comparing the Effects of loving-kindness meditation (LKM), music, and LKM plus music on psychological well-being	Sorensen et al. (2019)	*n* = 78	Findings revealed that Convergence, Loving Kindness Meditation-only, and Music-only were equally effective interventions, producing small improvements in well-being.	No HR and no EEG
Effectiveness of deep breathing and body scan meditation combined with music to improve sleep quality and quality of life in older adults	Nanthakwang et al. (2020)	*n* = 59	Improvement in sleep quality following the intervention, whereas the control group showed no difference. There was a decrease in individual Pittsburgh Sleep Quality Index components such as sleep efficiency, perceived sleep quality, and daily disturbance.	No HR and no EEG

This summary reveals a gap in mechanistic studies that combine physiological data with psychometrics.

Music and mindfulness also impact neural oscillations measured by electroencephalography (EEG) (Lomas et al., 2015; Asif et al., 2019; Fachner et al., 2013; Maidhof et al., 2023). Depending on the context, music alters activity across frequency bands, including increasing theta, alpha, beta, and gamma frequencies (Pavlygina et al., 2004; Mahmood et al., 2022; Kučikienė and Praninskienė, 2018). Mindfulness can also modulate activity across alpha, beta, theta, and gamma frequencies. However, it is most consistently associated with elevated alpha and theta rhythms (Lomas et al., 2015; Chiesa and Serretti, 2010; Braboszcz et al., 2017; Cahn et al., 2013; Fell et al., 2010).

Despite evidence that music mindfulness can be an effective intervention for stress-related symptoms (Table 1), no studies to date have used heart rate measurements and EEG to systematically detail the physiological effects of a music mindfulness intervention. We hypothesize that music mindfulness has a synergistic effect on both sympathetic and parasympathetic activity, modulates a broader range of neural oscillations when combined, and reduces stress. We recruited adult non-experienced meditators with symptoms of depression and anxiety to participate in a two-week bilingual music mindfulness program, conducted either in a community-based location or virtually through a livestream. Sessions focused on “Focus” and “Motivation” and were facilitated by a mental health worker and a professional musician who improvised during the session. The Focus sessions guided participants in focused attention on their breath and body, while the motivation sessions targeted cognitive appraisal and restructuring, visualization, and open monitoring. We assessed the physiological impact of music mindfulness by utilizing mobile technology to record HRV and EEG from participants as they engaged in the sessions. All participants completed surveys regarding their stress levels, degree of mindfulness, state of consciousness, and level of social connection. Our data reveals the acute physiological and psychological effects of a community-based music mindfulness intervention for anxiety and depression.

## Materials and methods

### Ethical approval

This study was approved by the Yale Human Research Protection Program (HRPP), which includes the Institutional Review Board (IRB). These organizations ensure that research is conducted in accordance with laws, regulations, guidelines, and ethical principles, such as those outlined in the Belmont Report. The HRPP ensures that studies meet legal, regulatory, and ethical obligations and routinely collaborates with community partners to enhance quality, efficiency, and the protection of human participants (HIC #2000028866).

### Compliance statement

Yale University IRB is fully accredited by the Association for the Accreditation of Human Research Protection Programs (AAHRPP) and operates in accordance with applicable laws, regulations, and guidelines in the United States and other countries. This includes, but is not limited to, regulations from the United States Food and Drug Administration (FDA) (21 CFR 50 and 56), the U.S. Department of Health and Human Services (45 CFR Part 46), ICH Good Clinical Practice (ICH GCP), the Belmont Report, the World Medical Association Declaration of Helsinki, and the Council for International Organizations of Medical Sciences (CIOMS). Yale University IRB is registered with the FDA and OHRP, and its Federal Wide Assurance (FWA) is approved by OHRP. The IRB registration number listed below applies to all of Yale’s IRB panels.

IRB Registration #: 00011725.

IRB Organization (IORG) #: IORG0000431.

FWA#: 00002571.

### Participants

**Figure 1:**
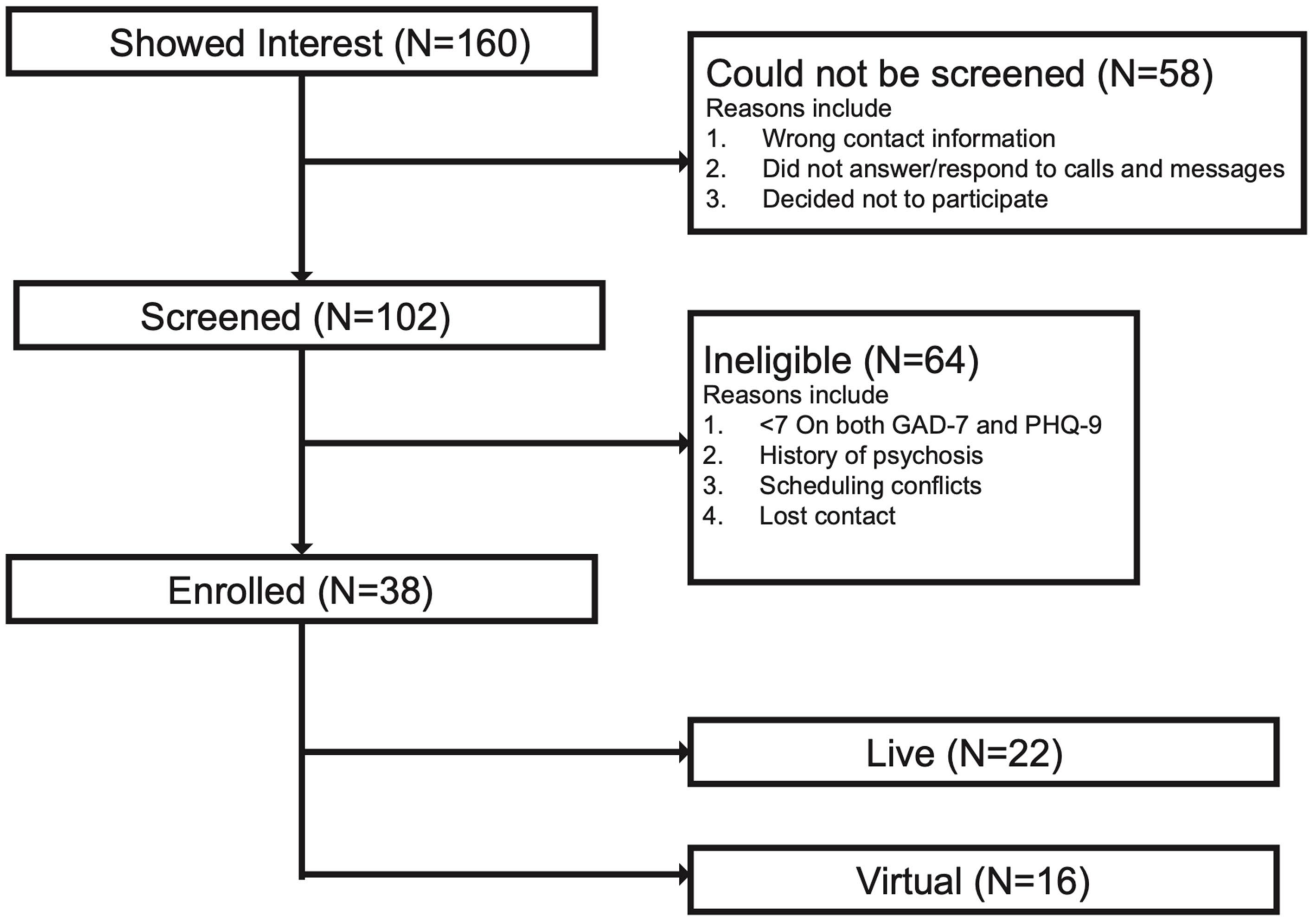
Study participant flowchart. The chart displays the number of individuals who were screened and enrolled. A total of 160 individuals were recruited. Of this number, 58 participants did not proceed with screening due to incorrect contact information, lack of follow-up response, or voluntary withdrawal from the study. Additionally, 64 participants were deemed ineligible to participate due to low anxiety and depression symptoms, evaluated by scores less than 7 for both GAD-7 and PHQ-9, a history of psychosis, scheduling conflicts, or loss of contact with the participant. Finally, a total of 38 participants were enrolled in the study: 22 participants in the live group (in-person musician and facilitator) and 16 participants in the virtual group (live-stream over laptop, phone, or tablet).

Using volunteer and convenience sampling, we partnered with community organizations to recruit 160 individuals for an in-person or virtual music mindfulness study. Convenience sampling was used to facilitate community-based recruitment, ensuring an ecologically valid and naturalistic study context. We screened 102 individuals from the New Haven community and enrolled 38 adults aged 18 to 65 years who had moderate symptoms of depression and anxiety. A total of 22 adults participated in the in-person component of the study, while 16 participated in the virtual component (Figure 1). Pre-consent screening included assessing the age and relevant medical history of each prospective participant. We utilized Yale University’s Clarity analysis platform to conduct a power analysis for our study. The power for a repeated measures ANOVA study with a sample size of 22, an alpha level of 0.05, and a large effect size (Cohen’s d = 0.8) is approximately 0.9460, indicating that our study has a 94.6% chance of detecting a large effect if it exists. This criterion exceeds our desired power level of 80%. The Patient Health Questionnaire-9 (PHQ-9) (Kroenke et al., 2001) and General Anxiety Disorder-7 (GAD-7) (Spitzer et al., 2006) were administered to participants after consent to determine eligibility. Participants who responded with a score of 7 or higher on the GAD-7 assessment were considered eligible. Those with a score of 9 or higher on the PHQ-9 questionnaire were also considered eligible. A confirmatory answer to the suicidality question on the PHQ-9 indicated ineligibility and the implementation of a Risk Reduction and Safety Plan. Following screening, verbal consent was obtained over the phone, and participants were enrolled in the 2-week study. Demographic information such as age, date of birth, gender, race and ethnicity was collected during the consent process. This demographic information aligned with the Yale Institutional Revisional Board (IRB) HIC #2000028866. During the consent process, significant emphasis was placed on the voluntary nature of the experiment. Both in-person and virtual participants were compensated based on their completion of surveys, agreement to wear the portable devices, and attendance at the optional focus group at the end of the study.

### In-person participant procedure

In-person participants were registered and assigned participant IDs to ensure anonymity. They were then required to complete a pre-survey before the experiment began, gathering background information, including age, gender, and place of residence. Participants reported any previous mental health diagnoses and past music engagement, then completed abbreviated versions of the Subjective Units of Distress Scale (SUDS), Perceived Stress Scale (PSS), Altered States of Consciousness (ASC) Scale, State Mindfulness Scale (SMS), and Social Connectedness Scale (SCS). Once the surveys were completed, the Polar H10 and Emotiv Insight EEG devices were fitted on the participants. No participants reported being left-handed. Data recording began, and the facilitators provided instructions on the recommended sitting position and expectations for the session. These instructions and mindfulness guidance were delivered in both English and Spanish to enhance understanding.

Participants were asked to relax for 1-min before listening to an informational excerpt from a book about mindfulness concepts, which served as the baseline recording and lasted 4-min. The start and end times of the recording were noted. After the baseline recording, a 50-s break was provided as a buffer between the baseline and the first part of the session. The instructions were read once again by the facilitator, followed by a 10-s countdown to indicate the start of the first session. The participants were then guided through either a “Focus” Meditation, which involved various focused attention exercises, or a “Motivation” Meditation, designed to enhance open monitoring, cognitive appraisal, cognitive restructuring, self-compassion, and arousal.

The first part of the session included either music or no music in the background, depending on the cohort group and the day. All parameters, including types of meditation, the order of music and no music, and the type of instrument used during the live meditation, were counterbalanced to avoid order effects. After completing the first part of the session, the participants kept their devices on but were given a 10-min break to complete a mid-session survey and drink water if needed. Participants were then instructed to take a seat and relax for a 1-min tolerance period. After this period, facilitators repeated the initial instructions to indicate that the second meditation was beginning. Another 10-s countdown was given by the facilitators before the second part of the session began. Participants were guided through this part, which was either music or no music, depending on the first session. Once the session ended, facilitators provided a 10-s countdown to indicate that the session was ending. Participants then removed their devices and completed the post-survey.

The in-person study focusing on wellness took place at BLOOM, a community center in New Haven, CT. Its retreat center is called the BLOOM Inn. Participants were either in a “live music room” with the musician and facilitator or in the “headphones room.” In the headphones room, they could listen to music and meditation in real-time through headphones while viewing visuals of the musician and facilitator. The preliminary survey and HRV data analysis did not reveal significant differences between groups, so they were analyzed together. All procedures were conducted in accordance with ethics approval from the Yale IRB.

### Virtual participant procedure

Virtual participants logged into a livestream link to participate in music mindfulness sessions in real time. After entering their name and ID, they were provided with instructions and a link to complete the pre-survey, which they filled out online. Once the pre-survey were completed, participants listened to a mixed livestream where they received the same instructions, tolerance guidelines, and transition periods. They then followed the guided instructions alongside the in-person participants. If participants had any questions, they could communicate during the stream via live chat. Similar to the in-person participants, virtual participants also completed a middle and post-survey. Virtual participants did not wear Polar H10 or mobile EEG devices and were analyzed solely to assess the impact of music mindfulness on psychometric data.

### Audio setup and engineering

Audio setup and engineering for music mindfulness sessions were provided by CT Streaming Services. A senior audiovisual engineer and an assistant were available to set up and break down after each session, which included running cables to speakers, headphones, cameras, and microphones. The audio was mixed and simultaneously projected to the room at BLOOM, in addition to the virtual participants via live stream. The audio mix included microphone input from the live instrumentalist, microphone input from the mindfulness facilitator, and a pre-recorded backing track.

### Music mindfulness session types

Participants took part in two types of Music mindfulness (MM) sessions: “Focus” and “Motivation.” Both were designed to help participants practice a set of mindfulness skills. The “Focus” MM sessions guided participants to focus on their bodies and states of mind, engaging in focused breathing from different points, including the nostrils and the belly. In the “Motivation” MM sessions, participants practiced cognitive appraisal and restructuring, visualization, self-compassion, and open monitoring of sensations and emotions. Each set of instructions was accompanied by a different music backing track, serving as the foundation for the live musician’s improvisation (see Supplementary Materials for the Focus and Motivation scripts).

The Focus meditation backing track was in the key of B Major and at 72 beats per minute (BPM). The Motivation backing track was in the key of E Minor and at 164 BPM. These backing tracks were initially created based on music parameters identified as supportive of mindfulness (Focus) and for supporting arousal (Motivation) (Dvorak and Hernandez-Ruiz, 2021; Hernandez-Ruiz and Dvorak, 2021; Carpentier et al., 2007; Salimpoor et al., 2009; Husain et al., 2002). The Focus backing track was selected because it was a preferred track from our feasibility study of a virtual music mindfulness platform (Igbinobaro et al., 2024). Music tracks are available to listen to at https://on.soundcloud.com/Yyv34iPa8UnQroxz8.

Each session consisted of a period with background music and a live musician improvising along with the mindfulness facilitator, as well as a period with mindfulness instructions without music. Both period lasted 15 min and had the same mindfulness instructions. The order of the music versus no-music sessions was counterbalanced to control for the potential temporal effect of the music order.

### Live musicians

Two professional musicians, a cellist and a trumpet player, were selected in collaboration with the New Haven Symphony Orchestra. They received the backing track audio before the music mindfulness sessions. During the session, the musicians improvised alongside the backing track. The two instruments were chosen to provide live sound with varying temporal and spectral energy distributions, temporal variations, and different timbres (Wessel, 1979; Thoret et al., 2021).

### Audio spectrograms

Audio spectrograms were generated from the last 13 to 17-s of both Focus and Motivation mindfulness sessions, with and without music, during which the facilitator says, *“If* *the eyes were closed, beginning to open them or lifting the gaze. Take your time. Si los ojos están cerrados comience a abrirlos o a levantar la mirada. Tome su tiempo.” The* audio channels of the recording samples were converted from stereo to mono, and the file format was changed from .mp4 to .wav. The matplotlib spectrogram function was used to generate spectrograms for the four audio samples, applying a Fast Fourier Transform (FFT) to the audio signals. All spectrograms were generated at a sampling rate of 48,000 Hz.

## Physiological measurements

### Heart rate data acquisition

The Polar H10 sensor chest strap (Polar Electro Oy, Kempele, Finland) was paired via Bluetooth with the Polar Flow App (Version 6.24.0 (1030)) to record participants’ heart rate (HR) data at a sampling rate of 1,000 Hz. The device uses electrical sensors to measure electrical activity generated by the heart muscle when it contracts. Participants wore the device under their garments to ensure direct skin contact. HR data of all 22 in-person participants was recorded during both Focus and Motivation MM sessions.

### Heart rate variability (HRV) data processing

Heart rate variability (HRV) was measured according to the recommendations of the Task Force of the European Society of Cardiology and the North American Society of Pacing and Electrophysiology (Malik et al., 1996). HRV was calculated to assess the parasympathetic and sympathetic activity of the autonomic nervous system. The raw HR data for each participant was extracted from the Polar Flow web app as a Comma-Separated Values (CSV) file, and the periods of interest were noted for all data files. The R-R intervals were calculated for each HR data point recorded during the baseline, music, and no music periods, using the formula 60,000/HR to convert beats per minute (BPM) into seconds (Young, 2014). Linear interpolation was applied in the forward direction on participants’ data with less than 20% missing data points, while those with more missing data were excluded. Time and frequency domain HRV parameters were computed using the hrv-analysis Python package.^1^ The computed time domain parameters included the standard deviation of the normal-normal intervals (SDNN) and the root mean square of successive interval differences (RMSSD). Very low-frequency power (VLF) and the ratio of low-frequency to high-frequency powers (LF/HF) were also calculated for frequency domain parameters. The HRV parameters were computed for the entire session and for 5-min durations to assess dynamics throughout the session, in addition to being computed for the 4-min baseline period.

### Heart rate variability (HRV) analysis

HRV was analyzed, taking into consideration the normality of the data. GraphPad Prism was used to generate all plots and conduct statistical analyses. Bar plots were generated for the 5-min and 15-min intervals to compare the effects of baseline, music, and no-music sessions during focus and motivation meditations on the time and frequency domain HRV parameters. Additionally, bar plots were generated for both male and female participants during 5-min intervals to assess the impact of sex differences on the autonomic response to the MM sessions. Statistical analysis for the 5-min duration bar plots was performed using two-way ANOVA with Greenhouse–Geisser correction to ensure the accuracy of results when the sphericity assumption is violated. One-way ANOVA was used for statistical analysis on the 15-min duration bar plots. Repeated measures ANOVA based on the general linear model was applied for both one-way and two-way analyses. Post-hoc analysis on bar plots was conducted using Tukey’s multiple comparisons test. Line graphs were generated from the 1-min interval computed parameters. A simple linear regression was used for the statistical analysis of the line graphs.

### Electroencephalography (EEG) data acquisition

EEG data was recorded using portable EEG headsets (Emotiv Insight) that could be easily deployed in a real-world community setting. EEG signals were recorded from five channels (AF3, AF4, T7, T8, Pz) at a sampling rate of 128 Hz, with a resolution of 0.1275 μV, and included built-in filtering, such as digital notch filters at 50 Hz and 60 Hz, along with a built-in digital 5th order Sinc filter. The bandwidth frequency response ranged from 0.5 to 45 Hz. Two reference channels were positioned on the left mastoid process, including the CMS/DRL references. Three-prong gummy sensors were also included on the Pz channel to ensure better penetration through hair. A 10–20 semi-dry polymer sensor configuration was used, which included Pz. A bottle of “Primer Fluid” consisting of 80% glycerin and 20% saline was applied to each sensor tip to improve contact with the scalp and improve signal quality.

Once the headset was placed on the participant, the contact quality of the sensors was measured using the EmotivPRO app. A sensor map displayed four colors indicating different states: black for no contact, red for poor contact, yellow/orange for average contact, and green for good contact quality. If low contact quality was observed, the participant’s hair was adjusted using a small lighted fiber optic probe (Hiroshima, Japan) to improve contact with the scalp. Once the contact quality reached 100%, the EEG quality was assessed. To assess EEG quality, a map of the electrodes with varying colors was shown on the EmotivPRO app, corresponding to the EEG signal quality for each sensor. The score ranged from 0 to 100, with scores closer to 100 representing higher quality. To improve EEG quality, the reference electrodes near the left ear of participants were readjusted, with the optimal position being against the mastoid bone. Sensors indicating poor EEG quality were then adjusted, additional primer fluid was applied to the sensors, and hair was manipulated if necessary. The EmotivPro software was utilized to obtain a live recording of the EEG signal. Signals from all five channels were collected to analyze EEG signals across delta (0.5–4 Hz), theta (4–8 Hz), alpha (8–12 Hz), beta (13–27 Hz), and gamma (28–40 Hz) frequency bands. The signals were pre-processed using custom code written with MNE Python.

### EEG pre-processing and analysis pipeline

Initially, the raw data were visually inspected to identify any corrupted or missing segments. Participant data with 50% or more missing values were automatically excluded from analyses. We developed a preprocessing and analysis pipeline using Python and the MNE library (version 1.6.1), informed by the Emotiv Analyzer framework. Slew rate limiting was performed with a threshold of 30 Hz to control the maximum rate of signal change, mitigate amplifier saturation, and reduce artifacts. Signals were re-referenced to the interquartile mean to minimize the influence of outlier channels and artifacts, ensuring a more robust estimation of the signal’s central tendency. The EEG data were then filtered using a band-pass filter with a low cutoff set at 0.5 Hz and a high cutoff at 45 Hz to remove low-frequency drifts and high-frequency noise. An automatic Independent Component Analysis (ICA) was applied to remove ocular artifacts. ICA was computed to find numerous components that explain 99% of the variance in the data. We designated channels close to the eyes as electrooculogram (EOG) channels and used the MNE library to compute correlations between each IC and EOG channels to determine which ICs are responsible for ocular artifacts. We find the threshold correlation score by iterating over a range of threshold values starting from 3.5 and stepping down until at least two ICs are identified. This is because we expect to identify both blinks and eye movement artifact ICs. Finally, the preprocessed EEG data were transformed into power spectral density (PSD) values across Delta, Theta, Alpha, Beta, and Gamma frequency bands using Fast Fourier Transform (FFT). For FFT, the Welch method was used with a window length of 512 samples, representing 4-s of data, and a step size of 128 samples.

### Representative spectrograms

To visualize the temporal evolution of power across different frequency bands, time-frequency spectrograms were computed using custom Python code leveraging the SciPy Python library (version 1.12.0). A value of 1,028 was selected as the length for each sample for the Short-Time Fourier Transform (STFT), with an overlap of 128 samples.

### Topographic maps

Topographic maps were created using the MNE Python library (version 1.6.1) to visualize the spatial distribution of EEG power spectral density (PSD) across the scalp. The average PSD for each frequency band (Delta, Theta, Alpha, Beta, and Gamma) and electrode (AF3, AF4, T7, T8, and Pz) was computed for all participants. Therefore, each topographic map represents the average PSD among participants. These maps provide a clear depiction of condition-specific neural activity patterns, highlighting regional differences in EEG power dynamics across sessions.

### Average PSDs

Measurements were collected from 18 participants for focus meditation and 20 participants for motivation during both 15-min meditation periods. The average power spectral density (PSD) was calculated for the entire 15-min period under three conditions: music, no music, and baseline for both meditation types. The average PSD for all participants was presented as a mean ± standard error of the mean. A Friedman’s test was conducted to assess differences in average PSD across all conditions (baseline, music, no music) using MNE Python and the SciPy Python library. Analyses were also performed for male and female subgroups.

### PSD Normalization

To account for the observed individual variability in EEG responses, data were normalized. Normalization was performed by first calculating the PSD values for each condition (Baseline, Music, No Music). For each participant, the average PSD value for the Music and No Music conditions was calculated for each frequency band and then divided by the average PSD value from their Baseline condition, as shown in Equation 1, where the condition can either be music or no music EEG data.


(1) $$\mathrm{Normalized}\;PSD_{\mathrm{condition}\;\mathrm{frequency}\;\mathrm{band}}=\frac{{\bar{PSD}}_{\mathrm{condition},\mathrm{frequency}\;\mathrm{band}}}{{\bar{PSD}}_{\mathrm{baseline},\mathrm{frequency}\;\mathrm{band}}}$$

This approach ensures that the data reflects relative changes in EEG activity, minimizing inter-individual differences in baseline EEG power.

### Psychometric measurements

The psychological impact of the music mindfulness session was measured through surveys. To facilitate participation in a community-based setting, and based on preliminary feasibility focus groups in the local community (New Haven, CT), we used abbreviated versions of the Subjective Units of Distress Scale (SUDS), Perceived Stress Scale (PSS 10) (Cohen, 1988), Altered States of Consciousness Scale (ASC) (Studerus et al., 2010; Schmidt and Berkemeyer, 2018), State Mindfulness Scale (SMS) (Tanay and Bernstein, 2013) and Social Connectedness Scale (SCS) (Lee and Robbins, 1995). The surveys were administered weekly before the session, during both parts of the session, and post-session.

#### Subjective units of distress assessment

Subjective Units of Distress Scale (SUDS) ratings are commonly used during exposure tasks in cognitive behavioral treatment (CBT) for anxiety and PTSD (Morey et al., 2016; Newman and Kaloupek, 2004; Benjamin et al., 2010). It can also serve as a community-based assessment to indicate the level of emotional distress in individuals at risk for major stress-related causes of psychiatric hospitalizations (Kent and Yellowlees, 1994). We utilized a five-question SUDS to capture overall levels of stress and major risk factors for hospitalization. It was administered prior to and following the music mindfulness sessions and was scored on a scale from 1 (lowest level of distress) to 7 (highest level of distress). Cronbach’s alpha was calculated using Yale University’s Clarity analysis platform and was found to be approximately 0.70. This suggests that the items demonstrate acceptable internal consistency and are reliable for measuring the underlying construct of distress.

##### SUDS

On a scale of 1 (low) to 7 (high), please select how intensely you are currently experiencing the following feelings and urges.

- Level of stress.
- Urge to harm yourself.
- Intent to end your life.
- Urge to use drugs or alcohol.
- Urge to harm someone else.

#### Perceived stress scale

In the PSS questionnaire, each question is scored on a scale from 0 (Never) to 4 (Very often). A 5-item version of the PSS-10 was administered in the pre-survey and post-survey to assess changes in perceived stress throughout the intervention. PSS evaluates how situations in one’s life are perceived as stressful. The psychometric properties of the PSS-10 were initially evaluated in a large national sample of 2,387 American adults (Cohen, 1988). Scores on the PSS-10 demonstrated adequate internal consistency reliability (*α* = 0.78), moderate concurrent criterion validity with the amount of stress experienced during an average week (*r* = 0.39, *p* < 0.001), and the frequency of stressful life events in the past year (*r* = 0.32, *p* < 0.001); they also showed adequate convergent validity, evidenced by expected negative associations with perceived health status (*r* = −0.22, *p* < 0.001) and positive associations with psychosomatic symptoms (*r*s = 0.28 to 0.34, *p* < 0.001) (Baik et al., 2019). The Cronbach’s alpha for the shortened version of the PSS scale is 0.73, indicating an acceptable level of internal consistency and reliability for measuring perceived stress in the sample.

##### PSS

Select the circle that best represents how often you felt or thought a certain way.

- In the last month, how often have you been upset because of something that happened unexpectedly?
- In the last month, how often have you felt that you were unable to control the important things in your life?
- In the last month, how often have you felt nervous and “stressed”?
- In the last month, how often have you found that you could not cope with all the things that you had to do?
- In the last month, how often have you been able to control irritations in your life?

#### The state mindfulness scale

The SMS questionnaire was utilized to assess the enhancement of present-moment awareness following an intervention. Participants rated their experiences on a Likert scale ranging from 1 (Not at all) to 5 (Very good). SMS evaluates the effects of mindfulness training within the context of our music mindfulness-based sessions. From the original 21-item scale, we selected six items that focused on key aspects of subjective awareness, including mindfulness of bodily sensations and mental events. These items reflected the integration of traditional Buddhist and contemporary definitions of mindfulness that underpin the SMS model. Higher SMS scores suggest an enhancement in mindfulness. The SMS achieved a Cronbach’s Alpha of 0.88, indicating that the items in the shortened version of the SMS scale are highly reliable and consistent in measuring the intended construct of state mindfulness.

##### SMS

Please indicate how much you have been currently experiencing each of the following by selecting one of the options presented below.

- I was aware of different emotions that arose in me.
- I noticed emotions come and go.
- I noticed thoughts come and go.
- It was interesting to see the patterns of my thinking.
- I felt present in my body.
- I noticed the sensations in my body.

#### The altered states of consciousness scale

The ASC measures marked deviations in the subjective experience or psychological functioning of an individual from their usual waking consciousness. These states are typically self-induced (e.g., through hallucinogenic drugs, meditation, or hypnosis), but they may also occur spontaneously in everyday life (e.g., during hypnagogic states) (Studerus et al., 2010; Schmidt and Berkemeyer, 2018). The Altered States of Consciousness Scale (ASC) is a 94-item questionnaire designed to assess altered states of consciousness in psychedelic medicine (Dittrich, 2007). In this study, we first used multiple focus groups to condense the scale by selecting questions that represented each of the five original dimensions. The Cronbach’s alpha for the dataset is approximately 0.91, indicating an excellent level of internal consistency and reliability for the scale.

##### ASC

Below, you will find a number of statements, and next to each statement, there is a line with the endpoints “No, not more than usual” on the most left and “Yes, much more than usual” on the right. The line functions similarly to a thermometer, measuring alterations in your normal consciousness while awake or how much your state of consciousness has deviated from your normal state. Please rate the extent to which the statements apply to your current experience by adjusting the slider on the line below. Please note: A mark at the far left end of the scale represents your normal waking consciousness, indicating “No, my state of consciousness has not changed at all compared to normal,” while a mark at the far right end means, “Yes, my state of consciousness has changed the most compared to normal.”

- My thoughts and actions were slowed down.
- Bodily sensations were very enjoyable.
- Sounds seemed to influence what I saw.
- Some everyday things acquired special meaning.
- I felt one with my surroundings.
- Worries and anxieties of everyday life felt unimportant.
- My sense of time and space was altered as if I was dreaming.
- I experienced profound inner peace.
- I experienced an all-embracing love.

#### The social connectedness scale

The SCS is a 20-item instrument designed to assess various dimensions of social connectedness, including belongingness, closeness, support, and satisfaction. We selected four items that specifically address the support dimension, focusing on participants’ perceptions of having individuals to rely on for help and understanding. Participants rated each item on a 6-point Likert scale, ranging from 1 (strongly disagree) to 6 (strongly agree). Scores for the SCS are calculated by summing the ratings for all items, with higher scores indicating a greater sense of social connectedness. The SCS evaluates participants’ sense of social support and connectedness before and after sessions. The Cronbach’s alpha for the dataset is approximately 0.88, indicating a high level of internal consistency and reliability for the scale.

##### SCS

Click the answer that shows how much you agree or disagree with each of the following statements.

- I feel disconnected from the world around me.
- Even around people I know, I do not feel that I really belong.
- I feel so distant from people.
- I catch myself losing all sense of connectedness with society.

The middle survey included questions about individuals’ levels of social connectedness, states of mindfulness, and altered states of consciousness. The post-survey posed the same questions as the pre-survey.

### Psychometric analysis

Psychometric data were analyzed by calculating the average of all participants’ pre-survey scores and the average of all post-survey scores. Additionally, the average difference between participants’ pre-and post-survey scores (post-survey score minus pre-survey score) was calculated to assess the impact of focus and motivation sessions. These analyses were conducted for both live and virtual participants. The impact of the mindfulness intervention was evaluated by comparing average pre-and post-survey scores, as well as pre-post difference scores among all live male and female participants during focus and motivation sessions. To compare the differences between live and virtual sessions, the average pre-post difference scores were assessed for both live and virtual participants. The varying participant numbers in live and virtual settings resulted in unequal sample sizes, which was addressed by conducting the Levene test using SciPy in Python to test for homogeneity of variance. The appropriate statistical tests for the psychometric data were then identified based on normality and homogeneity of variances. GraphPad Prism was used to create bar graphs with standard error of the mean (SEM) error bars and to perform statistical analyses. In graphs comparing pre-and post-survey scores, paired statistical tests were applied, whereas unpaired statistical tests were utilized for graphs comparing pre-post difference values in focus versus motivation sessions. Some analyses were performed using the “SciPy” or “statsmodel” Python package.

### Correlation of HRV metrics and psychometric scores

Correlation analysis was conducted on sessions with significant HRV metrics and significant psychometric scores using GraphPad Prism. In this process, the average SDNN, RMSSD, and VLF during the baseline period were subtracted from the average SDNN, RMSSD, and VLF recorded in the last 5-min of the focus session. This change was then correlated with the changes in SUDS, ACS, SMS, and SCS psychometric scores. The analysis aimed to assess how the psychometric scores reported by participants relate to their autonomic responses during the focus session. Statistical analysis was conducted using simple linear regression on GraphPad Prism to compute the goodness of fit and the significance of the slope parameters.

## Results

**Figure 2:**
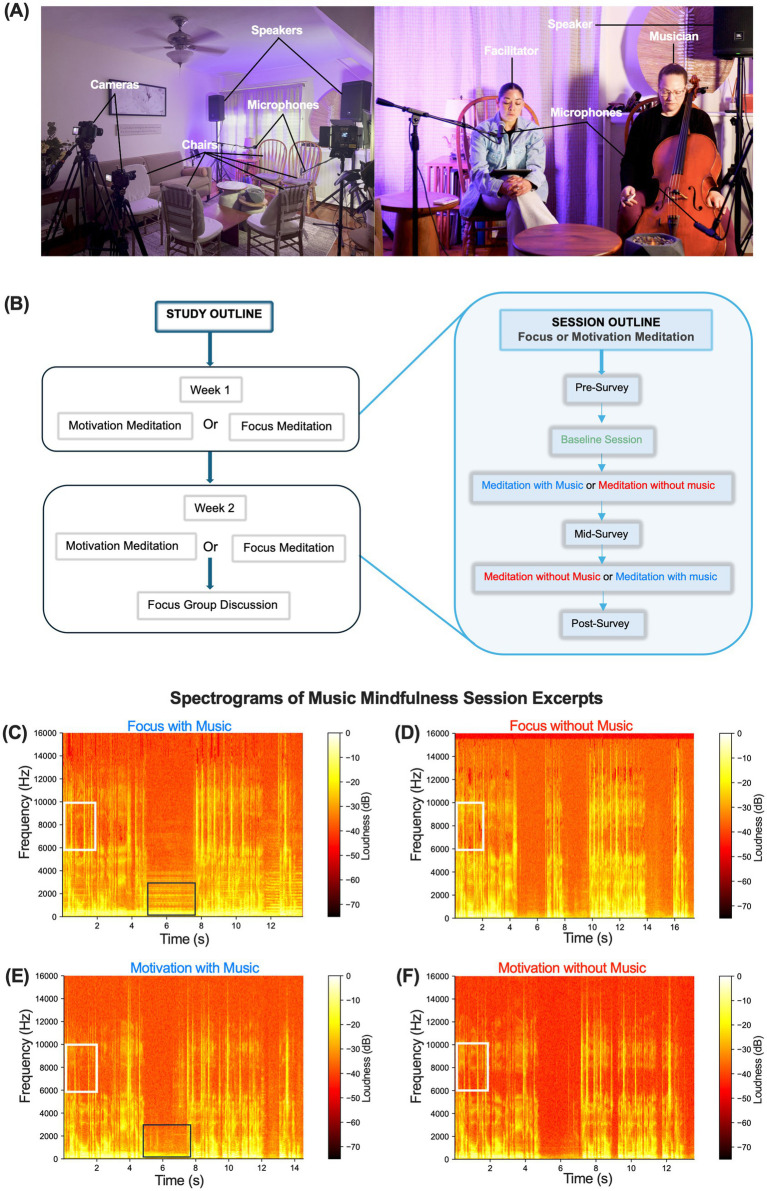
A 2-week community-based music mindfulness program. **(A)** Left-music mindfulness sessions are set up with streaming cameras, microphones, speakers, and soft ambient lighting, with chairs arranged for participants, facilitators, and musicians. Right—front view of the session setup. **(B)** Study and session outline. Participants enrolled in a 2-week music mindfulness study with focus and motivation sessions. All participants experienced both sessions, each consisting of a section with and without music. **(C–F)** are representative time-frequency spectrograms from high-fidelity audio recordings of Focus and Motivation sessions, both with and without music. They show the last 13–17-s of each session when the facilitator says, *“If* *the eyes were closed, beginning to open them, or lifting the gaze. Take your time. Si los ojos están cerrados, comience a abrirlos o a levantar la mirada. Tome su tiempo.”* Boxes show representative instrumental (black box) and vocal (white box) formants during the session.

Eligible individuals with moderate symptoms of anxiety and depression (n = 38) participated in two weeks of guided bilingual music mindfulness sessions focused on “Focus” and “Motivation” (Table 2; Figure 1). The sessions were conducted at a local community center in a room fully equipped with audio capabilities for a live musician, facilitator, and multiple participants (Figure 2A). The sequence of session types (Focus or Motivation) was counterbalanced across weeks (Figure 2B). Each session included a section with music where the live musician improvised in accordance with the facilitator’s guided instructions, as well as another section with guided instructions without music (Figure 2B). The mindfulness instructions remained the same during both sections, and the order of sections was counterbalanced across sessions (Figure 2B).

**Table 2:** Demographic and mental health assessments of participants: patient health questionnaire-9 (PHQ-9) and generalized anxiety disorder-7 (GAD-7).

		Live group (*n*) = 22	Virtual group (*n*) = 16	Total (*n*) = 38	Percentage (%)
Sex/gender	Male	7	3	10	26
	Female	15	13	28	74
Race/ethnicity	Asian	4	1	5	13
	Black or African American	10	10	20	53
	Hispanic	1	1	2	5
	Latin	2	3	5	13
	Multiracial	2	0	2	5
	White	3	1	4	11
Age (years)	18–34	21	10	31	82
	35–64	1	6	7	18
PHQ-9	Mean ± STD	12.64 ± 4.80	10.73 ± 4.06	11.865 ± 4.565	
GAD-7	Mean ± STD	9.42 ± 3.93	9.00 ± 3.72	9.25 ± 3.79	
Modified AIMS (Total = 85)	Mean ± STD	60.222 ± 15.326	62.778 ± 5.619	61.074 ± 12.838	

Participant demographics and clinical scores are summarized, including sex/gender, racial background, age (in years), and mental health assessments. Sex/gender, Race/ethnicity, and Age are reported as numbers and percentages. Sex/gender and Race/ethnicity are categorized based on self-reported identification. Age is grouped into two categories: 18–34 and 35–64 years. PHQ-9 and GAD-7 scores are presented as means ± standard deviation, indicating levels of depressive and anxiety symptoms, respectively. PHQ-9 and GAD-7 scores are calculated for a total of 37 and 32 participants, respectively. The Modified AIMS (absorption in music scale) is also presented as means ± standard deviation, capturing music’s ability to immerse participants in an emotional experience. The Modified AIMS consists of 17 questions instead of the original 34 questions.

**Figure 3:**
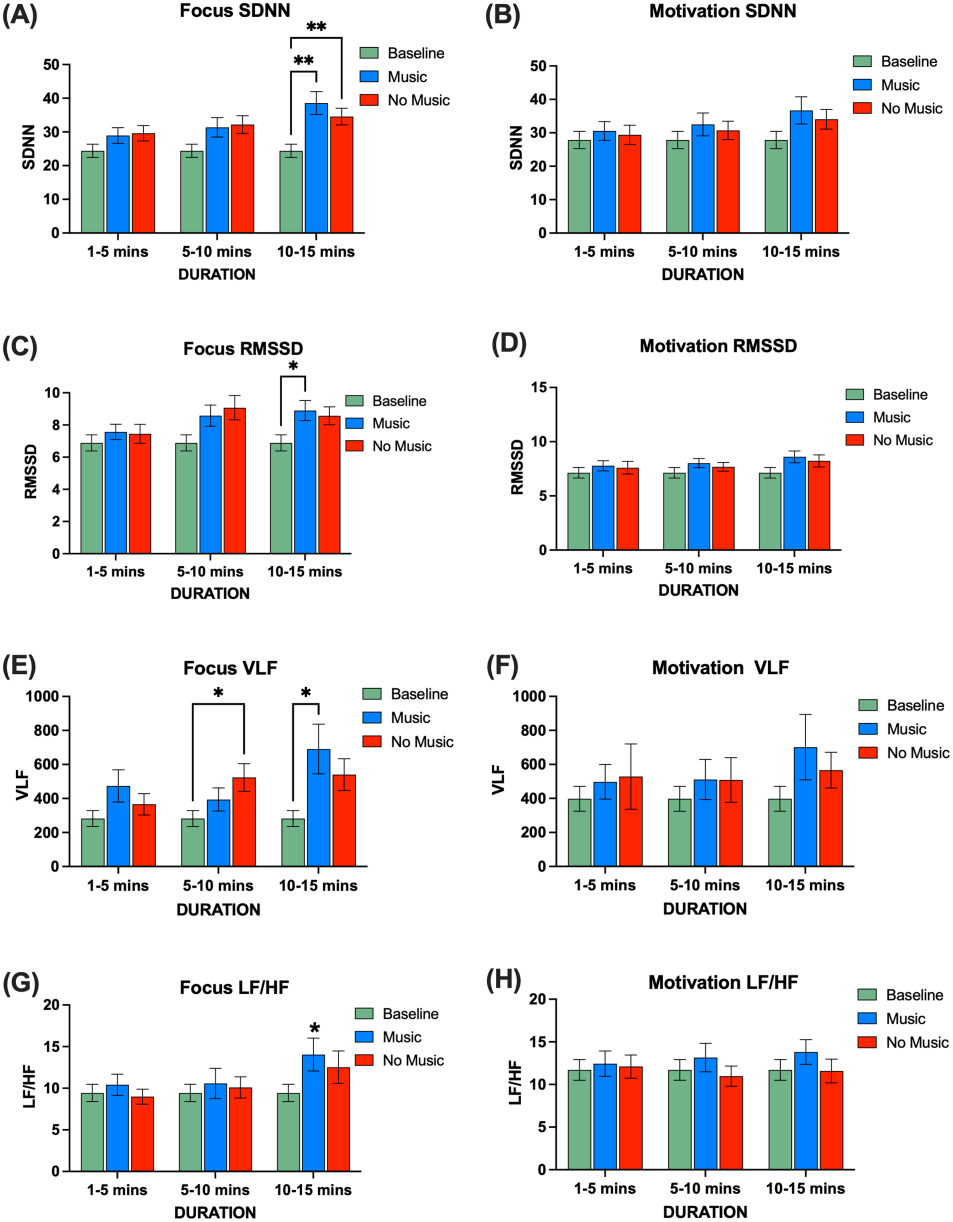
Comparison of time and frequency domain HRV metrics during focus (*n* = 19) and motivation (*n* = 21) sessions, represented in 5-min time windows. **(A)** SDNN during focus was significantly greater than baseline for both music and no music. A two-way repeated measures ANOVA revealed significant effects in Time × Session (F(4, 108) = 3.480, *p* = 0.0103), Time (F(1.971, 106.4) = 9.845, *p* < 0.0001), Session (F(2, 54) = 4.580, *p* = 0.0145), and Participants (F(54, 108) = 7.831, *p* < 0.0001). Tukey’s *post hoc* multiple comparisons tests showed significant differences for music and no music between minutes 10 and 15 (q = 5.122; **p = 0.0031, q = 4.570; **p = 0.0075). **(B)** SDNN during motivation: a two-way repeated measures ANOVA showed significant effects for Time (F(1.632, 97.93) = 4.086, *p* = 0.0269) and Participants (F(60, 120) = 8.786, *p* < 0.0001). **(C)** RMSSD during focus; a two-way repeated measures ANOVA showed a significant difference in Time × Session (F(4, 108) = 4.587, *p* = 0.0018), Time (F(1.582, 85.44) = 14.66, *p* < 0.0001), and Participants (F(54, 108) = 18.56, *p* < 0.0001). Tukey’s *post hoc* multiple comparisons tests showed a significant difference between minutes 10 and 15 for music (q = 3.531; *p = 0.0450). **(D)** RMSSD during motivation: a two-way repeated measures ANOVA showed significant differences in Time (F(1.765, 111.2) = 4.828, *p* = 0.0127) and Participants (F(63, 126) = 15.49, *p* < 0.0001). **(E)** VLF during focus; a two-way repeated measures ANOVA showed significant differences for Time × Session (F(4, 108) = 3.893, *p* = 0.0054), Time (F(1.752, 94.63) = 5.744, *p* = 0.0062), and Participants (F(54, 108) = 6.380, *p* < 0.0001). Tukey’s *post hoc* multiple comparisons tests showed significant differences between minutes 5 to 10 for no music and 10 to 15 for music (q = 3.641; *p = 0.0398, q = 3.758; *p = 0.0371). **(F)** VLF during motivation; a two-way repeated measures ANOVA showed significant differences in participants (F(60, 120) = 5.348, *p* < 0.0001). **(G)** LF/HF during focus; a two-way repeated measures ANOVA showed significant differences in Time (F(1.930, 104.2) = 6.701, *p* = 0.0021) and Participants (F(54, 108) = 6.423, *p* < 0.0001). **(H)** LF/HF during motivation; a two-way repeated measures ANOVA showed significant differences in participants (F(60, 120) = 6.025, *p* < 0.0001). SDNN: standard deviation of the normal-normal intervals, RMSSD: root mean square of successive interval differences. VLF: very low-frequency power; LF/HF: ratio of very low-frequency power to very high-frequency power. Error bars denote the standard error of the mean, SEM.

Spectrograms of audio recorded during the sessions revealed a similar range of frequencies from 0.1 Hz to 15,000 Hz across all sessions (Figures 2C–F). Sessions featuring music showed characteristic formants associated with music production, while all sessions displayed distinct vocal formants (Figures 2C–F). We recorded HR activity throughout the music, noting that the only section in the music mindfulness session preceding the baseline involved participants listening to a mindfulness reading with their eyes closed. We analyzed HR alongside time and frequency domain HRV metrics. No changes in average HR were observed compared to baseline during focus or motivation sessions (Supplementary Figure 1). Analyzing time domain HRV metrics, we found a significant increase in the standard deviation of normal-normal intervals (SDNN) during the last 5 min of the Focus session under both music and no music conditions (two-way repeated measures ANOVA, Time × Session) (F(4, 108) = 3.499, *p* = 0.0100), Time (F(1.965, 106.1) = 10.40, *p* < 0.0001); Tukey’s *post hoc* multiple comparisons tests showed significance for music (q = 5.122; **p = 0.0031) and no music (q = 4.871; **p = 0.0042) between minutes 10 and 15 (Figure 3A; Supplementary Figure 2A). We did not observe a significant increase during the Motivation sessions (Figure 3B). A significant increase in the root mean square of successive interval differences (RMSSD) was noted during the music condition in the last 5 min (two-way repeated measures ANOVA, Time × Session) (F(4, 108) = 4.587, *p* = 0.0018), Time (F(1.582, 85.44) = 14.66, *p* < 0.0001), and Participants (F(54, 108) = 18.56, *p* < 0.0001); Tukey’s *post hoc* multiple comparisons test between minutes 10 and 15 for music (q = 3.531; *p value = 0.0450) (Figure 3C). A similar significant increase in RMSSD was not observed during the Motivation session (Figure 3D). Examining frequency domain HRV metrics, we discovered that very low-frequency power (VLF) significantly increased during no music after the first 5 min and during music in the last 5 min (two-way repeated measures ANOVA, Time × Session) (F(4, 108) = 3.893, *p* = 0.0054); Tukey’s *post hoc* multiple comparisons test revealed significance between minutes 5 and 10 for no music (q = 3.788; *p = 0.0314) and minutes 10 and 15 for music (q = 3.758; *p = 0.0371) (Figure 3E). We did not detect a statistically significant increase in VLF during motivation (Figure 3F). We also assessed the ratio of very low-frequency power to very high-frequency power (LF/HF), observing a statistically significant effect of time on LF/HF during Focus (two-way repeated measures ANOVA), Time (F(1.922, 103.8) = 6.296, *p* = 0.0030), but not during Motivation sessions (Figures 3G,H).

We analyzed our HRV data to determine whether there were differences based on participants’ reported sex. During Focus sessions, males demonstrated a significantly higher SDNN during the last 5 min of the no music condition compared to baseline (two-way repeated measures ANOVA), Participants (F(18, 36) = 29.96, *p* < 0.0001); Tukey’s *post hoc* multiple comparisons test for no music between minutes 10 and 15 (q = 4.706; *p = 0.0203) (Supplementary Figure 3A). Interestingly, we found that females also exhibited a significant increase in SDNN during Focus music mindfulness compared to baseline (two-way repeated measures ANOVA, Time × Session) (F(4, 72) = 3.379, *p* = 0.0137), Time (F(1.856, 66.81) = 6.044, *p* < 0.0047), and Participants (F(36, 72) = 5.922, *p* < 0.0001). Tukey’s *post hoc* multiple comparisons test between minutes 10 and 15 for music (q = 4.299; *p = 0.0190) (Supplementary Figure 3A). Additionally, females showed a significant increase in RMSSD during Focus music mindfulness that was not observed in males (two-way repeated measures ANOVA, Time × Session) (F(4, 72) = 6.125, *p* = 0.0003), Time (F(1.726, 62.15) = 12.61, *p* < 0.0001), and Participants (F(36, 72) = 14.88, *p* < 0.0001). Tukey’s *post hoc* multiple comparisons test between minutes 10 and 15 for music (q = 4.108; *p = 0.0251) (Supplementary Figure 3C). During the motivation sessions, females exhibited a significantly higher RMSSD during music mindfulness that was not seen in males (two-way repeated measures ANOVA, Time × Session) (F(4, 78) = 2.627, *p* = 0.0407), Time (F(1.585, 61.81) = 9.684, *p* = 0.0006), and Participants (F(39, 78) = 9.231, *p* < 0.0001). Tukey’s *post hoc* multiple comparisons test between minutes 10 and 15 for music (q = 3.545; *p = 0.0484) (Supplementary Figure 3D).

**Figure 4:**
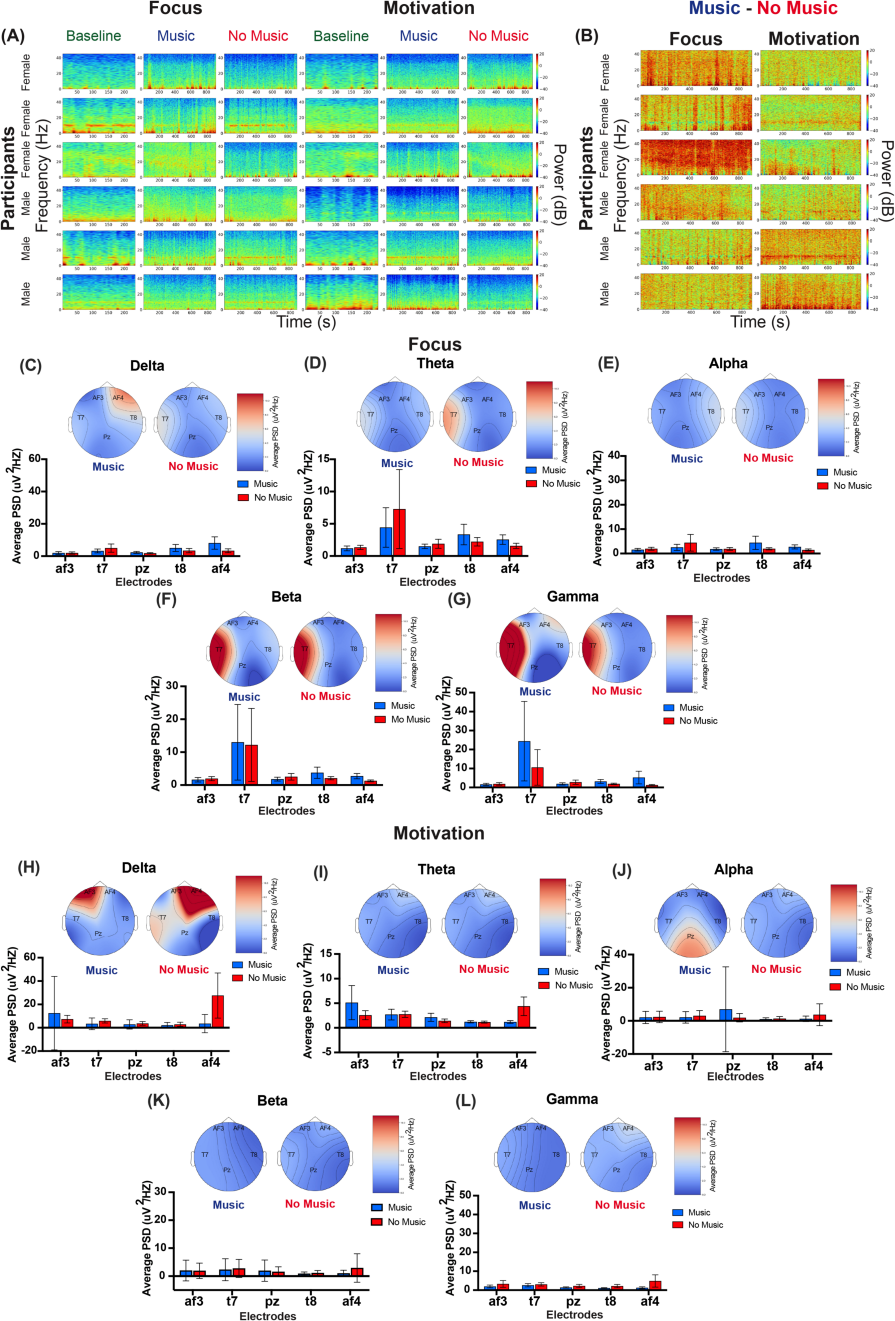
EEG power spectrum across frequency bands during music mindfulness. **(A)** Representative time-frequency EEG spectrograms illustrating power spectral density (PSD) across frequencies over time during baseline, music, and no-music sessions for both Focus and Motivation conditions. Each row represents one participant, totaling six participants. The first three participants are female participants, while the next three are male participants. **(B)** Different EEG spectrograms illustrate the contrast between music and no-music conditions (music–no music) for the same participants. Each spectrogram visualizes the differential PSD, highlighting frequency-specific changes in brain activity influenced by the music condition. Warmer colors (red) indicate increased power, while cooler colors (blue) indicate decreased power in the music condition compared to no music. **(C–L)** Group-averaged power spectral density (PSD) and EEG topographic maps for the Focus and Motivation conditions based on normalized EEG data. **(C)** Delta frequency band for focus music and no-music sessions. The Friedman test did not show a significant difference in PSD across electrodes during music sessions, χ^2^ = 6.000, *p* = 0.1991. Similarly, there was no significant difference in PSD across electrodes during No-Music sessions, χ^2^ = 4.2667, *p* = 0.3711. **(D)** Theta frequency band for focus music and no-music sessions. The Friedman test did not show a significant difference in PSD across electrodes during music sessions, χ^2^ = 6.1333, *p*-value = 0.1894. Similarly, there was no significant difference in PSD across electrodes during No-Music sessions, χ^2^ = 4.5333, *p*-value = 0.3386. **(E)** Alpha frequency band for focus music and no-music sessions. The Friedman test did not show a significant difference in PSD across electrodes during Music sessions, χ^2^ = 3.1557, *p*-value = 0.5321. Similarly, there was no significant difference in PSD across electrodes during No-Music sessions, χ^2^ = 2.3555, *p*-value = 0.6707. **(F)** Beta frequency band for focus music and no-music sessions. The Friedman test did not show a significant difference in PSD across electrodes during Music sessions, χ^2^ = 4.9333, *p* = 0.2942. Similarly, there was no significant difference in PSD across electrodes during No-Music sessions, χ^2^ = 3.7777, *p* = 0.4369. **(G)** The gamma frequency band for focus music and no-music sessions. The Friedman test did not show a significant difference in PSD across electrodes during Music sessions, χ^2^ = 6.0444, *p*-value = 0.1959. There was no significant difference in PSD across electrodes during No-Music sessions, χ^2^ = 3.9556, *p*-value = 0.4121. **(H)** The delta frequency band for Motivation Music and No-Music sessions. The Friedman test did not show a significant difference in PSD across electrodes during Music sessions, χ^2^ = 2.9600, *p*-value = 0.5645. Similarly, there was no significant difference in PSD across electrodes during No-Music sessions, χ^2^ = 6.4800, *p*-value = 0.1661. **(I)** Theta frequency band for Motivation Music and No-Music sessions. The Friedman test did not show a significant difference in PSD across electrodes during Music sessions, χ^2^ = 1.6400, *p* = 0.8016. There was also no significant difference in PSD across electrodes during No-Music sessions, χ^2^ = 7.5200, *p* = 0.1108. **(J)** Alpha frequency band for Motivation Music and No-Music sessions. The Friedman test did not show a significant difference in PSD across electrodes during Music sessions, χ^2^ = 2.4800, *p*-value = 0.6482. Similarly, there was no significant difference in PSD across electrodes during no-music sessions, χ^2^ = 5.0800, *p*-value = 0.2792. **(K)** The beta frequency band for motivation music and no-music sessions. The Friedman test did not show a significant difference in PSD across electrodes during music sessions, χ^2^ = 1.2400, *p*-value = 0.8715. Similarly, there was no significant difference in PSD across electrodes during no-music sessions, χ^2^ = 8.7600, *p*-value = 0.0674. **(L)** Gamma frequency band for motivation music and no-music sessions. The Friedman test did not show a significant difference in PSD across electrodes during Music sessions, χ^2^ = 1.2800, *p*-value = 0.8648. Similarly, there was no significant difference in PSD across electrodes during No-Music sessions, χ^2^ = 7.4400, *p*-value = 0.1144.

We utilized portable devices to record EEG activity while participants engaged in both Focus and Motivation sessions. Topographic maps and Power Spectral Density (PSD) values were used to represent the average power at each frequency band during the baseline recording period, as well as during sections with and without music (Supplementary Figure 4). Additionally, we observed the time-frequency EEG spectrogram distribution of individual participants during the baseline recording period, as well as in sections with and without music. We found notable individual variability in the distribution of power values across frequencies and time (Figures 4A,B). To account for this interindividual variability, we normalized each participant’s average PSD values by their baseline values (Sammler et al., 2007). We then used topographic maps and average PSD values to represent the normalized power at each frequency band (Figure 4). During Focus sessions, we observed a trend of higher power at af4 in the delta (0.5–4 Hz) frequency range and at t7 in the theta (4–8 Hz), beta (13–27 Hz), and gamma (28–40 Hz) frequency ranges (Figures 4A–G). There were no statistically significant differences between mindfulness with music and no music. During Motivation, we observed delta power at frontal af4 and af3 electrodes; however, no statistically significant differences were found between conditions or electrodes (Figures 4H–L).

Given the differences observed in our HRV data when analyzing by reported sex, we also examined potential differences in our EEG data. We found that the increased theta, beta, and gamma power initially observed at the overall group level during Focus sessions was observed in the female group data but absent in the male dataset (Supplementary Figure 5). We also found that during Focus sessions, males exhibited frontal af4 delta power observed during music but not in the no-music condition (Supplementary Figure 5A). The Motivation session also revealed distinctions, as males demonstrated strong posterior alpha power and an additional posterior-temporal beta signal during music mindfulness, which was absent in females (Supplementary Figures 5F,H).

**Figure 5:**
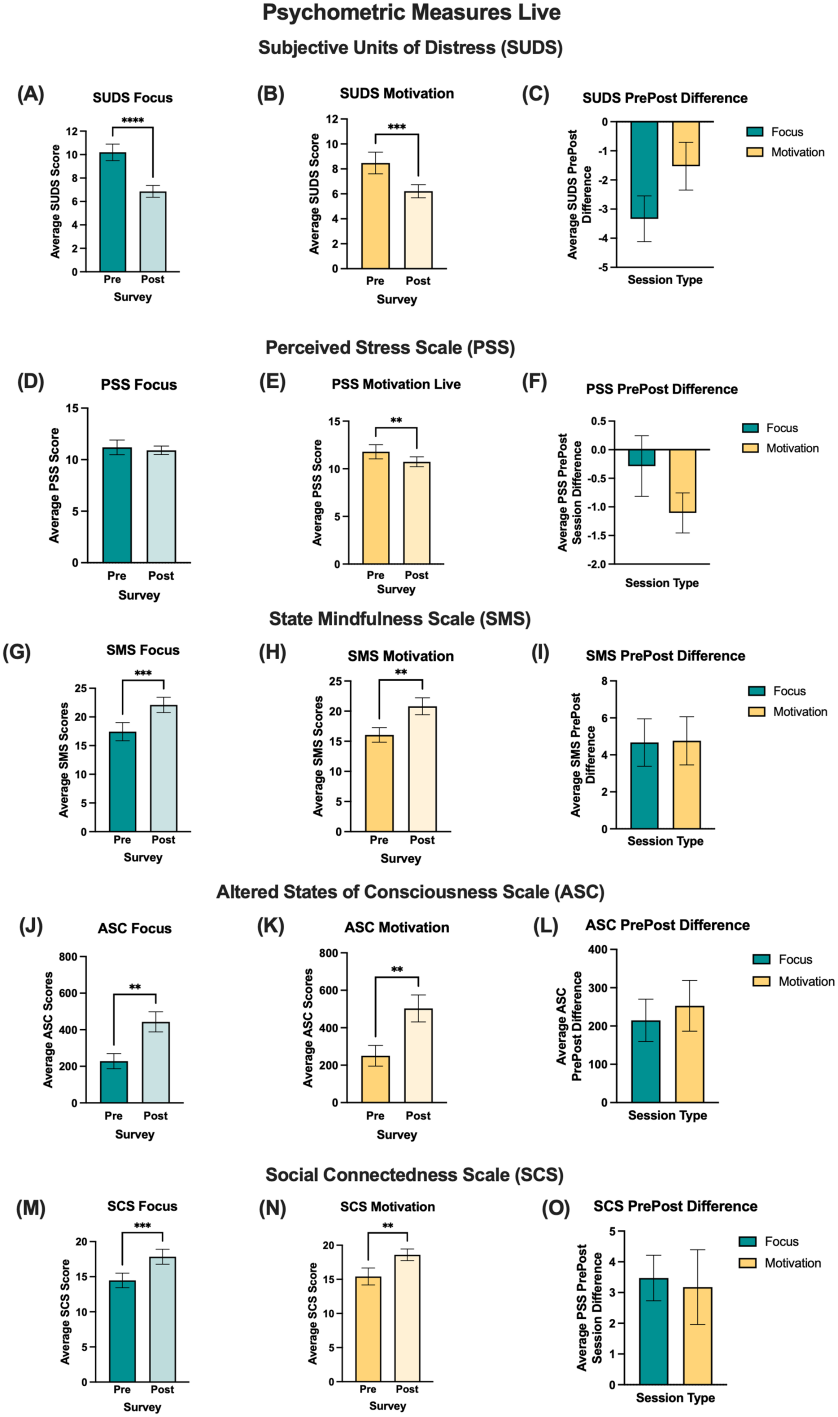
Psychometric data were collected both before and after the focus and motivation sessions, comparing average pre-survey scores with average post-survey scores for all psychometrics across live participants. The first column displays focus meditation, the second column presents motivation sessions, and the last column illustrates the difference between post-survey and pre-survey scores (post-survey minus pre-survey score) for focus and motivation sessions. **(A)** The post score for SUDS was significantly lower than the pre score during Focus meditation. The Wilcoxon matched-pairs signed-rank test showed significance in survey type (W = −192, ****p < 0.0001). **(B)** SUDS post-score was significantly lower than the pre-score during Motivation meditation. The Wilcoxon matched-pairs signed-rank test showed significance in survey type (W = −91, ***p = 0.0002). **(C)** SUDS PrePost Difference Scores between Focus and Motivation. The Mann–Whitney unpaired rank test did not show significance for session type (U = 171.5, *p* = 0.4524). **(D)** The PSS post score was not significantly lower than the pre score during Focus meditation. The paired t-test did not show significance in survey type (t = 0.5384, *p* = 0.5963). **(E)** The PSS post score was significantly lower than the pre score during Motivation meditation. The paired t-test showed significance in survey type (t = 2.874, **p value = 0.0097). **(F)** The PrePost difference scores for focus and motivation meditation were not significant. The unpaired t-test did not indicate significance for session type (t = 1.261, *p* = 0.2149). **(G)** The SMS post score was significantly higher than the pre score during Focus meditation. The Wilcoxon matched-pairs sign rank test showed significance in survey type (W = 167, ***p value = 0.0009). **(H)** The SMS post score was significantly higher than the pre score during the Motivation Meditation. The Wilcoxon matched-pairs signed-rank test showed significance in survey type (W = 158, **p value = 0.0019). **(I)** The SMS PrePost Difference Scores between Focus and Motivation Meditation did not show significance. The Mann–Whitney unpaired rank test also did not show significance in session type (U = 210.5, *p* = 0.8078). **(J)** The ASC post-score was significantly higher than the pre-score during Focus meditation. The paired t-test showed significance in survey type (t = 3.888, **p value = 0.0037). **(K)** The ASC post-score was significantly higher than the pre-score during Motivation meditation. A paired t-test showed significance in survey type (t = 3.811, **p value = 0.0088). **(L)** ASC PrePost difference scores between focus and motivation meditation showed no significance. An unpaired t-test indicated no significance for session type (t = 0.4412, *p* = 0.6654). **(M)** The SCS post-score was significantly higher than the pre-score during Focus meditation. The Wilcoxon matched-pairs sign rank test showed significance in survey type (W = 104, ***p value = 0.0048). **(N)** The SCS post-score was significantly higher than the pre-score during Motivation meditation. The Wilcoxon matched-pairs sign rank test showed significance in survey type (W = 84, **p value = 0.0037). **(O)** The SCS PrePost difference scores between focus and motivation meditation showed no significance. The Mann–Whitney unpaired test also did not reveal significance in session type (U = 147, *p* = 0.6590).

To test whether our music mindfulness sessions led to an improved psychological state in participants, they completed psychometric surveys before and after both the music and non-music sections of the Focus and Motivation sessions. Participants reported significantly reduced stress, as measured by the Subjective Units of Distress assessment (SUDS), after completing both Focus (Wilcoxon matched-pairs signed-rank test, W = −192, ****p < 0.0001) and Motivation (Wilcoxon matched-pairs signed-rank test, W = −91, ***p = 0.0002) sessions (Figures 5A–C). There was no significant difference in Perceived Stress (PSS) after the Focus session (Figure 5D). However, there was a decrease in PSS after the Motivation session (paired t-test, t = 2.874, *p = 0.0097) (Figure 5E), although this difference was not clinically meaningful (Figure 5F). Participants reported increased State Mindfulness (Figures 5G–I) after both Focus (Wilcoxon matched-pairs signed-rank test, W = 167, ***p = 0.0009) and Motivation (Wilcoxon matched-pairs signed-rank test, W = 158, **p = 0.0019) sessions. Similarly, Altered State of Consciousness (ASC) increased during both Focus (paired t-test, t = 3.888, **p = 0.0037) and Motivation (paired t-test, t = 3.811, **p = 0.0088) (Figures 5J–L). Lastly, social connectedness (SCS) was also enhanced after both Focus (Wilcoxon matched-pairs signed-rank test, W = 104, ***p = 0.0048) and Motivation (Wilcoxon matched-pairs signed-rank test, W = 84, **p = 0.0037) sessions (Figures 5M–O).

Given the observed sex differences in HRV and EEG measures, we further assessed the psychometric data based on reported sex. We found that both males (Wilcoxon matched-pairs signed rank test, W = -21, *p = 0.0312) and females (Wilcoxon matched-pairs signed rank test, W = -93, **p = 0.0021) experienced a decrease in SUDS after the Focus session (Supplementary Figures 6A,B). However, only females demonstrated a significant increase in SMS (Wilcoxon matched-pairs signed rank test, W = 77, *p = 0.0125), ASC (paired t-test, t = 4.149, **p = 0.0060), and SCS (paired t-test, t = 4.038, **p = 0.0016) (Supplementary Figures 6J,N,R). Similarly, when we assessed psychometric changes after the Motivation session, we found that females had a decrease in SUDS (Wilcoxon matched-pairs signed rank test, W = -55, **p = 0.0020) and PSS (paired t-test, t = 2.929, *p = 0.0117) (Supplementary Figures 6D,H). We also found an increase in SMS among females (paired t-test, t = 3.195, **p = 0.0065) (Supplementary Figure 6L). These significant changes were not observed in males.

Given the positive effects observed in the psychometric responses of live participants, we assessed whether virtual participants who accessed Focus and Motivation sessions via live stream experienced comparable psychological changes to those observed in the live group. We found that the virtual group exhibited a significant decrease in SUDS during the Focus session (paired t-test, t = 3.173, *p = 0.0238) (Supplementary Figure 7A). Additionally, there was a significant increase in ASC after the Focus session (paired t-test, t = 4.291, *p = 0.0233) (Supplementary Figure 7M). We then directly compared the changes in psychometric scores between live and virtual participants by subtracting pre-survey scores from post-survey scores. We did not identify any significant differences between live and virtual participants in the average psychometric difference scores (Supplementary Figure 7).

**Figure 6:**
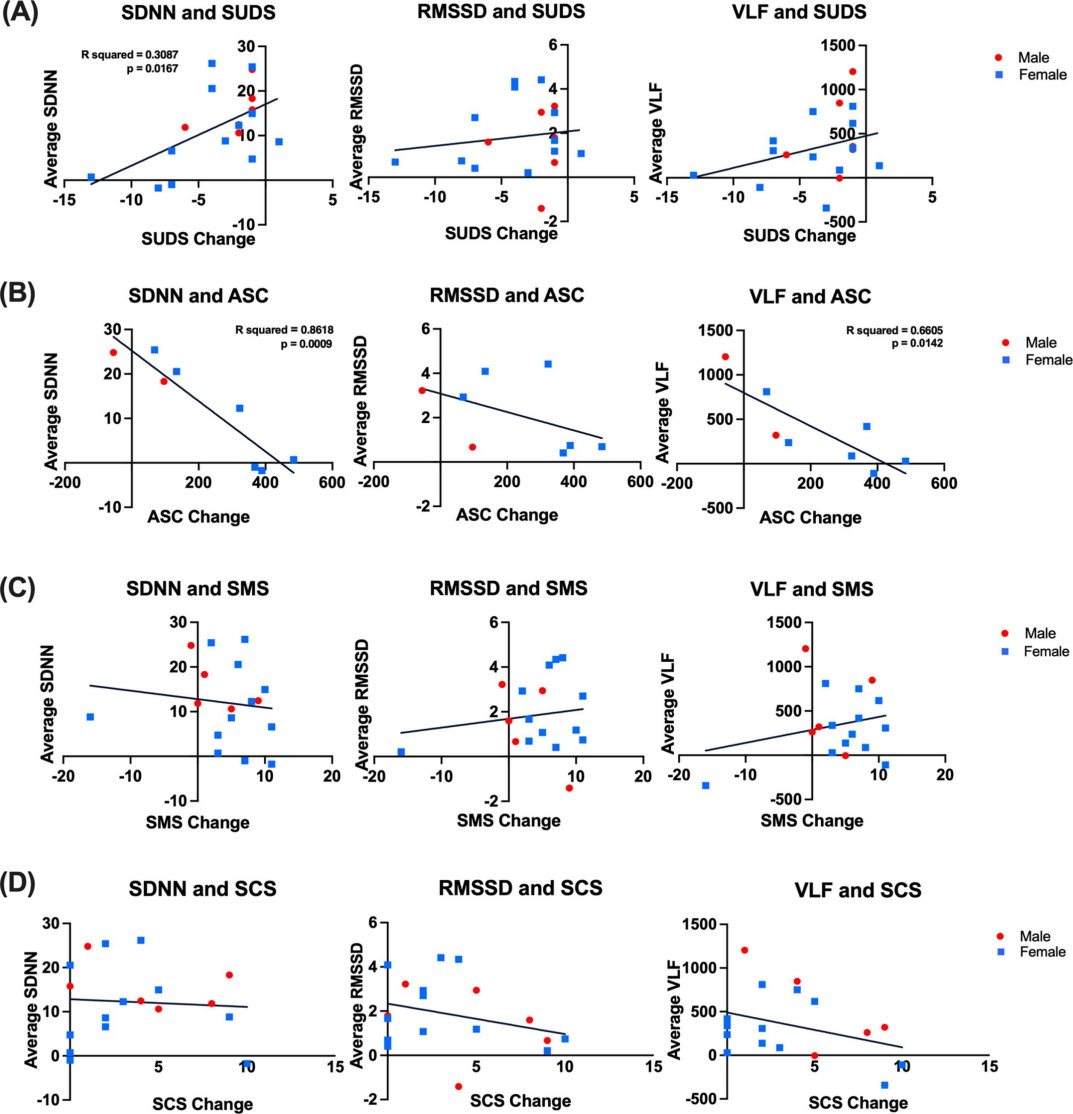
Correlations between changes in HRV metrics and psychometric ratings. **(A)** Left: Correlation between SDNN and SUDS scores during the last 5 min of focus for all participants was significant; a simple linear regression showing the line of best fit with significance (R squared = 0.3087, *p* value = 0.0167). Center: correlation between RMSSD and SUDS scores during the last 5 min of focus for all participants; a simple linear regression showed the line of best fit (R squared = 0.0205, *p* value = 0.5709). Right: Correlation between VLF and SUDS scores during the last 5 min of focus for all participants; a simple linear regression showed the line of best fit (R squared = 0.1095, *p* value = 0.1799). **(B)** Left: Correlation between SDNN and ASC scores during the last 5 min of focus for all participants was significant; a simple linear regression showed the line of best fit with significance (R squared = 0.8618, *p* value = 0.0009). Center: correlation between RMSSD and ASC scores during the last 5 min of focus for all participants; a simple linear regression showed the line of best fit (R squared = 0.2166, *p* value = 0.2452). Right: Correlation between VLF and ASC scores during the last 5 min of focus for all participants; a simple linear regression showed the line of best fit (R squared = 0.6605, *p* value = 0.0142). **(C)** Left: correlation between SDNN and SMS scores during the last 5 min of focus for all participants; a simple linear regression showed the line of best fit (R squared = 0.01868, *p* value = 0.6009). Center: correlation between RMSSD and SMS scores during the last 5 min of focus for all participants; a simple linear regression showed the line of best fit (R squared = 0.02351, *p* value = 0.5568). Right: correlation between VLF and SMS scores during the last 5 min of focus for all participants; a simple linear regression showed the line of best fit (R squared = 0.05658, *p* value = 0.3579). **(D)** Left: correlation between SDNN and SCS scores during the last 5 min of focus for all participants; a simple linear regression showed the line of best fit (R squared = 0.004832, *p*-value = 0.7840). Center: correlation between RMSSD and SCS scores during the last 5 min of focus for all participants; a simple linear regression showed the line of best fit (R squared = 0.08899, *p*-value = 0.2292). Right: correlation between VLF and SCS scores during the last 5 min of focus for all participants; a simple linear regression showed the line of best fit (R squared = 0.1293, *p*-value = 0.1428). SDNN: standard deviation of normal-normal intervals, RMSSD: root mean square of successive interval differences, VLF: very low-frequency power, and LF/HF: ratio of very low-frequency power to very high-frequency power. Error bars denote the standard error of the mean (SEM). SUDS: Subjective Units of Distress; ACS: Altered States of Consciousness Scale; SMS: State Mindfulness Scale; SCS: Social Connectedness Scale.

Finally, we hypothesized that the changes in autonomic system activity observed during Focus sessions could be an underlying mechanism for the psychological benefits of music mindfulness. We examined the three HRV metrics with significant findings during the last 5 min of the Focus sessions—SDNN, RMSSD, and VLF—and subtracted the average baseline value from the average value observed during this period. We then correlated these values with the survey difference scores (post-survey – pre-survey). Surprisingly, we found that a greater change in SDNN from baseline significantly correlated with a reduced change in SUDS (simple linear regression, R squared = 0.3087, *p*-value = 0.0167) (Figure 6A) and ASC (simple linear regression, R squared = 0.8618, *p*-value = 0.0009) (Figure 6B). Additionally, a greater change in VLF from baseline was also significantly correlated with a reduced change in ASC (Figure 6B).

## Discussion

In this study, we demonstrated for the first time that a community-based bilingual music mindfulness session enhances autonomic nervous system activity, modulates neural rhythms, reduces stress, and improves the psychological state of individuals with anxiety and depression. Notably, Focus sessions led to significant increases in SDNN, RMSSD, and VLF after 10 min (Figure 3); however, changes in VLF occurred prior to the changes in RMSSD and SDNN. SDNN reflects a balance of sympathetic and parasympathetic activity, and increased values indicate that music mindfulness enhances autonomic flexibility (Li and Zheng, 2022; Monfredi et al., 2014; Banu and Nagaveni, 2023). RMSSD values reflect parasympathetic activity (Bertsch et al., 2012; DeGiorgio et al., 2010), and our findings provide evidence that Focus music mindfulness sessions enhance parasympathetic activity, while Motivation sessions may not. VLF is associated with various components of the autonomic system, including renin-angiotensin system activity, blood pressure regulation, and thermoregulation, and it has also been associated with markers of inflammation such as CRP and IL-6 (Akselrod et al., 1981; Usui and Nishida, 2017; Janszky et al., 2004; Lampert et al., 2008). We found that music mindfulness enhances VLF as well, and future studies may detail how these varying components—SDNN, RMSSD, and VLF—can be modulated for therapeutic impact. Our study validates the notion that autonomic activity can be rapidly modulated by music mindfulness in individuals with anxiety and depression. Given that decreased HRV metrics may serve as biomarkers for anxiety and depression (Chalmers et al., 2014; Gorman and Sloan, 2000; Sgoifo et al., 2015), the ability of Focus music mindfulness to increase both parasympathetic and sympathetic HRV activity makes it a promising mechanism-based MBI. In this study, we only used HRV metrics as an index of autonomic activity. Using higher-density ECG recordings and adding measurements for blood pressure, respiration, and skin conductance will provide a more detailed physiological understanding to inform therapeutic models.

Surprisingly, our correlation analyses suggest that reduced HRV reactivity (measured by smaller changes in SDNN from baseline) is associated with greater reductions in distress and greater alterations of consciousness during Focus sessions (Figure 6). Phasic HRV suppression has previously been linked to attentional processes, stress regulation, and executive functioning (Pham et al., 2021; Laborde et al., 2017; Thayer et al., 1996). Therefore, the correlation we found indicates that the impact of music mindfulness on autonomic reactivity during focused attention may be relevant to its ability to reduce distress and alter consciousness.

Our EEG qualitative analysis revealed interindividual variability in power across frequency and time. Normalizing power by the baseline condition during sections with and without music revealed a trend for higher t7 power in theta, beta, and gamma frequency bands (Figure 4) during Focus sessions, which was not observed during Motivation sessions. Interestingly, increased left temporal theta has been observed after music therapy and is associated with emotional music performance (Fachner et al., 2013; Ghodousi et al., 2022). In non-musicians, left temporal gamma is seen when individuals are at rest with their eyes closed and listening to music (Bhattacharya et al., 2001). Left temporal beta power is a reliable EEG marker for linguistic processing and may reflect the processing of mindfulness instructions across the session (Spironelli and Angrilli, 2010). While delta oscillations are primarily associated with sleep, they have also been believed to play a role in motivational cognitive processes related to attention (Knyazev, 2012), and increased delta power is believed to inhibit sensory afference to facilitate internal concentration (Harmony, 2013). One outcome of our naturalistic approach is that we are limited to portable headsets with a relatively small number of electrodes, leading to sparse signaling. This limitation constrains our ability to analyze this EEG data further using methods such as source localization. The fidelity of the EEG signal as a potential biomarker would improve in a laboratory setting using higher-density EEG electrodes.

Interestingly, our study suggests that participants’ physiological and psychological responses to music mindfulness differ based on their reported sex. We find that females demonstrate significant increases in SDNN and RMSSD during music mindfulness, which are not observed in males (Supplementary Figure 4). While our small sample size limits our study’s power to assess sex differences, previous research has shown variations in HRV values, indicating less power in HRV power spectral density for females than for men (Koenig and Thayer, 2016; Geovanini et al., 2020; Liao et al., 1995). Additionally, differences in HRV responses to mental stress have been observed (Adjei et al., 2018).

Differences between sexes have also been observed in EEG signals, with females exhibiting either greater or lesser power in the delta, theta, alpha, and beta frequency bands depending on the task (Hashemi et al., 2016; Cave and Barry, 2021; Brenner et al., 1995; Latta et al., 2005). Females also show greater right-hemisphere activation during emotional processing and better control of asymmetrical cortical patterning (Davidson et al., 1976). Evidence suggests there are differences in music processing, with a tendency for more bilateral processing in females (Koelsch et al., 2003). The differences in EEG data between males and females may relate to differences in brain anatomy, such as thicker cortical gray matter in females, or (Sowell et al., 2007) may stem from differences in neural processes (Rabinowicz et al., 1999). However, when analyzing these observed and reported differences, we do not posit a genetic or biological origin, as sociocultural influences and gender socialization may also affect the observed differences in HRV, EEG activity, and psychometrics (Unger, 1979; Marini, 1990). Previous research indicates that women have a higher prevalence of mood disorders such as anxiety and depression. However, the role of sociocultural differences, sampling biases, and biases in diagnostic criteria should be considered when interpreting these findings (Hartung and Widiger, 1998; Klose and Jacobi, 2004; Rich-Edwards et al., 2018). Music mindfulness may provide a particularly effective therapeutic approach for females, given its potentially unique impact on autonomic activity. This method could represent a promising opportunity for the non-pharmacological management of anxiety and depression during the peripartum and perimenopausal periods (Qi et al., 2023; Evans et al., 2018; Domínguez-Solís et al., 2021).

We found that Focus sessions modulated autonomic activity, EEG signatures, and psychometrics differently than Motivation sessions. The primary differences between Focus and Motivation sessions lie in their supporting music features and the nature of the guided mindfulness activities practiced during each session. The music for the Focus session was in the key of B Major and had a tempo of 72 BPM. It was composed of a number of tracks, timbre choices, and other features guided by principles of music designed to support mindfulness (Dvorak and Hernandez-Ruiz, 2021). In contrast, the music for the Motivation session was in the key of E minor and had a tempo of 164 BPM. Motivational music exhibits greater rhythmic flexibility and a higher tempo to enhance arousal and reduce parasympathetic measures (Watanabe et al., 2017). Research indicates that music with slower tempos has been associated with larger increases in parasympathetic activity, while faster tempos have been associated with arousal (Bretherton et al., 2019; Koelsch and Jäncke, 2015; Husain et al., 2002; Carpentier et al., 2007; Kim et al., 2019). Moreover, tempo has been shown to impact the valence of music (Ooishi et al., 2017). Thus, the differences in music composition features (tempo, key, mode) between Focus and Motivation sessions may explain the physiological differences observed in HRV.

Modern models of mindfulness categorize mindfulness practices into focused attention and open monitoring activities, with different neural networks proposed to support them (Vago and David, 2012; Fujino et al., 2018). In addition, various cognitive dimensions differentiate the different mindfulness practices (Matko and Sedlmeier, 2019; Burke, 2012). In this study, we adopted this broad framework of focused attention and open monitoring while incorporating visualization and cognitive restructuring into open monitoring during the Motivation sessions. Focused attention activates various cortical networks and relies on regions such as the dorsolateral prefrontal cortex (dlPFC) (Manna et al., 2010). Open monitoring is believed not to rely on the dlPFC but rather on regions such as the anterior cingulate cortex (ACC) and the ventrolateral prefrontal cortex (vlPFC) (Lippelt et al., 2014; Manna et al., 2010). Activating these different nodes and their corresponding networks may also account for the differences in EEG observed between Focus and Motivation sessions. These neural variations might also impact HRV signals, as cortical structures like the ACC are linked to the regulation of autonomic activity (Seamans and Floresco, 2022; Critchley et al., 2003, 2005).

Despite the differences in music composition, mindfulness activities, and the underlying differences in HRV and neural network activation, both Focus and Motivation sessions are helpful in relieving stress and modulating psychological states. For example, both Motivation and Focus lead to decreased distress (SUDs) as well as enhanced mindfulness (SMS), altered states of consciousness (ASC), and social connection (SCS). Overall, these findings suggest that while HRV changes may be significant for Focus sessions, they do not explain the stress-reduction effects of Motivation-based interventions.

Notably, we were able to compare the psychological impact of both live and virtual music mindfulness sessions. During the virtual session, there was a significant decrease in the SUDS score during the Focus sessions but not in the Motivation sessions (Supplementary Figure 7). Additionally, there was an increase in ASC during the Focus sessions but not during the Motivation session. For the virtual group, no other significant differences were observed between the Focus and Motivation sessions. This contrasts with the live sessions, where SUDS decreased, SMS increased for both Focus and Motivation, while PSS decreased for Motivation (Figure 5). For both live and virtual sessions, ASC improved during Focus sessions. Altered states of consciousness have long been viewed as having therapeutic value in community and clinical settings (Ludwig, 1966; Sheppard, 2024). The therapeutic value of altered states may be associated with changes in autonomic system activity and frontotemporal networks (Dietrich, 2003; Boly et al., 2008; Maldonato et al., 2018; Aftanas and Golocheikine, 2001; Oswald et al., 2023). This data provides evidence for the induction of an altered state of consciousness through music mindfulness as a potential therapeutic psychological mechanism. Future work will utilize controlled experiments to detail the autonomic and central nervous system mechanisms that modulate psychological states.

Interestingly, we also found that social connectedness (SCS) increased during the live Focus and Motivation sessions, while SCS did not increase during the virtual session. This is consistent with the idea that in-person settings enhance social connections in ways that may not be replicated on virtual platforms. Furthermore, our previous pilot study of a virtual music mindfulness platform found no increase or correlation with social connectedness; however, we did observe a correlation between time spent in music mindfulness and decreased stress (Igbinobaro et al., 2024). Lastly, live music has been shown to engage listeners, alleviate symptoms, and improve relationships more than recorded music (Swarbrick et al., 2019; Bailey, 1983; Clare and Camic, 2020), which may directly mediate some of the differences observed. In our study, the live audio and video were professionally mixed and streamed directly, and virtual participants were instructed to use headphones for a more immersive experience. They were also encouraged to participate in a relaxing room or comfortable space. However, technical issues such as audio-visual signal lag, low-quality headphones, and lack of environmental control may have all had an effect on the experience of virtual participants.

Overall, when directly comparing the difference scores for psychometrics between live and virtual sessions, we do not find a significant difference. Previous studies have shown that virtual and online therapeutic approaches are effective for the management of symptoms like anxiety and depression (Reangsing et al., 2023; Fernandez et al., 2021; Saddichha et al., 2014; Roy et al., 2021). Thus, the reduction in distress and increase in altered states of consciousness observed in our virtual group further supports the implementation of virtual approaches for treating symptoms such as anxiety. One caveat when interpreting this data is that we have a relatively low number of virtual participants, resulting in decreased power to detect statistically significant effects within the virtual group. Our results imply that while virtual sessions reduce stress, they do not have as extensive an effect on psychological states as in-person sessions do. Our results also suggest that important aspects of live social interaction may drive feelings of social connection and serve as a key differentiator between live and virtual sessions.

Clinical applications of music mindfulness approaches for managing symptoms of anxiety, depression, and pain already demonstrate therapeutic promise (Knoerl et al., 2022; Liu et al., 2019; Hwang et al., 2023; Chan et al., 2023; Qi et al., 2023; Soo et al., 2016). There is also growing evidence that cognitive strategies enhancing focused attention may have a therapeutic impact on multiple psychiatric conditions (Keller et al., 2019; Rubia, 2009). Our Focus music mindfulness sessions may alleviate distress across multiple diagnoses and directly improve focused attention in a sustained manner.

Music mindfulness can also play an important role in psychedelic-assisted therapy, which has re-emerged as a promising therapeutic modality for stress-related disorders such as PTSD, anxiety, and depression (Mitchell et al., 2023; Carhart-Harris et al., 2021; Gukasyan et al., 2022; Griffiths et al., 2016; Grob et al., 2011). Music is already an important facilitative tool during psychedelic dosing sessions (Adamska and Finc, 2023; Kaelen et al., 2015; Kaelen et al., 2018; Barrett et al., 2017; Bonny and Pahnke, 1972) and can support meaning-making, emotionality, and mental imagery after psychedelic administration (Barrett et al., 2017; Kaelen et al., 2018; Barrett et al., 2018). Mindfulness has been shown to modify participants’ responses to psychedelics, while psychedelics can enhance mindfulness (Radakovic et al., 2022; Smigielski et al., 2019; Meling et al., 2024; Chambers et al., 2023; Eleftheriou and Thomas, 2021). For example, psilocybin can enhance meditation depth and incidence of positively experienced self-dissolution without anxiety (Smigielski et al., 2019). Music mindfulness interventions may be particularly impactful as part of preparation, dosing, and integration sessions for psychedelic-assisted therapy.

In this study, we implemented a community-based approach to enroll participants and collect data in a naturalistic setting. Our approach can be readily adapted to other community spaces with existing trust, such as schools, community centers, and churches (Jordan et al., 2021). Individuals identified by community providers as having moderate to severe anxiety or depression would participate in music mindfulness group sessions daily or weekly at a local community center or wellness center. Musicians in this model can be professional musicians sourced from local orchestras, bands, or music schools and do not require specific music therapy training. Facilitators can be local mindfulness and meditation teachers; however, social workers, psychologists, psychiatrists, and nurses can also read mindfulness scripts and create a supportive environment for the music mindfulness sessions if they have prior teacher or facilitator training. These sessions could also be conducted in mental health centers or outpatient clinics for patients who have progressed to moderate or severe anxiety but do not yet need hospitalization. They can participate in these sessions daily or weekly to regulate autonomic activity, reduce distress, and prevent hospitalization. Other potential uses for community-based music mindfulness include individuals with anxiety or agitation living in group or residential facilities. Daily or weekly sessions at the residential facility could help manage symptoms and reduce acute distress. Music mindfulness interventions may also provide a non-pharmacological approach for treating anxiety or agitation in individuals with dementia, where polypharmacy can present an additional challenge for symptom management (Dimitriou et al., 2020; Cooke et al., 2010; Ing-Randolph et al., 2015). Finally, music therapists already use instruments such as the monochord to facilitate mindfulness activities (Gäbel et al., 2017; Lee and Bhattacharya, 2013; Lee et al., 2012). Music therapists could also use music mindfulness backing tracks and guided activities to work with patients and lead them through these scripted guided exercises or improvisations as part of their music therapy sessions.

In this study, we provide the first physiological evidence that targeting autonomic activity is a transdiagnostic mechanistic framework for music mindfulness interventions. Future studies will assess the impact of 8-week music mindfulness paradigms on managing symptoms of anxiety and depression.

While these initial data provide first-of-its-kind analyses, there are limitations to our naturalistic approach. While we recruit participants directly from the local community through convenience sampling, this method introduces selection bias in our results and limits the generalizability of our study. The naturalistic setting also results in a less controlled environment than a laboratory-based study, which may introduce noise into our data. We also have a sample size with limited power to perform correlational and other secondary analyses. In this study, we utilized mobile technology to record EEG signals in a naturalistic setting. However, we use low-density headsets, which are more susceptible to artifacts and lack spatial specificity. High-density EEG recordings in a larger sample will better characterize EEG signatures in females and males during music mindfulness. Finally, to encourage community participation and completion of survey data, we shortened multiple psychometric scales. We demonstrate that these shortened versions of the scales have internal reliability for the measured constructs. However, when factor analysis is conducted, they are unidimensional and cannot capture the various factors that may impact our measured psychological constructs. These shortened scales will require further validation through larger studies in a randomized controlled trial.

Our study leverages community partnerships and wearable technology (Alhejaili and Alomainy, 2023; Gomes et al., 2023; Theofanopoulou et al., 2024) to detail the physiological and psychological impacts of music mindfulness for individuals with anxiety and depression in real-world settings. This approach aligns with the efforts to balance the empirical rigor of clinical trials with ecological validity (Habibi et al., 2022). We find that community-based music mindfulness enhances both sympathetic and parasympathetic system activity within 10-min, reduces distress, and alters states of consciousness in individuals with mood disorders. These acute changes in an autonomic state may be central to music mindfulness’s ability to relieve stress in individuals with anxiety and depression.

## Data Availability

The datasets presented in this study can be found in online repositories. The names of the repository/repositories and accession number(s) can be found at: https://osf.io/4h7wm/?view_only=09ff4a216c68436e862d03a5b8632267.

## Footnotes

^1^ https://aura-healthcare.github.io/hrv-analysis/

## References

[ref1] Aalbers Sonja Fusar-Poli Laura Freeman Ruth E. Spreen Marinus Ket Johannes CF Vink Annemiek C.. (2017) “Music Therapy for Depression, Aalbers S. Cochrane Library.” Accessed December 8, 2024. Available online at: https://www.cochranelibrary.com/cdsr/doi/10.1002/14651858.CD004517.pub3/full 10.1002/14651858.CD004517.pub3 PMC6486188 29144545

[ref2] Adamska I. Finc K. (2023). Effect of LSD and music on the time-varying brain dynamics. Psychopharmacology 240, 1601–1614. doi: 10.1007/s00213-023-06394-8, PMID: 37291360 PMC10272181

[ref3] Adjei T. Xue J. Mandic D. P. (2018). The female heart: sex differences in the dynamics of ECG in response to stress. Front. Physiol. 9:1616. doi: 10.3389/fphys.2018.01616, PMID: 30546313 PMC6279887

[ref4] Aftanas L. I. Golocheikine S. A. (2001). Human anterior and frontal midline Theta and lower alpha reflect emotionally positive state and internalized attention: high-resolution EEG investigation of meditation. Neurosci. Lett. 310, 57–60. doi: 10.1016/s0304-3940(01)02094-8, PMID: 11524157

[ref5] Akselrod S. David Gordon F. Ubel A. Shannon D. C. Clifford Berger A. Cohen R. J. (1981). Power Spectrum analysis of heart rate fluctuation: a quantitative probe of beat-to-beat cardiovascular control. Science 213, 220–222. doi: 10.1126/science.6166045, PMID: 6166045

[ref6] Alhejaili R. Alomainy A. (2023). The use of wearable Technology in Providing Assistive Solutions for mental well-being. Sensors (Basel, Switzerland) 23:7378. doi: 10.3390/s23177378, PMID: 37687834 PMC10490605

[ref7] Alqatari S. Kelly L. Fitzpatrick K. Cheung P. S. Moss H. (2022). Mindful music—a pilot study of the effects of mindfulness-based music on staff members of the University of Limerick. Music Med 14, 115–124. doi: 10.47513/mmd.v14i2.853

[ref8] Asif A. Majid M. Anwar S. M. (2019). Human stress classification using EEG signals in response to music tracks. Comput. Biol. Med. 107, 182–196. doi: 10.1016/j.compbiomed.2019.02.015, PMID: 30836290

[ref9] Bachman N. Palgi Y. Bodner E. (2022). Emotion regulation through music and mindfulness are associated with positive solitude differently at the second half of life. Int. J. Behav. Dev. 46, 520–527. doi: 10.1177/01650254221131304

[ref10] Baik S. H. Fox R. S. Mills S. D. Roesch S. C. Sadler G. R. Klonoff E. A.. (2019). Reliability and validity of the perceived stress Scale-10 in Hispanic Americans with English or Spanish language preference. J. Health Psychol. 24, 628–639. doi: 10.1177/1359105316684938, PMID: 28810432 PMC6261792

[ref11] Bailey L. M. (1983). The effects of live music versus tape-recorded music on hospitalized Cancer patients. Music. Ther. 3, 17–28. doi: 10.1093/mt/3.1.17

[ref12] Banu A. R. S. Nagaveni V. (2023). Assessment of sympathetic and parasympathetic activities of nervous system from heart rate variability using machine learning techniques. SN Comput. Sci. 4:646. doi: 10.1007/s42979-023-02062-y

[ref13] Barrett F. S. Preller K. H. Kaelen M. (2018). Psychedelics and music: neuroscience and therapeutic implications. Int. Rev. Psychiatry 30, 350–362. doi: 10.1080/09540261.2018.1484342, PMID: 30240282

[ref14] Barrett F. S. Robbins H. Smooke D. Brown J. L. Griffiths R. R. (2017). Qualitative and quantitative features of music reported to support peak mystical experiences during psychedelic therapy sessions. Front. Psychol. 8:1238. doi: 10.3389/fpsyg.2017.01238, PMID: 28790944 PMC5524670

[ref15] Basso J. C. McHale A. Ende V. Oberlin D. J. Suzuki W. A. (2019). Brief, daily meditation enhances attention, memory, mood, and emotional regulation in non-experienced meditators. Behav. Brain Res. 356, 208–220. doi: 10.1016/j.bbr.2018.08.023, PMID: 30153464

[ref16] Baylan S. McGinlay M. MacDonald M. Easto J. Cullen B. Haig C.. (2018). Participants’ experiences of music, mindful music, and audiobook listening interventions for people recovering from stroke. Ann. New York Acad. Sci. 1423, 349–359. doi: 10.1111/nyas.13618, PMID: 29727009

[ref17] Benjamin C. L. O’Neil K. A. Crawley S. A. Beidas R. S. Coles M. Kendall P. C. (2010). Patterns and predictors of subjective units of distress in anxious youth. Behav. Cogn. Psychother. 38, 497–504. doi: 10.1017/S1352465810000287, PMID: 20509987 PMC4874244

[ref18] Berntson G. G. Cacioppo J. T. (2004). “Heart rate variability: stress and psychiatric conditions” in Dynamic electrocardiography. Eds. M. Malik and A. J. Camm (Hoboken, NJ: John Wiley & Sons, Ltd.), 57–64.

[ref19] Bertsch K. Hagemann D. Naumann E. Schächinger H. Schulz A. (2012). Stability of heart rate variability indices reflecting parasympathetic activity. Psychophysiology 49, 672–682. doi: 10.1111/j.1469-8986.2011.01341.x, PMID: 22335779

[ref20] Bhattacharya J. Petsche H. Pereda E. (2001). Long-range synchrony in the γ band: role in music perception. J. Neurosci. 21, 6329–6337. doi: 10.1523/JNEUROSCI.21-16-06329.2001, PMID: 11487656 PMC6763152

[ref21] Blood A. J. Zatorre R. J. (2001). Intensely pleasurable responses to music correlate with activity in brain regions implicated in reward and emotion. Proc. Natl. Acad. Sci. 98, 11818–11823. doi: 10.1073/pnas.191355898, PMID: 11573015 PMC58814

[ref22] Boly M. Phillips C. Tshibanda L. Vanhaudenhuyse A. Schabus M. Dang-Vu T. T.. (2008). Intrinsic brain activity in altered states of consciousness. Ann. N. Y. Acad. Sci. 1129, 119–129. doi: 10.1196/annals.1417.015, PMID: 18591474 PMC2855379

[ref23] Bonny H. L. Pahnke W. N. (1972). The use of music in psychedelic (LSD) psychotherapy. J. Music. Ther. 9, 64–87. doi: 10.1093/jmt/9.2.64

[ref24] Braboszcz C. Rael Cahn B. Levy J. Fernandez M. Delorme A. (2017). Increased Gamma brainwave amplitude compared to control in three different meditation traditions. PLoS One 12:e0170647. doi: 10.1371/journal.pone.0170647, PMID: 28118405 PMC5261734

[ref25] Bradt Joke Dileo Cheryl Shim Minjung. (2013) “Music Interventions for Preoperative Anxiety. Bradt J Cochrane Library.” Accessed December 8, 2024. Available online at: https://www.cochranelibrary.com/cdsr/doi/10.1002/14651858.CD006908.pub2/full. 10.1002/14651858.CD006908.pub2 PMC9758540 23740695

[ref26] Brenner R. P. Ulrich R. F. Reynolds C. F. (1995). EEG spectral findings in healthy, elderly men and women — sex differences. Electroencephalogr. Clin. Neurophysiol. 94, 1–5. doi: 10.1016/0013-4694(94)00234-C 7530634

[ref27] Bretherton B. Deuchars J. Luke Windsor W. (2019). The effects of controlled tempo manipulations on cardiovascular autonomic function. Music Sci. 2:2059204319858281. doi: 10.1177/2059204319858281, PMID: 40066071

[ref28] Burke A. (2012). Comparing individual preferences for four meditation techniques: Zen, Vipassana (mindfulness), qigong, and mantra. Explore 8, 237–242. doi: 10.1016/j.explore.2012.04.003, PMID: 22742674

[ref29] Cahn B. R. Delorme A. Polich J. (2013). Event-Related Delta, Theta, alpha and Gamma correlates to auditory oddball processing during Vipassana meditation. Soc. Cogn. Affect. Neurosci. 8, 100–111. doi: 10.1093/scan/nss060, PMID: 22648958 PMC3541491

[ref30] Carhart-Harris R. Giribaldi B. Watts R. Baker-Jones M. Murphy-Beiner A. Murphy R.. (2021). Trial of psilocybin versus Escitalopram for depression. N. Engl. J. Med. 384, 1402–1411. doi: 10.1056/NEJMoa2032994, PMID: 33852780

[ref31] Carpentier D. Francesca R. Potter R. F. (2007). Effects of music on physiological arousal: explorations into tempo and genre. Media Psychol. 10, 339–363. doi: 10.1080/15213260701533045

[ref32] Cave A. E. Barry R. J. (2021). Sex differences in resting EEG in healthy Young adults. Int. J. Psychophysiol. 161, 35–43. doi: 10.1016/j.ijpsycho.2021.01.008, PMID: 33454318

[ref33] Chalmers J. A. Quintana D. S. Anne Abbott M. J. Kemp A. H. (2014). Anxiety disorders are associated with reduced heart rate variability: a Meta-analysis. Front. Psych. 5. doi: 10.3389/fpsyt.2014.00080, PMID: 25071612 PMC4092363

[ref34] Chambers R. Stoliker D. Simonsson O. (2023). Psychedelic-assisted psychotherapy and mindfulness-based cognitive therapy: potential synergies. Mindfulness 14, 2111–2123. doi: 10.1007/s12671-023-02206-4

[ref35] Chan S. H. W. Cheung M. Y. C. Chiu A. T. S. Leung M. H. T. Kuo M. C. C. Yip D. Y. C.. (2023). Clinical effectiveness of mindfulness-based music therapy on improving emotional regulation in blind older women: a randomized controlled trial. Integ. Med. Res. 12:100993. doi: 10.1016/j.imr.2023.100993, PMID: 37915438 PMC10616413

[ref36] Cheever T. Taylor A. Finkelstein R. Edwards E. Thomas L. Bradt J.. (2018). NIH/Kennedy center workshop on music and the brain: finding Harmony. Neuron 97, 1214–1218. doi: 10.1016/j.neuron.2018.02.004, PMID: 29566791 PMC6688399

[ref37] Chen W. G. Edwards E. Iyengar S. Finkelstein R. Rutter D. F. Fleming R.. (2024). Music and medicine: quickening the tempo of Progress. Lancet 403, 1213–1215. doi: 10.1016/S0140-6736(24)00477-X, PMID: 38513679

[ref38] Chen W. G. Iversen J. R. Kao M. H. Loui P. Patel A. D. Zatorre R. J.. (2022). Music and brain circuitry: strategies for strengthening evidence-based research for music-based interventions. J. Neurosci. 42, 8498–8507. doi: 10.1523/JNEUROSCI.1135-22.2022, PMID: 36351825 PMC9665917

[ref39] Cheung V. K. M. Harrison P. M. C. Meyer L. Pearce M. T. Haynes J.-D. Koelsch S. (2019). Uncertainty and surprise jointly predict musical pleasure and amygdala, Hippocampus, and auditory cortex activity. Curr. Biol. 29, 4084–4092.e4. doi: 10.1016/j.cub.2019.09.067 31708393

[ref40] Chiesa A. Serretti A. (2010). A systematic review of neurobiological and clinical features of mindfulness meditations. Psychol. Med. 40, 1239–1252. doi: 10.1017/S0033291709991747, PMID: 19941676

[ref41] Christodoulou G. Salami N. Black D. S. (2020). The utility of heart rate variability in mindfulness research. Mindfulness 11, 554–570. doi: 10.1007/s12671-019-01296-3

[ref42] Chu H. Chyn-Yng Yang Y. Lin K.-L. O. Lee T.-Y. O’Brien A. P. Chou K.-R. (2014). The impact of group music therapy on depression and cognition in elderly persons with dementia: a randomized controlled study. Biol. Res. Nurs. 16, 209–217. doi: 10.1177/1099800413485410, PMID: 23639952

[ref43] Clare A. Camic P. M. (2020). Live and recorded group music interventions with active participation for people with dementias: a systematic review. Arts Health 12, 197–220. doi: 10.1080/17533015.2019.1675732, PMID: 31583964

[ref44] Cohen S. (1988). “Perceived stress in a probability sample of the United States” in The social psychology of health, 31–67. The Claremont symposium on applied social psychology (Thousand Oaks, CA, US: Sage Publications, Inc.).

[ref45] Collins F. S. Fleming R. (2017). Sound health: an NIH-Kennedy center initiative to explore music and the mind. JAMA 317, 2470–2471. doi: 10.1001/jama.2017.7423, PMID: 28586832 PMC6688192

[ref46] Cooke M. L. Moyle W. Shum D. H. K. Harrison S. D. Murfield J. E. (2010). A randomized controlled trial exploring the effect of music on agitated Behaviours and anxiety in older people with dementia. Aging Ment. Health 14, 905–916. doi: 10.1080/13607861003713190, PMID: 20635236

[ref47] Critchley H. D. Mathias C. J. Josephs O. O’Doherty J. Zanini S. Dewar B.-K.. (2003). Human cingulate cortex and autonomic control: converging neuroimaging and clinical evidence. Brain 126, 2139–2152. doi: 10.1093/brain/awg216, PMID: 12821513

[ref48] Critchley H. D. Tang J. Glaser D. Butterworth B. Dolan R. J. (2005). Anterior cingulate activity during error and autonomic response. NeuroImage 27, 885–895. doi: 10.1016/j.neuroimage.2005.05.047, PMID: 15996878

[ref49] Daly I. Malik A. Hwang F. Roesch E. Weaver J. Kirke A.. (2014). Neural correlates of emotional responses to music: an EEG study. Neurosci. Lett. 573, 52–57. doi: 10.1016/j.neulet.2014.05.003, PMID: 24820541

[ref50] Davidson R. J. Schwartz G. E. Pugash E. Bromfield E. (1976). Sex differences in patterns of EEG asymmetry. Biol. Psychol. 4, 119–137. doi: 10.1016/0301-0511(76)90012-0 1276303

[ref51] DeGiorgio C. M. Miller P. Meymandi S. Chin A. Epps J. Gordon S.. (2010). RMSSD, a measure of Vagus-mediated heart rate variability, is associated with risk factors for SUDEP: the SUDEP-7 inventory. Epilepsy Behav. 19, 78–81. doi: 10.1016/j.yebeh.2010.06.011, PMID: 20667792 PMC2943000

[ref52] Diaz F. M. (2013). Mindfulness, attention, and flow during music listening: an empirical investigation. Psychol. Music 41, 42–58. doi: 10.1177/0305735611415144

[ref53] Dietrich A. (2003). Functional neuroanatomy of altered states of consciousness: the transient Hypofrontality hypothesis. Conscious. Cogn. 12, 231–256. doi: 10.1016/S1053-8100(02)00046-6, PMID: 12763007

[ref54] Dimitriou T.-D. Verykouki E. Papatriantafyllou J. Konsta A. Kazis D. Tsolaki M. (2020). Non-pharmacological interventions for the anxiety in patients with dementia. A cross-over randomised controlled trial. Behav. Brain Res. 390:112617. doi: 10.1016/j.bbr.2020.112617, PMID: 32428636

[ref55] Dittrich A. (2007). The standardized psychometric assessment of altered states of consciousness (ASCs) in humans. Pharmacopsychiatry 31, 80–84. doi: 10.1055/s-2007-979351, PMID: 9754838

[ref56] Domínguez-Solís E. Lima-Serrano M. Lima-Rodríguez J. S. (2021). Non-pharmacological interventions to reduce anxiety in pregnancy, labour and postpartum: a systematic review. Midwifery 102:103126. doi: 10.1016/j.midw.2021.103126, PMID: 34464836

[ref57] Dvorak A. L. Hernandez-Ruiz E. (2021). Comparison of music stimuli to support mindfulness meditation. Psychol. Music 49, 498–512. doi: 10.1177/0305735619878497

[ref58] Eckhardt K. J. Dinsmore J. A. (2012). Mindful music listening as a potential treatment for depression. J. Creat. Ment. Health 7, 175–186. doi: 10.1080/15401383.2012.685020

[ref59] Eleftheriou M. E. Thomas E. (2021). Examining the potential synergistic effects between mindfulness training and psychedelic-assisted therapy. Front. Psych. 12. doi: 10.3389/fpsyt.2021.707057, PMID: 34456763 PMC8386240

[ref60] Ellis Robert J. Thayer Julian F. (2010) “Music and Autonomic Nervous System (Dys)Function.” Accessed December 28, 2024. Available online at: https://online.ucpress.edu/mp/article/27/4/317/62457/Music-and-Autonomic-Nervous-System-Dys-Function. 10.1525/mp.2010.27.4.317 PMC3011183 21197136

[ref61] Evans K. Jane Morrell C. Spiby H. (2018). Systematic review and Meta-analysis of non-pharmacological interventions to reduce the symptoms of mild to moderate anxiety in pregnant women. J. Adv. Nurs. 74, 289–309. doi: 10.1111/jan.13456, PMID: 28921612

[ref62] Faber S. Belden A. Loui P. McIntosh A. R. (2024). Network connectivity differences in music listening among older adults following a music-based intervention. Aging Brain 6:100128. doi: 10.1016/j.nbas.2024.100128, PMID: 39539646 PMC11558634

[ref63] Fachner J. Gold C. Erkkilä J. (2013). Music therapy modulates Fronto-temporal activity in rest-EEG in depressed clients. Brain Topogr. 26, 338–354. doi: 10.1007/s10548-012-0254-x, PMID: 22983820

[ref64] Fancourt D. Williamon A. Carvalho L. A. Steptoe A. Dow R. Lewis I. (2016). Singing modulates mood, stress, cortisol, cytokine and neuropeptide activity in Cancer patients and Careers. Ecancermedicalscience 10:631. doi: 10.3332/ecancer.2016.631, PMID: 27170831 PMC4854222

[ref65] Fell J. Axmacher N. Haupt S. (2010). From alpha to Gamma: electrophysiological correlates of meditation-related states of consciousness. Med. Hypotheses 75, 218–224. doi: 10.1016/j.mehy.2010.02.025, PMID: 20227193

[ref66] Fernandez E. Woldgabreal Y. Day A. Pham T. Gleich B. Aboujaoude E. (2021). Live psychotherapy by video versus in-person: a Meta-analysis of efficacy and its relationship to types and targets of treatment. Clin. Psychol. Psychother. 28, 1535–1549. doi: 10.1002/cpp.2594, PMID: 33826190

[ref67] Finnerty R. McWeeny S. Trainor L. (2023). Online group music therapy: proactive Management of Undergraduate Students’ stress and anxiety. Front. Psych. 14:1183311. doi: 10.3389/fpsyt.2023.1183311, PMID: 37151974 PMC10160410

[ref68] Fujino M. Ueda Y. Mizuhara H. Saiki J. Nomura M. (2018). Open monitoring meditation reduces the involvement of brain regions related to memory function. Sci. Rep. 8:9968. doi: 10.1038/s41598-018-28274-4, PMID: 29967435 PMC6028418

[ref69] Gäbel C. Garrido N. Koenig J. Hillecke T. K. Warth M. (2017). Effects of monochord music on heart rate variability and self-reports of relaxation in healthy adults. Complem. Med. Res. 24, 97–103. doi: 10.1159/000455133, PMID: 28192781

[ref70] Geovanini G. R. Vasques E. R. de Oliveira Alvim R. Mill J. G. Andreão R. V. Vasques B. K.. (2020). Age and sex differences in heart rate variability and vagal specific patterns–Baependi heart study. Glob. Heart 15:71. doi: 10.5334/gh.873, PMID: 33150136 PMC7583712

[ref71] Ghodousi M. Pousson J. E. Voicikas A. Bernhofs V. Pipinis E. Tarailis P.. (2022). EEG connectivity during active emotional musical performance. Sensors 22:4064. doi: 10.3390/s22114064, PMID: 35684685 PMC9185252

[ref72] Goldin P. R. Gross J. J. (2010). Effects of mindfulness-based stress reduction (MBSR) on emotion regulation in social anxiety disorder. Emotion 10, 83–91. doi: 10.1037/a0018441, PMID: 20141305 PMC4203918

[ref73] Gomes N. Pato M. Lourenço A. R. Datia N. (2023). A survey on wearable sensors for mental health monitoring. Sensors (Basel, Switzerland) 23:1330. doi: 10.3390/s23031330, PMID: 36772370 PMC9919280

[ref74] Gorman J. M. Sloan R. P. (2000). Heart rate variability in depressive and anxiety disorders. Am. Heart J. 140, S77–S83. doi: 10.1067/mhj.2000.109981, PMID: 11011352

[ref75] Gosselin N. Peretz I. Johnsen E. Adolphs R. (2007). Amygdala damage impairs emotion recognition from music. Neuropsychologia 45, 236–244. doi: 10.1016/j.neuropsychologia.2006.07.012, PMID: 16970965

[ref76] Griffiths R. R. Johnson M. W. Carducci M. A. Umbricht A. Richards W. A. Richards B. D.. (2016). Psilocybin produces substantial and sustained decreases in depression and anxiety in patients with life-threatening Cancer: a randomized double-blind trial. J. Psychopharmacol. 30, 1181–1197. doi: 10.1177/0269881116675513, PMID: 27909165 PMC5367557

[ref77] Grob C. S. Danforth A. L. Chopra G. S. Hagerty M. McKay C. R. Halberstadt A. L.. (2011). Pilot study of psilocybin treatment for anxiety in patients with advanced-stage Cancer. Arch. Gen. Psychiatry 68, 71–78. doi: 10.1001/archgenpsychiatry.2010.116, PMID: 20819978

[ref78] Gukasyan N. Davis A. K. Barrett F. S. Cosimano M. P. Sepeda N. D. Johnson M. W.. (2022). Efficacy and safety of psilocybin-assisted treatment for major depressive disorder: prospective 12-month follow-up. J. Psychopharmacol. 36, 151–158. doi: 10.1177/02698811211073759, PMID: 35166158 PMC8864328

[ref79] Habibi A. Kreutz G. Russo F. Tervaniemi M. (2022). Music-based interventions in community settings: navigating the tension between rigor and ecological validity. Ann. N. Y. Acad. Sci. 1518, 47–57. doi: 10.1111/nyas.14908, PMID: 36200590 PMC10092011

[ref80] Harmony T. (2013). The functional significance of Delta oscillations in cognitive processing. Front. Integr. Neurosci. 7. doi: 10.3389/fnint.2013.00083, PMID: 24367301 PMC3851789

[ref81] Hartung C. M. Widiger T. A. (1998). Gender differences in the diagnosis of mental disorders: conclusions and controversies of the DSM-IV. Psychol. Bull. 123, 260–278. doi: 10.1037/0033-2909.123.3.260, PMID: 9602559

[ref82] Hashemi A. Pino L. J. Moffat G. Mathewson K. J. Aimone C. Bennett P. J.. (2016). Characterizing Population EEG Dynamics throughout Adulthood. ENeuro 3:ENEURO.0275-16.2016. doi: 10.1523/ENEURO.0275-16.2016, PMID: 27957533 PMC5150228

[ref83] Hernandez-Ruiz E. Dvorak A. L. (2021). Music stimuli for mindfulness practice: a replication study. J. Music. Ther. 58, 155–176. doi: 10.1093/jmt/thaa018, PMID: 33020803

[ref84] Hernandez-Ruiz E. Sebren A. Alderete C. Bradshaw L. Fowler R. (2021). Effect of music on a mindfulness experience: an online study. Arts Psychother. 75:101827. doi: 10.1016/j.aip.2021.101827, PMID: 40083722

[ref85] Hilton L. Hempel S. Ewing B. A. Apaydin E. Xenakis L. Newberry S.. (2017). Mindfulness meditation for chronic pain: systematic review and Meta-analysis. Ann. Behav. Med. 51, 199–213. doi: 10.1007/s12160-016-9844-2 27658913 PMC5368208

[ref86] Hofmann S. G. Gómez A. F. (2017). Mindfulness-based interventions for anxiety and depression. Psychiatr. Clin. North Am. 40, 739–749. doi: 10.1016/j.psc.2017.08.008, PMID: 29080597 PMC5679245

[ref87] Husain Gabriela Thompson William Forde Glenn Schellenberg E. (2002) “Effects of Musical Tempo and Mode on Arousal, Mood, and Spatial Abilities.” Accessed December 31, 2024. Available online at: https://online.ucpress.edu/mp/article/20/2/151/62120/Effects-of-Musical-Tempo-and-Mode-on-Arousal-Mood

[ref88] Hwang M. H. Bunt L. Warner C. (2023). An eight-week Zen meditation and music Programme for mindfulness and happiness: qualitative content analysis. Int. J. Environ. Res. Public Health 20:7140. doi: 10.3390/ijerph20237140, PMID: 38063569 PMC10706294

[ref89] Igbinobaro E. Watts A. Oshodi O. Emile M. C. Soumare A. Takyi P.. (2024). A virtual music mindfulness tool for individuals of African descent during COVID-19. medRxiv. doi: 10.1101/2024.12.23.24305623

[ref90] Ing-Randolph A. R. Phillips L. R. Williams A. B. (2015). Group music interventions for dementia-associated anxiety: a systematic review. Int. J. Nurs. Stud. 52, 1775–1784. doi: 10.1016/j.ijnurstu.2015.06.014, PMID: 26228591

[ref91] Janszky I. Ericson M. Lekander M. Blom M. Buhlin K. Georgiades A.. (2004). Inflammatory markers and heart rate variability in women with coronary heart disease. J. Intern. Med. 256, 421–428. doi: 10.1111/j.1365-2796.2004.01403.x, PMID: 15485478

[ref92] Jones G. Herrmann F. Nock M. K. (2023). A digital music-based mindfulness intervention for Black Americans with elevated race-based anxiety: a multiple-baseline pilot study. JMIR Form. Res. 7:e49284. doi: 10.2196/49284, PMID: 37585252 PMC10468709

[ref93] Jordan A. Babuscio T. Nich C. Carroll K. M. (2021). A feasibility study providing substance use treatment in the Black church. J. Subst. Abus. Treat. 124:108218. doi: 10.1016/j.jsat.2020.108218, PMID: 33771290

[ref94] Kaelen M. Barrett F. S. Roseman L. Lorenz R. Family N. Bolstridge M.. (2015). LSD enhances the emotional response to music. Psychopharmacology 232, 3607–3614. doi: 10.1007/s00213-015-4014-y, PMID: 26257162

[ref95] Kaelen M. Giribaldi B. Raine J. Evans L. Timmerman C. Rodriguez N.. (2018). The hidden therapist: evidence for a central role of music Inpsychedelic therapy. Psychopharmacology 235, 505–519. doi: 10.1007/s00213-017-4820-5, PMID: 29396616 PMC5893695

[ref96] Keller A. S. Leikauf J. E. Holt-Gosselin B. Staveland B. R. Williams L. M. (2019). Paying attention to attention in depression. Transl. Psychiatry 9, 1–12. doi: 10.1038/s41398-019-0616-1, PMID: 31699968 PMC6838308

[ref97] Kent S. Yellowlees P. (1994). Psychiatric and social reasons for frequent Rehospitalization. Psychiatr. Serv. 45, 347–350. doi: 10.1176/ps.45.4.347, PMID: 8020919

[ref98] Kim J. Strohbach C. A. Wedell D. H. (2019). Effects of manipulating the tempo of popular songs on behavioral and physiological responses. Psychol. Music 47, 392–406. doi: 10.1177/0305735618754688

[ref99] Kirk U. Axelsen J. L. (2020). Heart rate variability is enhanced during mindfulness practice: a randomized controlled trial involving a 10-Day online-based mindfulness intervention. PLoS One 15:e0243488. doi: 10.1371/journal.pone.0243488, PMID: 33332403 PMC7746169

[ref100] Kirk U. Ngnoumen C. Clausel A. Purvis C. K. (2022). Effects of three genres of focus music on heart rate variability and sustained attention. J. Cogn. Enhanc. 6, 143–158. doi: 10.1007/s41465-021-00226-3

[ref101] Klose M. Jacobi F. (2004). Can gender differences in the prevalence of mental disorders be explained by sociodemographic factors? Arch. Womens Ment. Health 7, 133–148. doi: 10.1007/s00737-004-0047-7, PMID: 15083348

[ref102] Knoerl R. Mazzola E. Woods H. Buchbinder E. Frazier L. LaCasce A.. (2022). Exploring the feasibility of a mindfulness-music therapy intervention to improve anxiety and stress in adolescents and Young adults with Cancer. J. Pain Symptom Manag. 63, e357–e363. doi: 10.1016/j.jpainsymman.2021.11.013, PMID: 34896280

[ref103] Knyazev G. G. (2012). EEG Delta oscillations as a correlate of basic homeostatic and motivational processes. Neurosci. Biobehav. Rev. 36, 677–695. doi: 10.1016/j.neubiorev.2011.10.002, PMID: 22020231

[ref104] Koelsch S. Cheung V. K. M. Jentschke S. Haynes J.-D. (2021). Neocortical substrates of feelings evoked with music in the ACC, insula, and somatosensory cortex. Sci. Rep. 11:10119. doi: 10.1038/s41598-021-89405-y, PMID: 33980876 PMC8115666

[ref105] Koelsch S. Jäncke L. (2015). Music and the heart. Eur. Heart J. 36, 3043–3049. doi: 10.1093/eurheartj/ehv430 26354957

[ref106] Koelsch S. Maess B. Grossmann T. Friederici A. D. (2003). Electric brain responses reveal gender differences in music processing. Neuro Report 14, 709–713. doi: 10.1097/00001756-200304150-00010, PMID: 12692468

[ref107] Koenig J. Thayer J. F. (2016). Sex differences in healthy human heart rate variability: a Meta-analysis. Neurosci. Biobehav. Rev. 64, 288–310. doi: 10.1016/j.neubiorev.2016.03.007, PMID: 26964804

[ref108] Kroenke K. Spitzer R. L. Williams J. B. (2001). The PHQ-9: validity of a brief depression severity measure. J. Gen. Intern. Med. 16, 606–613. doi: 10.1046/j.1525-1497.2001.016009606.x, PMID: 11556941 PMC1495268

[ref109] Krygier J. R. Heathers J. A. J. Shahrestani S. Abbott M. Gross J. J. Kemp A. H. (2013). Mindfulness meditation, well-being, and heart rate variability: a preliminary investigation into the impact of intensive Vipassana meditation. Int. J. Psychophysiol. 89, 305–313. doi: 10.1016/j.ijpsycho.2013.06.017, PMID: 23797150

[ref110] Kučikienė D. Praninskienė R. (2018). The impact of music on the bioelectrical oscillations of the brain. Acta Medica Lituanica 25, 101–106. doi: 10.6001/actamedica.v25i2.3763, PMID: 30210244 PMC6130927

[ref111] Kume S. Nishimura Y. Mizuno K. Sakimoto N. Hori H. Tamura Y.. (2017). Music improves subjective feelings leading to cardiac autonomic nervous modulation: a pilot study. Front. Neurosci. 11. doi: 10.3389/fnins.2017.00108, PMID: 28344545 PMC5344927

[ref112] Laborde S. Mosley E. Thayer J. F. (2017). Heart rate variability and cardiac vagal tone in psychophysiological research–recommendations for experiment planning, data analysis, and data reporting. Front. Psychol. 8:213. doi: 10.3389/fpsyg.2017.00213, PMID: 28265249 PMC5316555

[ref113] Lampert R. Douglas Bremner J. Shaoyong S. Miller A. Lee F. Cheema F.. (2008). Decreased heart rate variability is associated with higher levels of inflammation in middle-aged men. Am. Heart J. 156, 759.e1–759.e7. doi: 10.1016/j.ahj.2008.07.009, PMID: 18926158 PMC2587932

[ref114] Latta F. Leproult R. Tasali E. Hofmann E. Van Cauter E. (2005). Sex differences in Delta and alpha EEG activities in healthy older adults. Sleep 28, 1525–1534. doi: 10.1093/sleep/28.12.1525, PMID: 16408411

[ref115] Lee E.-J. Bhattacharya J. (2013). Heart rate variability during monochord-induced relaxation in female patients with Cancer undergoing chemotherapy. Music Med. 5, 177–186. doi: 10.47513/mmd.v5i3.321

[ref116] Lee E.-J. Bhattacharya J. Sohn C. Verres R. (2012). Monochord sounds and progressive muscle relaxation reduce anxiety and improve relaxation during chemotherapy: a pilot EEG study. Complement. Ther. Med. 20, 409–416. doi: 10.1016/j.ctim.2012.07.002 23131371

[ref117] Lee R. M. Robbins S. B. (1995). Measuring belongingness: the social connectedness and the social assurance scales. J. Couns. Psychol. 42, 232–241. doi: 10.1037/0022-0167.42.2.232

[ref118] Lesiuk T. (2015). The effect of mindfulness-based music therapy on attention and mood in women receiving adjuvant chemotherapy for breast Cancer: a pilot study. Oncol. Nurs. Forum 42, 276–282. doi: 10.1188/15.ONF.276-282, PMID: 25901379

[ref119] Li J. Zheng L. (2022). The mechanism of cardiac sympathetic activity assessment methods: current knowledge. Front. Cardiovasc. Med. 9:931219. doi: 10.3389/fcvm.2022.931219, PMID: 35811701 PMC9262089

[ref120] Liao D. Barnes R. W. Chambless L. E. Simpson R. J. Sorlie P. Heiss G.. (1995). Age, race, and sex differences in autonomic cardiac function measured by spectral analysis of heart rate variability—the ARIC study. Am. J. Cardiol. 76, 906–912. doi: 10.1016/S0002-9149(99)80260-4, PMID: 7484830

[ref121] Lippelt D. P. Hommel B. Colzato L. S. (2014). Focused attention, open monitoring and loving kindness meditation: effects on attention, conflict monitoring, and creativity – a review. Front. Psychol. 5:1083. doi: 10.3389/fpsyg.2014.01083, PMID: 25295025 PMC4171985

[ref122] Liu H. Gao X. Hou Y. (2019). Effects of mindfulness-based stress reduction combined with music therapy on pain, anxiety, and sleep quality in patients with osteosarcoma. Revista Brasileira De Psiquiatria (Sao Paulo, Brazil: 1999) 41, 540–545. doi: 10.1590/1516-4446-2018-0346, PMID: 31116262 PMC6899366

[ref123] Liu X. Liu Y. Shi H. Li L. Zheng M. (2021a). Regulation of mindfulness-based music listening on negative emotions related to COVID-19: an ERP study. Int. J. Environ. Res. Public Health 18:7063. doi: 10.3390/ijerph18137063, PMID: 34280999 PMC8296951

[ref124] Liu X. Liu Y. Shi H. Zheng M. (2021b). Effects of mindfulness meditation on musical aesthetic emotion processing. Front. Psychol. 12:648062. doi: 10.3389/fpsyg.2021.648062, PMID: 34366968 PMC8334183

[ref125] Lomas T. Ivtzan I. Fu C. H. Y. (2015). A systematic review of the neurophysiology of mindfulness on EEG oscillations. Neurosci. Biobehav. Rev. 57, 401–410. doi: 10.1016/j.neubiorev.2015.09.018, PMID: 26441373

[ref126] Lothes I. I. John E. Matney S. Naseer Z. (2022). Sitting meditation (mindfulness) and music meditation effects on overall anxiety and test anxiety in a college student population. Build. Healthy Acad. Commun. J. 6, 29–46. doi: 10.18061/bhac.v6i1.8686

[ref127] Ludwig A. M. (1966). Altered states of consciousness. Arch. Gen. Psychiatry 15, 225–234. doi: 10.1001/archpsyc.1966.01730150001001 5330058

[ref128] Mahmood D. Nisar H. Yap V. V. Tsai C.-Y. (2022). The effect of music listening on EEG functional connectivity of brain: a short-duration and long-duration study. Mathematics 10:349. doi: 10.3390/math10030349

[ref129] Maidhof C. Müller V. Lartillot O. Agres K. Bloska J. Asano R.. (2023). Intra-and inter-brain coupling and activity dynamics during improvisational music therapy with a person with dementia: an explorative EEG-Hyperscanning single case study. Front. Psychol. 14:1155732. doi: 10.3389/fpsyg.2023.1155732, PMID: 37842703 PMC10570426

[ref130] Maldonato M. N. Sperandeo R. Dell’Orco S. Iennaco D. Cerroni F. Romano P.. (2018). Mind, brain and altered states of consciousness. Acta Medica 34:357. doi: 10.19193/0393-6384_2018_2_56

[ref131] Malik M. Thomas Bigger J. John Camm A. Kleiger R. E. Malliani A. Moss A. J.. (1996). Heart rate variability: standards of measurement, physiological interpretation, and clinical use. Eur. Heart J. 17, 354–381. doi: 10.1093/oxfordjournals.eurheartj.a014868 8737210

[ref132] Manna A. Raffone A. Perrucci M. G. Nardo D. Ferretti A. Tartaro A.. (2010). Neural correlates of focused attention and cognitive monitoring in meditation. Brain Res. Bull. 82, 46–56. doi: 10.1016/j.brainresbull.2010.03.001, PMID: 20223285

[ref133] Maratos Anna Gold Christian Xu Wang Crawford Mike. (2008) “Music Therapy for Depression. Maratos A Cochrane Library.” (Accessed December 8, 2024). Available online at: https://www.cochranelibrary.com/cdsr/doi/10.1002/14651858.CD004517.pub2/full

[ref134] Marini M. M. (1990). Sex and gender: what do we know? Sociol. Forum 5, 95–120. doi: 10.1007/BF01115139

[ref135] Matko K. Sedlmeier P. (2019). What is meditation? Proposing an empirically derived classification system. Front. Psychol. 10:2276. doi: 10.3389/fpsyg.2019.02276, PMID: 31681085 PMC6803504

[ref136] McCrary J. M. Altenmüller E. (2021). Mechanisms of music impact: autonomic tone and the physical activity roadmap to advancing understanding and evidence-based policy. Front. Psychol. 12:727231. doi: 10.3389/fpsyg.2021.727231, PMID: 34512483 PMC8429896

[ref137] McPherson T. Berger D. Alagapan S. Fröhlich F. (2019). Active and passive rhythmic music therapy interventions differentially modulate sympathetic autonomic nervous system activity. J. Music. Ther. 56, 240–264. doi: 10.1093/jmt/thz007, PMID: 31175814 PMC6693240

[ref138] Meling D. Egger K. Aicher H. D. Redondo J. J. Mueller J. Dornbierer J.. (2024). Meditating on psychedelics. A randomized placebo-controlled study of DMT and Harmine in a mindfulness retreat. J. Psychopharmacol. 38, 897–910. doi: 10.1177/02698811241282637, PMID: 39340164 PMC11487865

[ref139] Mitcheff M. Khan A. Imtiaz R. (2021). Physiological and psychological reactions to a musician, robot, or Boombox music player: comparison between EDA, HRV, and EEG. Neurol. Res. Surg. 4, 1–8. doi: 10.33425/2641-4333.1041

[ref140] Mitchell J. M. Bogenschutz M. Lilienstein A. Harrison C. Kleiman S. Parker-Guilbert K.. (2023). MDMA-assisted therapy for severe PTSD: a randomized, double-blind, placebo-controlled phase 3 study. Focus 21, 315–328. doi: 10.1176/appi.focus.23021011, PMID: 37404971 PMC10316215

[ref141] Monfredi O. Lyashkov A. E. Johnsen A.-B. Inada S. Schneider H. Wang R.. (2014). Biophysical characterization of the underappreciated and important relationship between heart rate variability and heart rate. Hypertension 64, 1334–1343. doi: 10.1161/HYPERTENSIONAHA.114.03782, PMID: 25225208 PMC4326239

[ref142] Moon E. Lee S.-H. Kim D.-H. Hwang B. (2013). Comparative study of heart rate variability in patients with schizophrenia, bipolar disorder, post-traumatic stress disorder, or major depressive disorder. Clin. Psychopharma. Neurosci. 11, 137–143. doi: 10.9758/cpn.2013.11.3.137 PMC3897762 24465250

[ref143] Moore A. Gruber T. Derose J. Malinowski P. (2012). Regular, brief mindfulness meditation practice improves electrophysiological markers of attentional control. Front. Hum. Neurosci. 6:18. doi: 10.3389/fnhum.2012.00018, PMID: 22363278 PMC3277272

[ref144] Morey R. A. Haswell C. C. Hooper S. R. De Bellis M. D. (2016). Amygdala, Hippocampus, and ventral medial prefrontal cortex volumes differ in maltreated youth with and without chronic posttraumatic stress disorder. Neuropsychopharmacology 41, 791–801. doi: 10.1038/npp.2015.205, PMID: 26171720 PMC4707825

[ref145] Nakashima M. Ebihara N. Nishijo H. Ohira H. (2013). The effect of music on psychological and physiological stress. J. Hum. Environ. Stud. 11, 19–25. doi: 10.4189/shes.11.19

[ref146] Nanthakwang N. Siviroj P. Matanasarawoot A. Sapbamrer R. Lerttrakarnnon P. Awiphan R. (2020). Effectiveness of deep breathing and body scan meditation combined with music to improve sleep quality and quality of life in older adults. Open Public Health J. 13, 232–239. doi: 10.2174/1874944502013010232

[ref147] Newman E. Kaloupek D. G. (2004). The risks and benefits of participating in trauma-focused research studies. J. Trauma. Stress. 17, 383–394. doi: 10.1023/B:JOTS.0000048951.02568.3a, PMID: 15633917

[ref148] Nijjar P. S. Puppala V. K. Dickinson O. Duval S. Duprez D. Kreitzer M. J.. (2014). Modulation of the autonomic nervous system assessed through heart rate variability by a mindfulness based stress reduction program. Int. J. Cardiol. 177, 557–559. doi: 10.1016/j.ijcard.2014.08.116, PMID: 25179555

[ref149] Nilsson U. (2008). The anxiety-and pain-reducing effects of music interventions: a systematic review. AORN J. 87, 780–807. doi: 10.1016/j.aorn.2007.09.013, PMID: 18395022

[ref150] Ooishi Y. Mukai H. Watanabe K. Kawato S. Kashino M. (2017). Increase in salivary oxytocin and decrease in salivary cortisol after listening to relaxing slow-tempo and exciting fast-tempo music. PLoS One 12:e0189075. doi: 10.1371/journal.pone.0189075, PMID: 29211795 PMC5718605

[ref151] Oswald V. Vanhaudenhuyse A. Annen J. Martial C. Bicego A. Rousseaux F.. (2023). Autonomic nervous system modulation during self-induced non-ordinary states of consciousness. Sci. Rep. 13:15811. doi: 10.1038/s41598-023-42393-7 37737222 PMC10516905

[ref152] Pavlygina R. A. Sakharov D. S. Davydov V. I. (2004). Spectral analysis of the human EEG during listening to musical compositions. Hum. Physiol. 30, 54–60. doi: 10.1023/B:HUMP.0000013765.64276.e6 15040288

[ref153] Peng C.-K. Henry I. C. Mietus J. E. Hausdorff J. M. Khalsa G. Benson H.. (2004). Heart rate dynamics during three forms of meditation. Int. J. Cardiol. 95, 19–27. doi: 10.1016/j.ijcard.2003.02.006, PMID: 15159033

[ref154] Pham T. Lau Z. J. Annabel Chen S. H. Makowski D. (2021). Heart rate variability in psychology: a review of HRV indices and an analysis tutorial. Sensors (Basel, Switzerland) 21:3998. doi: 10.3390/s21123998, PMID: 34207927 PMC8230044

[ref155] Phillips C. S. Bockhoff J. Berry D. L. Elizabeth Buchbinder A. Frazier L. LaCasce A.. (2023). Exploring Young adults’ perspectives of participation in a mindfulness-based music therapy intervention before and during the COVID-19 pandemic. J. Adolesc. Young Adult Oncol. 12, 569–576. doi: 10.1089/jayao.2022.0090, PMID: 36752714

[ref156] Qi W. Zhao F. Huang S. Wei Z. Yang H. He K.. (2023). Effects and feasibility of a mindfulness-based Guqin music intervention during pregnancy on postpartum anxiety and depression: a pilot randomized controlled trial. Mindfulness 14, 2641–2656. doi: 10.1007/s12671-023-02221-5

[ref157] Rabinowicz T. Dean D. E. Petetot J. M. D.-C. de Courten-Myers G. M. (1999). Gender differences in the human cerebral cortex: more neurons in males; more processes in females. J. Child Neurol. 14, 98–107. doi: 10.1177/088307389901400207, PMID: 10073431

[ref158] Radakovic C. Radakovic R. Peryer G. Geere J.-A. (2022). Psychedelics and mindfulness: a systematic review and Meta-analysis. J. Psych. Stud. 6, 137–153. doi: 10.1556/2054.2022.00218, PMID: 29951292

[ref159] Ramesh A. Nayak T. Beestrum M. Quer G. Pandit J. A. (2023). Heart rate variability in psychiatric disorders: a systematic review. Neuropsychiatr. Dis. Treat. 19, 2217–2239. doi: 10.2147/NDT.S429592, PMID: 37881808 PMC10596135

[ref160] Reangsing C. Trakooltorwong P. Maneekunwong K. Thepsaw J. Oerther S. (2023). Effects of online mindfulness-based interventions (MBIs) on anxiety symptoms in adults: a systematic review and Meta-analysis. BMC Complement. Med. Ther. 23:269. doi: 10.1186/s12906-023-04102-9, PMID: 37507747 PMC10386675

[ref161] Rich-Edwards J. W. Kaiser U. B. Chen G. L. Manson J. A. E. Goldstein J. M. (2018). Sex and gender differences research Design for Basic, clinical, and population studies: essentials for Investigators. Endocr. Rev. 39, 424–439. doi: 10.1210/er.2017-00246, PMID: 29668873 PMC7263836

[ref162] Roy A. Hoge E. A. Abrante P. Druker S. Liu T. Brewer J. A. (2021). Clinical efficacy and psychological mechanisms of an app-based digital therapeutic for generalized anxiety disorder: randomized controlled trial. J. Med. Internet Res. 23:e26987. doi: 10.2196/26987, PMID: 34860673 PMC8686411

[ref163] Rubia K. (2009). The neurobiology of meditation and its clinical effectiveness in psychiatric disorders. Biol. Psychol. 82, 1–11. doi: 10.1016/j.biopsycho.2009.04.003, PMID: 19393712

[ref164] Saddichha S. Al-Desouki M. Lamia A. Linden I. A. Krausz M. (2014). Online interventions for depression and anxiety – a systematic review. Health Psychol. Behav. Med. 2, 841–881. doi: 10.1080/21642850.2014.945934, PMID: 25750823 PMC4346073

[ref165] Salimpoor V. N. Benovoy M. Larcher K. Dagher A. Zatorre R. J. (2011). Anatomically distinct dopamine release during anticipation and experience of peak emotion to music. Nat. Neurosci. 14, 257–262. doi: 10.1038/nn.2726, PMID: 21217764

[ref166] Salimpoor V. N. Benovoy M. Longo G. Cooperstock J. R. Zatorre R. J. (2009). The rewarding aspects of music listening are related to degree of emotional arousal. PLoS One 4:e7487. doi: 10.1371/journal.pone.0007487, PMID: 19834599 PMC2759002

[ref167] Sammler D. Grigutsch M. Fritz T. Koelsch S. (2007). Music and emotion: electrophysiological correlates of the processing of pleasant and unpleasant music. Psychophysiology 44, 293–304. doi: 10.1111/j.1469-8986.2007.00497.x, PMID: 17343712

[ref168] Schmidt T. T. Berkemeyer H. (2018). The altered states database: psychometric data of altered states of consciousness. Front. Psychol. 9:1028. doi: 10.3389/fpsyg.2018.01028, PMID: 30013493 PMC6036510

[ref169] Seamans J. K. Floresco S. B. (2022). Event-based control of autonomic and emotional states by the anterior cingulate cortex. Neurosci. Biobehav. Rev. 133:104503. doi: 10.1016/j.neubiorev.2021.12.026, PMID: 34922986

[ref170] Sgoifo A. Carnevali L. Pico Alfonso M. D. Amore M. (2015). Autonomic dysfunction and heart rate variability in depression. Stress 18, 343–352. doi: 10.3109/10253890.2015.1045868, PMID: 26004818

[ref171] Sheppard E. (2024). “The therapeutic power of altered states of consciousness” in Mild altered states of consciousness: Subtle shifts of mind and their therapeutic potential. ed. Sheppard E. (Cham: Springer International Publishing), 277–294.

[ref172] Smigielski L. Kometer M. Scheidegger M. Krähenmann R. Huber T. Vollenweider F. X. (2019). Characterization and prediction of acute and sustained response to psychedelic psilocybin in a mindfulness group retreat. Sci. Rep. 9:14914. doi: 10.1038/s41598-019-50612-3, PMID: 31649304 PMC6813317

[ref173] Smoller J. W. (2016). The genetics of stress-related disorders: PTSD, depression, and anxiety disorders. Neuropsychopharmacology 41, 297–319. doi: 10.1038/npp.2015.266, PMID: 26321314 PMC4677147

[ref174] Soo M. S. Jarosz J. A. Wren A. A. Soo A. E. Mowery Y. M. Johnson K. S.. (2016). Imaging-guided Core-needle breast biopsy: impact of meditation and music interventions on patient anxiety, pain, and fatigue. J. Am. Coll. Radiol. 13, 526–534. doi: 10.1016/j.jacr.2015.12.004, PMID: 26853501

[ref175] Sorensen S. Steindl S. R. Dingle G. A. Garcia A. (2019). Comparing the effects of loving-kindness meditation (LKM), music and LKM plus music on psychological well-being. J. Psychol. 153, 267–287. doi: 10.1080/00223980.2018.1516610, PMID: 30592696

[ref176] Sowell E. R. Peterson B. S. Kan E. Woods R. P. Yoshii J. Bansal R.. (2007). Sex differences in cortical thickness mapped in 176 healthy individuals between 7 and 87 years of age. Cereb. Cortex 17, 1550–1560. doi: 10.1093/cercor/bhl066, PMID: 16945978 PMC2329809

[ref177] Spironelli C. Angrilli A. (2010). Developmental aspects of language lateralization in Delta, Theta, alpha and Beta EEG bands. Biol. Psychol. 85, 258–267. doi: 10.1016/j.biopsycho.2010.07.011, PMID: 20659528

[ref178] Spitzer R. L. Kroenke K. Williams J. B. W. Löwe B. (2006). A brief measure for assessing generalized anxiety disorder: the GAD-7. Arch. Intern. Med. 166, 1092–1097. doi: 10.1001/archinte.166.10.1092, PMID: 16717171

[ref179] Studerus E. Gamma A. Vollenweider F. X. (2010). Psychometric evaluation of the altered states of consciousness rating scale (OAV). PLoS One 5:e12412. doi: 10.1371/journal.pone.0012412, PMID: 20824211 PMC2930851

[ref180] Swarbrick D. Bosnyak D. Livingstone S. R. Bansal J. Marsh-Rollo S. Woolhouse M. H.. (2019). How live music moves us: head movement differences in audiences to live versus recorded music. Front. Psychol. 9:2682. doi: 10.3389/fpsyg.2018.02682, PMID: 30687158 PMC6336707

[ref181] Tanay G. Bernstein A. (2013). State mindfulness scale (SMS): development and initial validation. Psychol. Assess. 25, 1286–1299. doi: 10.1037/a0034044, PMID: 24059475

[ref182] Tang Y.-Y. Ma Y. Fan Y. Feng H. Wang J. Feng S.. (2009). Central and autonomic nervous system interaction is altered by short-term meditation. Proc. Natl. Acad. Sci. 106, 8865–8870. doi: 10.1073/pnas.0904031106, PMID: 19451642 PMC2690030

[ref183] Thayer J. F. Friedman B. H. Borkovec T. D. (1996). Autonomic characteristics of generalized anxiety disorder and worry. Biol. Psychiatry 39, 255–266. doi: 10.1016/0006-3223(95)00136-0 8645772

[ref184] Theofanopoulou C. Paez S. Huber D. Todd E. Ramírez-Moreno M. A. Khaleghian B.. (2024). Mobile brain imaging in Butoh dancers: from rehearsals to public performance. BMC Neurosci. 25:62. doi: 10.1186/s12868-024-00864-1, PMID: 39506628 PMC11539292

[ref185] Thoret E. Caramiaux B. Depalle P. McAdams S. (2021). Learning metrics on Spectrotemporal modulations reveals the perception of musical instrument timbre. Nat. Hum. Behav. 5, 369–377. doi: 10.1038/s41562-020-00987-5, PMID: 33257878

[ref186] Unger R. K. (1979). Toward a redefinition of sex and gender. Am. Psychol. 34, 1085–1094. doi: 10.1037/0003-066X.34.11.1085

[ref187] Usui H. Nishida Y. (2017). The very low-frequency band of heart rate variability represents the slow recovery component after a mental stress task. PLoS One 12:e0182611. doi: 10.1371/journal.pone.0182611, PMID: 28806776 PMC5555691

[ref188] Vago D. R. David S. A. (2012). Self-awareness, self-regulation, and self-transcendence (S-ART): a framework for understanding the neurobiological mechanisms of mindfulness. Front. Hum. Neurosci. 6:296. doi: 10.3389/fnhum.2012.00296, PMID: 23112770 PMC3480633

[ref189] Vidyarthi Jay Riecke Bernhard E. Gromala Diane. (2012). “Sonic cradle: designing for an immersive experience of meditation by connecting respiration to music.” In Proceedings of the designing interactive systems conference, 408–417. DIS’12. New York, NY, USA: Association for Computing Machinery.

[ref190] Watanabe K. Ooishi Y. Kashino M. (2017). Heart rate responses induced by acoustic tempo and its interaction with basal heart rate. Sci. Rep. 7:43856. doi: 10.1038/srep43856, PMID: 28266647 PMC5339732

[ref191] Wessel D. L. (1979). Timbre space as a musical control structure. Comput. Music. J. 3, 45–52. doi: 10.2307/3680283

[ref192] Xiao X. Chen W. Zhang X. (2023). The effect and mechanisms of music therapy on the autonomic nervous system and brain networks of patients of minimal conscious states: a randomized controlled trial. Front. Neurosci. 17:1182181. doi: 10.3389/fnins.2023.1182181, PMID: 37250411 PMC10213399

[ref193] Young D. W. (2014). Self-measure of heart rate variability (HRV) and arrhythmia to monitor and to manage atrial arrhythmias: personal experience with high intensity interval exercise (HIIE) for the conversion to sinus rhythm. Front. Physiol. 5:251. doi: 10.3389/fphys.2014.00251, PMID: 25071596 PMC4085876

