# Supplementary material for: Music mindfulness acutely modulates autonomic activity and improves psychological state in anxiety and depression
Source: Front Neurosci. 2025 Apr 8;19:1554156. doi: 10.3389/fnins.2025.1554156 (PMC12013341; doi:10.3389/fnins.2025.1554156)
Supplement: Supplementary file 1 [file Data_Sheet_1.pdf]

Supplementary Material

Supplementary Figures

Supplementary Figure 1

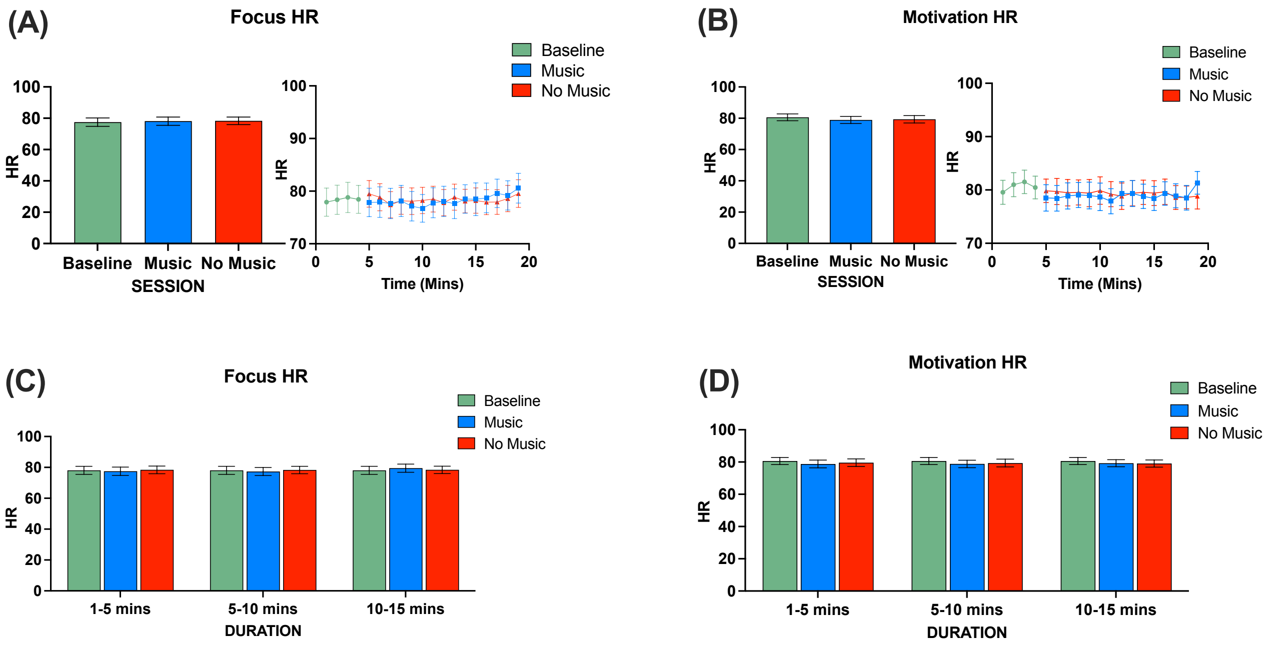

Supplementary Figure 1. **Heart rate during Music Mindfulness sessions**
(A) HR during focus for entire 15-minute duration. **Left** 1-way repeated measures ANOVA showed no significance ((F (2, 54) = 0.05074, P=0.9506)). **Right** the differences between the slopes are significant (Linear regression, F = 4.723. DFn = 2, DFd = 28, p value=0.0171). **(B)** HR during motivation. **Left** 1-way repeated measures ANOVA showed no significance (F (2, 60) = 0.1523, p value=0.8591). Right - the differences between the slopes are significant (F = 4.828. DFn = 2, DFd = 28, p value=0.0158). **(C)** HR during focus for five-minute intervals. 2-way repeated measures ANOVA showed significance in Time x Session (F (4, 108) = 3.346, P=0.0127), Time (F (1.826, 98.58) = 3.770, P=0.0300), and Participants (F (54, 108) = 146.4, P<0.0001). **(D)** HR during motivation for five-minute intervals. 2-way repeated measures ANOVA showed significance in Participants (F (60, 120) = 147.5, p value<0.0001).

Supplementary Figure 2

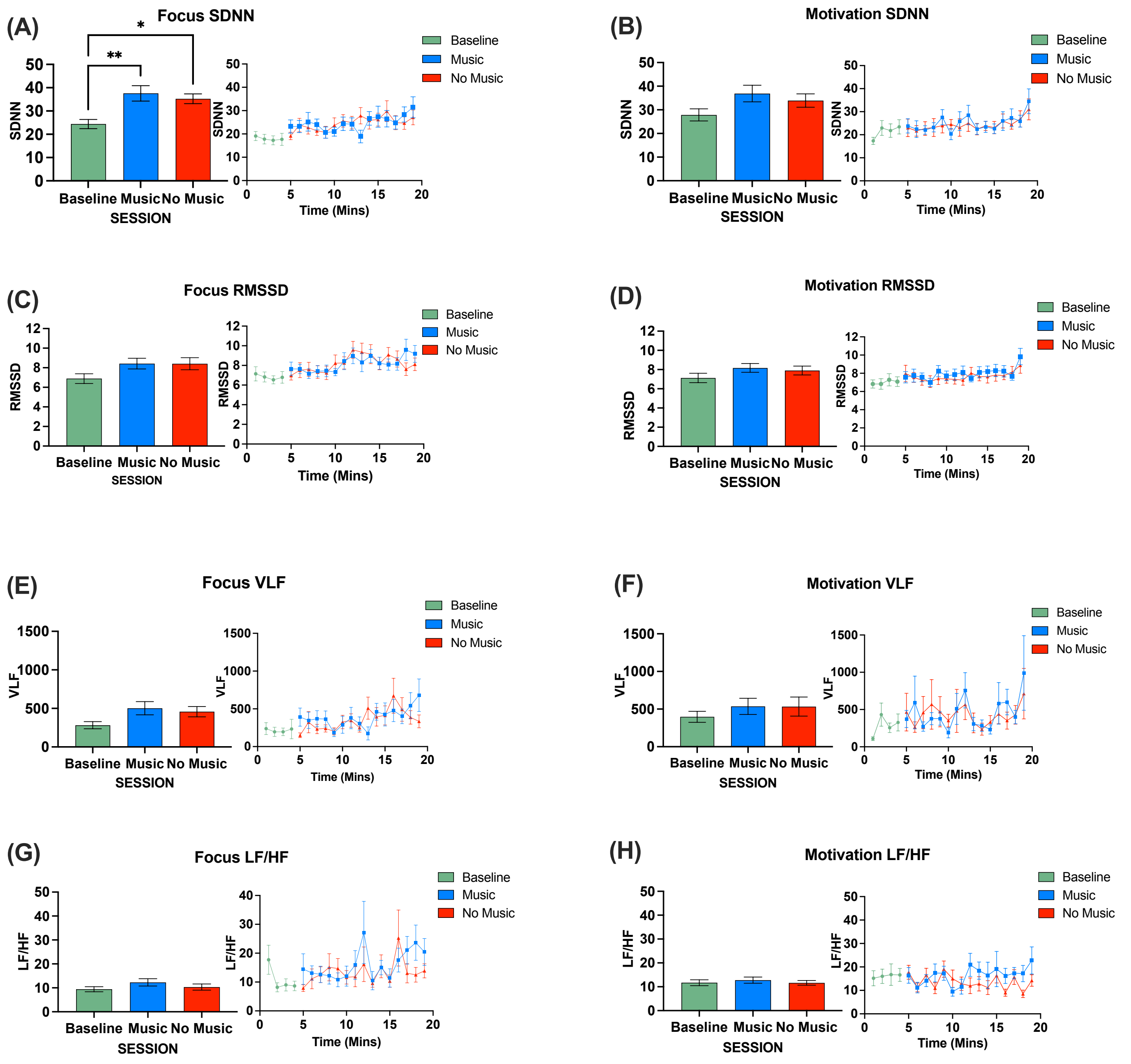

Supplementary Figure 2. **HRV metrics calculated for the entire duration of Focus and Motivation sessions**

(A) SDNN during focus for 15 minutes duration. **Left** 1-way repeated measures ANOVA showed significance in Session (F (2, 54) = 7.712, P=0.0011). Tukey's post hoc multiple comparisons test showed significance between minutes 10 to 15 (q = 5.203; **p value = 0.0015, q = 4.285; *p value = 0.0103)). **Right** - the differences between the slopes are not significant (F = 0.2407. DFn = 2, DFd = 640, P=0.7862).

(B) SDNN during motivation for 15 minutes duration. **Left** 1-way repeated measures ANOVA showed no significance (F (2, 60) = 2.351, P=0.1040). **Right** - the differences between the slopes are not significant (F = 0.7634. DFn = 2, DFd = 28, P=0.4755).

(C) RMSSD during focus for 15 minutes duration. **Left** 1-way repeated measures ANOVA showed no significance (F (2, 54) = 2.496, P=0.0919). **Right** - the differences between the slopes are not significant (F = 0.4803. DFn = 2, DFd = 640, P=0.6188).

(D) RMSSD during motivation for 15 minutes duration. **Left** 1-way repeated measures ANOVA showed no significance (F (2, 60) = 1.325, P=0.2735). **Right** - the differences between the slopes are not significant (F = 0.2890. DFn = 2, DFd = 28, P=0.7513).
(E) VLF during focus for 15 minutes duration. **Left** 1-way repeated measures ANOVA showed no significance (F (2, 54) = 2.909, P=0.0631). **Right** - the differences between the slopes are not significant (F = 0.1697. DFn = 2, DFd = 28, P=0.8448).
(F) VLF during motivation for 15 minutes duration. **Left** 1-way repeated measures ANOVA showed no significance (F (2, 60) = 0.5681, P=0.5696). **Right** - the differences between the slopes are not significant (F = 0.6710. DFn = 2, DFd = 28, P=0.5192).
(G) LF/HF during focus for 15 minutes duration. **Left** 1-way repeated measures ANOVA showed no significance (F (2, 54) = 1.255, P=0.2931). **Right** - the differences between the slopes are not significant (F = 0.1697. DFn = 2, DFd = 28, P=0.8448).
(H) LF/HF during motivation for 15 minutes duration. **Left** 1-way repeated measures ANOVA showed no significance (F (2, 60) = 0.2864, P=0.7520). **Right** - the differences between the slopes are significant (F = 3.484. DFn = 2, DFd = 28, P=0.0445).

Supplementary Figure 3

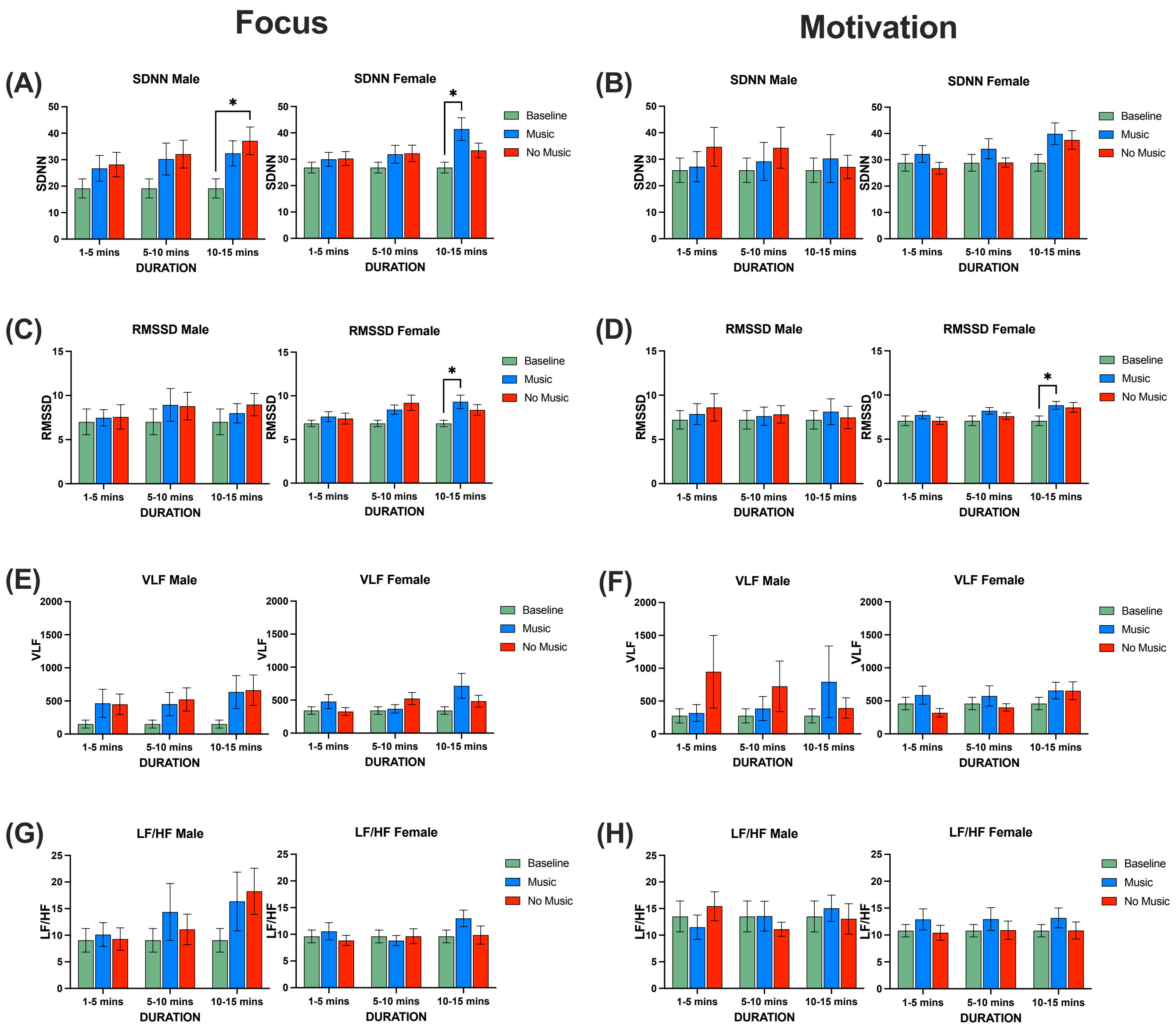

Supplementary Figure 3. Comparison of time and frequency domain HRV metrics for males and females during Focus and Motivation sessions

(A) ***Left:*** SDNN during focus for all males (n=6) was significantly greater than baseline for no music. 2-way repeated measures ANOVA showed significance in Time (F (1.654, 24.81) = 4.684, P=0.0241) and Participants (F (15, 30) = 15.32, P<0.0001). Tukey's post hoc multiple comparisons test showed significance for no music between minutes 10 to 15 (q = 3.999; *p value = 0.0478).
Right: SDNN during focus for all females (n=13) was significantly greater than baseline for music. 2-way repeated measures ANOVA showed significance in Time x Session (F (4, 72) = 3.379, P=0.0137, Time (F (1.856, 66.81) = 6.044, P=0.0047), and Participants (F (36, 72) = 5.922, P<0.0001). Tukey's post hoc multiple comparisons test showed significance between minutes 10 to 15 for music (q = 4.299; *p value = 0.0190).
(B) ***Left:*** SDNN during motivation for all males (n=7); 2-way repeated measures ANOVA showed significance in Participants (F (18, 36) = 29.96, p value<0.0001).

Right: SDNN during motivation for all females (n=14) ; 2-way repeated measures ANOVA showed significance in Time (F (1.679, 65.47) = 7.851 = 6.044, p value=0.0016), and Participants (F (39, 78) = 5.789, p value<0.0001).
(C) ***Left:*** RMSSD during focus for all males; 2-way repeated measures ANOVA showed significance in Participants (F (15, 30) = 29.08, p value<0.0001).
Right: RMSSD during focus for all females was significantly greater than baseline for music. 2-way repeated measures ANOVA showed significance in Time x Session (F (4, 72) = 6.125, p value=0.0003), Time F (1.726, 62.15) = 12.61, p value<0.0001), and Participants (F (36, 72) = 14.88, p value<0.0001). Tukey's post hoc multiple comparisons test showed significance between minutes 10 to 15 for music (q = 4.108; *p value = 0.0251).
(D) ***Left:*** RMSSD during motivation for all males; 2-way repeated measures ANOVA showed significance in Participants (F (18, 36) = 41.61, p value<0.0001).
Right: RMSSD during motivation for all females was significantly greater than baseline for music. 2-way repeated measures ANOVA showed significance in Time x Session (F (4, 78) = 2.627, p value=0.0407), Time (F (1.585, 61.81) = 9.684, p value=0.0006), and Participants (F (39, 78) = 9.231, p value<0.0001). Tukey's post hoc multiple comparisons test showed significance between minutes 10 to 15 for music (q = 3.545; *p value = 0.0484).
(E) ***Left:*** VLF during focus for all males; 2-way repeated measures ANOVA showed significance in Participants (F (15, 30) = 14.15, P<0.0001).
Right: VLF during focus for all females; 2-way repeated measures ANOVA showed significance in Time x Session (F (4, 72) = 3.425, p value=0.0128), Time (F (1.766, 63.58) = 3.465, p value=0.0428), and Participants (F (36, 72) = 4.470, p value<0.0001).
(F) ***Left:*** VLF during motivation for all males; 2-way repeated measures ANOVA showed significance in Participants (F (18, 36) = 6.621, p value<0.0001).
Right: VLF during focus for all females; 2-way repeated measures ANOVA showed significance in Participants (F (39, 78) = 5.160, p value<0.0001).
(G) ***Left:*** LF/HF during focus for all males; 2-way repeated measures ANOVA showed significance in Time (F (1.876, 28.14) = 6.678, P=0.0049), and Participants (F (15, 30) = 10.59, P<0.0001).
Right: LF/HF during focus for all females; 2-way repeated measures ANOVA showed significance in Participants (F (36, 72) = 4.765, p value<0.0001).
(H) ***Left:*** LF/HF during motivation for all males; 2-way repeated measures ANOVA showed significance in Participants (F (18, 36) = 8.111, p value<0.0001).
Right: LF/HF during motivation for all females; 2-way repeated measures ANOVA showed significance in Participants (F (39, 78) = 5.192, p value<0.0001).
SDNN: standard deviation of the normal-normal intervals, RMSSD: root mean square of successive interval differences. VLF: very low frequency power, LF/HF: ratio of low frequency power to high frequency power. Error bars denote standard error of the mean, SEM.

Supplementary Figure 4

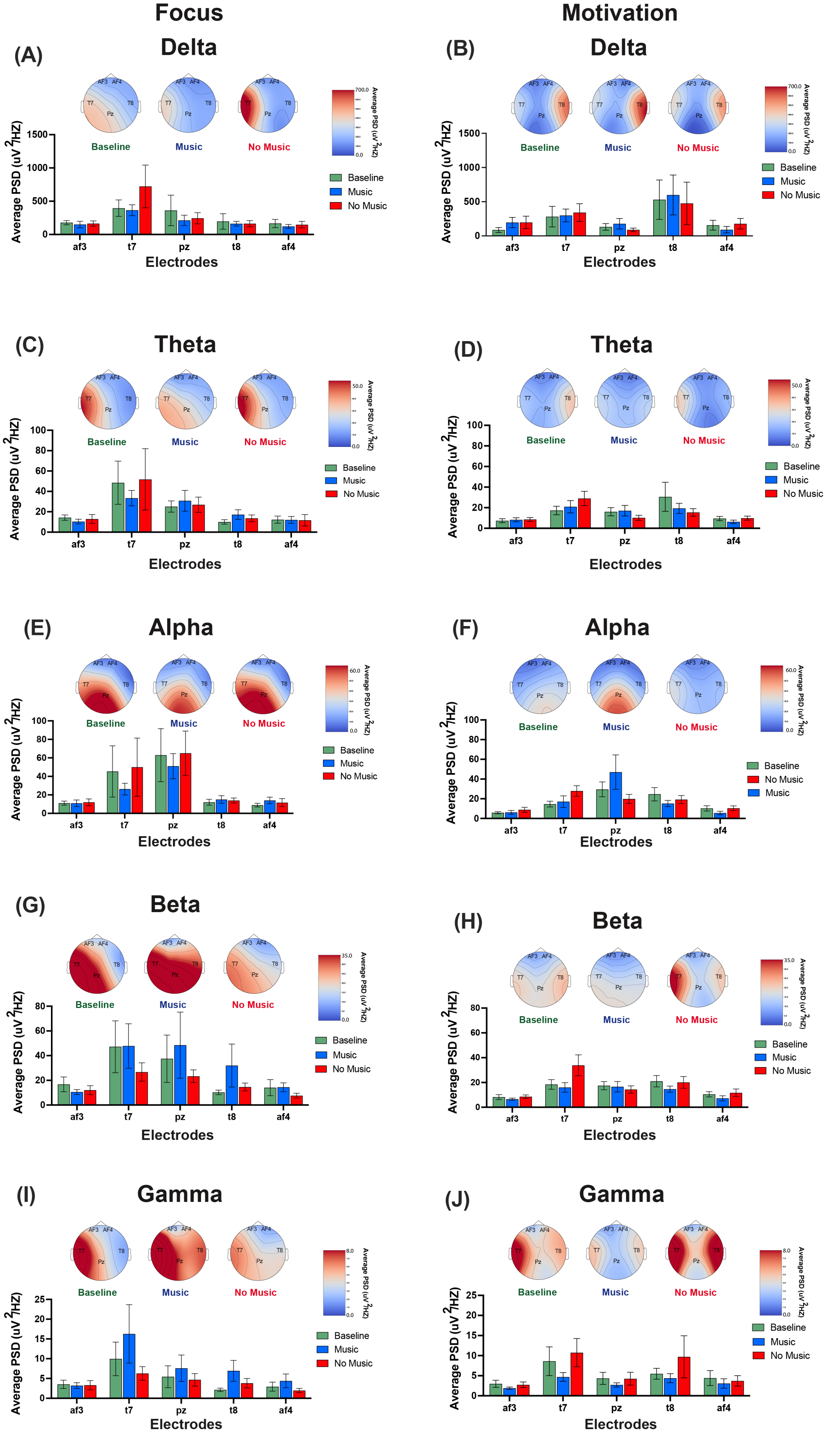

Supplementary Figure 4. Group-average power spectral density (PSD) and EEG topographic maps for Focus (left column) and Motivation (right column) conditions across Baseline, Music and No Music sessions

(A) Delta frequency band for Focus Baseline, Music and No Music sessions. The Friedman test did not show significant difference in PSD across electrodes during Baseline sessions, χ^2=6.1778, p-value=0.1862. There was no significant difference in PSD across electrodes during Music sessions, χ^2=8.7555, p-value=0.0675. There was no significant difference in PSD across electrodes during No Music session, χ^2=2.4444, p-value=0.6546.

(B) Theta frequency band for Focus Baseline, Music and No Music sessions. The Friedman test showed a significant difference in PSD across electrodes during Baseline sessions, χ^2=11.2444, p-value=0.0239. There was a significant difference in PSD across electrodes during Music sessions, χ^2=13.6444, p-value=0.0085. There was a significant difference in PSD across electrodes during No Music sessions, χ^2=10.3111, p-value=0.0355.

(C) Alpha frequency band for Focus Baseline, Music and No Music sessions. The Friedman test showed a significant difference in PSD across electrodes during Baseline sessions, χ^2=17.4222, p-value=0.0016. There was a significant difference in PSD across electrodes during Music sessions, χ^2=15.7777, p-value=0.0033. There was a significant difference in PSD across electrodes during No Music sessions, χ^2=25.4667, p-value=0.00004.

(D) Beta frequency band for Focus Baseline, Music and No Music sessions. The Friedman test showed a significant difference in PSD across electrodes during Baseline sessions, χ^2=13.5111, p-value=0.0090. There was a significant difference in PSD across electrodes during Music sessions, χ^2=15.8666, p-value=0.0032. There was a significant difference in PSD across electrodes during No Music sessions, χ^2=17.7777, p-value=0.0012.

(E) Gamma frequency band for Focus Baseline, Music and No Music sessions. The Friedman test showed a significant difference in PSD across electrodes during Baseline sessions, χ^2=11.6889, p-value=0.0198. There was a significant difference in PSD across electrodes during Music sessions, χ^2=9.6000, p-value=0.04773. There was a significant difference in PSD across electrodes during No Music sessions, χ^2=14.4034, p-value=0.0061.

(F) Delta frequency band for motivation Baseline, Music and No Music sessions. The Friedman test showed a significant difference in PSD across electrodes during Baseline sessions, χ^2=11.6000, p-value=0.0206. There was no significant difference in PSD across electrodes during Music sessions, χ^2=3.4400, p-value=0.4871. There was no significant difference in PSD across electrodes during No Music session, χ^2=7.4800, p-value=0.1130.

(G) Theta frequency band for motivation Baseline, Music and No Music sessions. The Friedman test showed a significant difference in PSD across electrodes during Baseline sessions, χ^2=14.6000, p-value=0.0056. There was a significant difference in PSD across electrodes during Music sessions, χ^2=11.6000, p-value=0.02059. There was a significant difference in PSD across electrodes during No Music sessions, χ^2=11.3200, p-value=0.0232.

(H) Alpha frequency band for motivation Baseline, Music and No Music sessions. The Friedman test showed a significant difference in PSD across electrodes during Baseline sessions, χ^2=18.1200, p-value=0.0012. There was a significant difference in PSD across electrodes during Music sessions, χ^2=19.0400, p-value=0.0008. There was a significant difference in PSD across electrodes during No Music sessions, χ^2=16.1600, p-value=0.0028.

(I) Beta frequency band for motivation Baseline, Music and No Music sessions. The Friedman test showed a significant difference in PSD across electrodes during Baseline sessions, χ^2=17.8000, p-value=0.0014. There was a significant difference in PSD across electrodes during Music sessions, χ^2=12.8000, p-value=0.0123. There was a significant difference in PSD across electrodes during No Music sessions, χ^2=18.9200, p-value=0.0008.

(J) Gamma frequency band for motivation Baseline, Music and No Music sessions. The Friedman test showed a significant difference in PSD across electrodes during Baseline sessions, χ^2=16.0400, p-value=0.030. There was a significant difference in PSD across electrodes during Music sessions, χ^2=8.5200, p-value=0.0742. There was a significant difference in PSD across electrodes during No Music sessions, χ^2=24.1200, p-value=0.00007.

Supplementary Figure 5

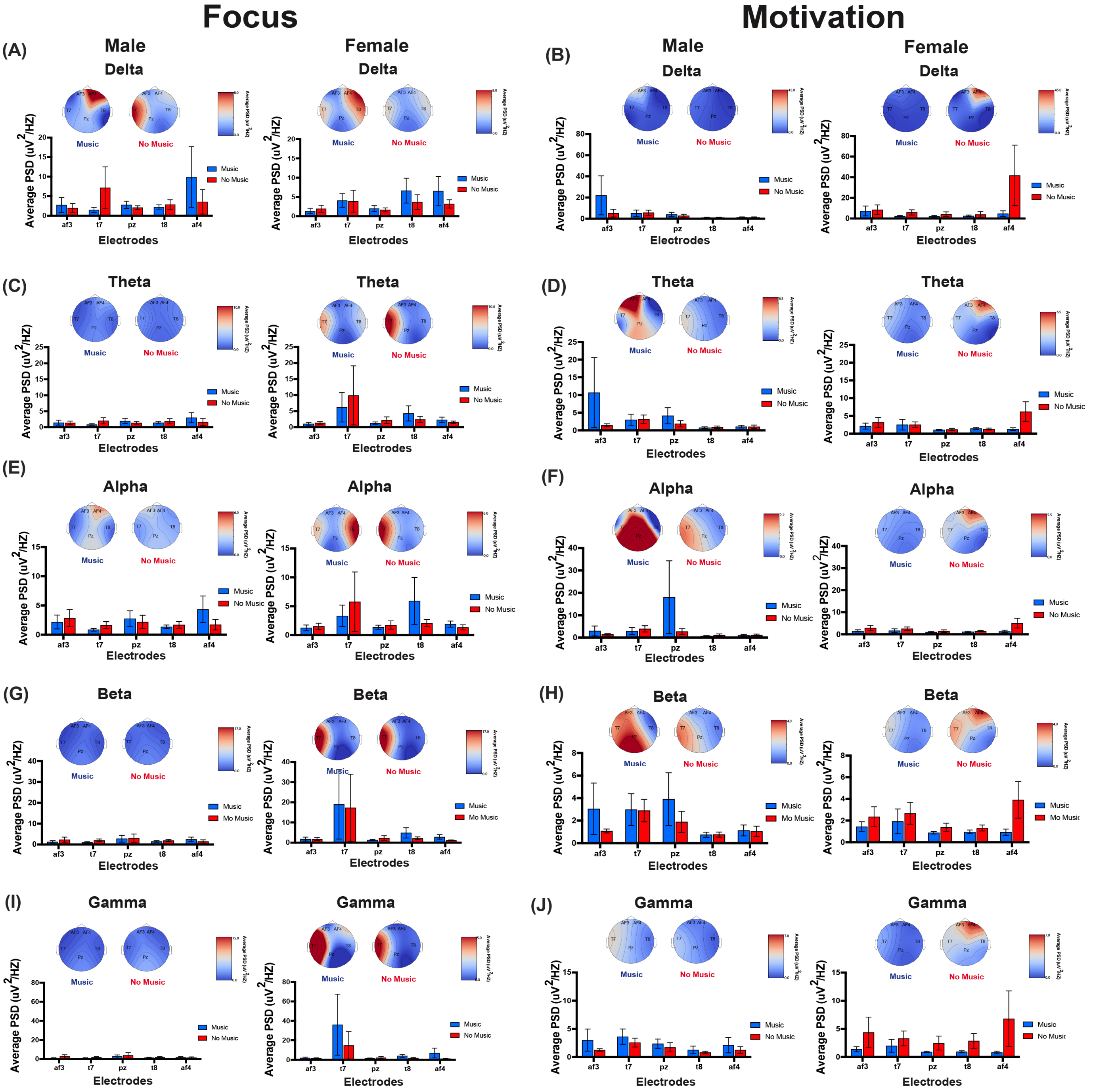

Supplementary Figure 5. Group Average EEG Power spectrum across frequency bands for males (n=7) and females (n=13) during music mindfulness sessions

(A) - (J) Group averaged power spectral density (PSD) and EEG topographic maps for Focus (Columns 1 and 2) and Motivation (Columns 3 and 4) conditions on normalized EEG data for male (left) and female (right) participants.

(A) ***Left*** Delta frequency band for Focus Music and No Music sessions for Male participants. The Friedman test did not show significant difference in PSD across electrodes during Music sessions, χ^2=3.0667, p-value=0.5467. There was no significant difference in PSD across electrodes during No Music sessions, χ^2=4.4000, p-value=0.3546.

Right Delta frequency band for Focus Music and No Music sessions for Female participants. The Friedman test did not show significant difference in PSD across electrodes during Music sessions, χ^2=4.8667, p-value=0.3013. There was no significant difference in PSD across electrodes during No Music sessions, χ^2=7.1333, p-value=0.1290.

(B) ***Left*** Delta frequency band for Motivation Music and No Music sessions for Male participants. The Friedman test did not show significant difference in PSD across electrodes during Music sessions, χ^2=2.5143, p-value=0.6421. There was no significant difference in PSD across electrodes during No Music sessions, χ^2=9.1429, p-value=0.0576.

Right Delta frequency band for Motivation Music and No Music sessions for Female participants. The Friedman test did not show significant difference in PSD across electrodes during Music sessions, χ^2=2.4000, p-value=0.6626. There was no significant difference in PSD across electrodes during No Music sessions, χ^2=1.4769, p-value=0.8307.

(C) ***Left*** Theta frequency band for Focus Music and No Music sessions for Male participants. The Friedman test did not show significant difference in PSD across electrodes during Music sessions, χ^2=6.0, p-value=0.1991. There was no significant difference in PSD across electrodes during No Music sessions, χ^2=1.7333, p-value=0.7846.

Right Theta frequency band for Focus Music and No Music sessions for Female participants. The Friedman test did not show significant difference in PSD across electrodes during Music sessions, χ^2=3.6000, p-value=0.4628. There was no significant difference in PSD across electrodes during No Music sessions, χ^2=7.5333, p-value=0.1102.

(D) ***Left*** Theta frequency band for Motivation Music and No Music sessions for Male participants. The Friedman test did not show significant difference in PSD across electrodes during Music sessions, χ^2=0.5714, p-value=0.9663. There was no significant difference in PSD across electrodes during No Music sessions, χ^2=8.2286, p-value=0.0836.

Right Theta frequency band for Motivation Music and No Music sessions for Female participants. The Friedman test did not show significant difference in PSD across electrodes during Music sessions, χ^2=2.2769, p-value=0.6850. There was no significant difference in PSD across electrodes during No Music sessions, χ^2=2.4615, p-value=0.6515.

(E) ***Left*** Alpha frequency band for Focus Music and No Music sessions for Male participants. The Friedman test did not show significant difference in PSD across electrodes during Music sessions, χ^2=5.4667, p-value=0.2427. There was no significant difference in PSD across electrodes during No Music sessions, χ^2=1.0667, p-value=0.8995.

Right Alpha frequency band for Focus Music and No Music sessions for Female participants. The Friedman test did not show significant difference in PSD across electrodes during Music sessions, χ^2=1.6667, p-value=0.7967. There was no significant difference in PSD across electrodes during No Music sessions, χ^2=3.0000, p-value=0.5578.

(F) ***Left*** Alpha frequency band for Motivation Music and No Music sessions for Male participants. The Friedman test did not show significant difference in PSD across electrodes during Music sessions, χ^2=3.3143, p-value=0.5067. There was no significant difference in PSD across electrodes during No Music sessions, χ^2=6.6286, p-value=0.1569.

Right Alpha frequency band for Motivation Music and No Music sessions for Female participants. The Friedman test did not show significant difference in PSD across electrodes during Music sessions, χ^2=1.6615, p-value=0.7977. There was no significant difference in PSD across electrodes during No Music sessions, χ^2=2.2154, p-value=0.6962.

(G) ***Left*** Beta frequency band for Focus Music and No Music sessions for Male participants. The Friedman test did not show significant difference in PSD across electrodes during Music sessions, χ^2=3.6000, p-value=0.4629. There was no significant difference in PSD across electrodes during No Music sessions, χ^2=1.4667, p-value=0.8325.

Right Beta frequency band for Focus Music and No Music sessions for Female participants. The Friedman test did not show significant difference in PSD across electrodes during Music sessions, χ^2=2.9333, p-value=0.5690. There was no significant difference in PSD across electrodes during No Music sessions, χ^2=3.9333, p-value=0.4151.

(H) ***Left*** Beta frequency band for Motivation Music and No Music sessions for Male participants. The Friedman test did not show significant difference in PSD across electrodes during Music sessions, χ^2=0.5714, p-value=0.9662. There was no significant difference in PSD across electrodes during No Music sessions, χ^2=8.8000, p-value=0.0663.
Right Beta frequency band for Motivation Music and No Music sessions for Female participants. The Friedman test did not show significant difference in PSD across electrodes during Music sessions, χ^2=1.9692, p-value=0.7414. There was no significant difference in PSD across electrodes during No Music sessions, χ^2=3.6308, p-value=0.4583.
(I) ***Left*** Gamma frequency band for Focus Music and No Music sessions for Male participants. The Friedman test did not show significant difference in PSD across electrodes during Music sessions, χ^2=2.9333, p-value=0.5690. There was no significant difference in PSD across electrodes during No Music sessions, χ^2=0.2667, p-value=0.9919.
Right Gamma frequency band for Focus Music and No Music sessions for Female participants. The Friedman test did not show significant difference in PSD across electrodes during Music sessions, χ^2=5.6000, p-value=0.2311. There was no significant difference in PSD across electrodes during No Music sessions, χ^2=4.7333, p-value=0.3158.
(J) ***Left*** Gamma frequency band for Motivation Music and No Music sessions for Male participants. The Friedman test did not show significant difference in PSD across electrodes during Music sessions, χ^2=1.3714, p-value=0.8491. There was no significant difference in PSD across electrodes during No Music sessions, χ^2=9.4857, p-value=0.05004.
Right Gamma frequency band for Motivation Music and No Music sessions for Female participants. The Friedman test did not show significant difference in PSD across electrodes during Music sessions, χ^2=1.6615, p-value=0.7977. There was no significant difference in PSD across electrodes during No Music sessions, χ^2=1.7846, p-value=0.7753.

Supplementary Figure 6

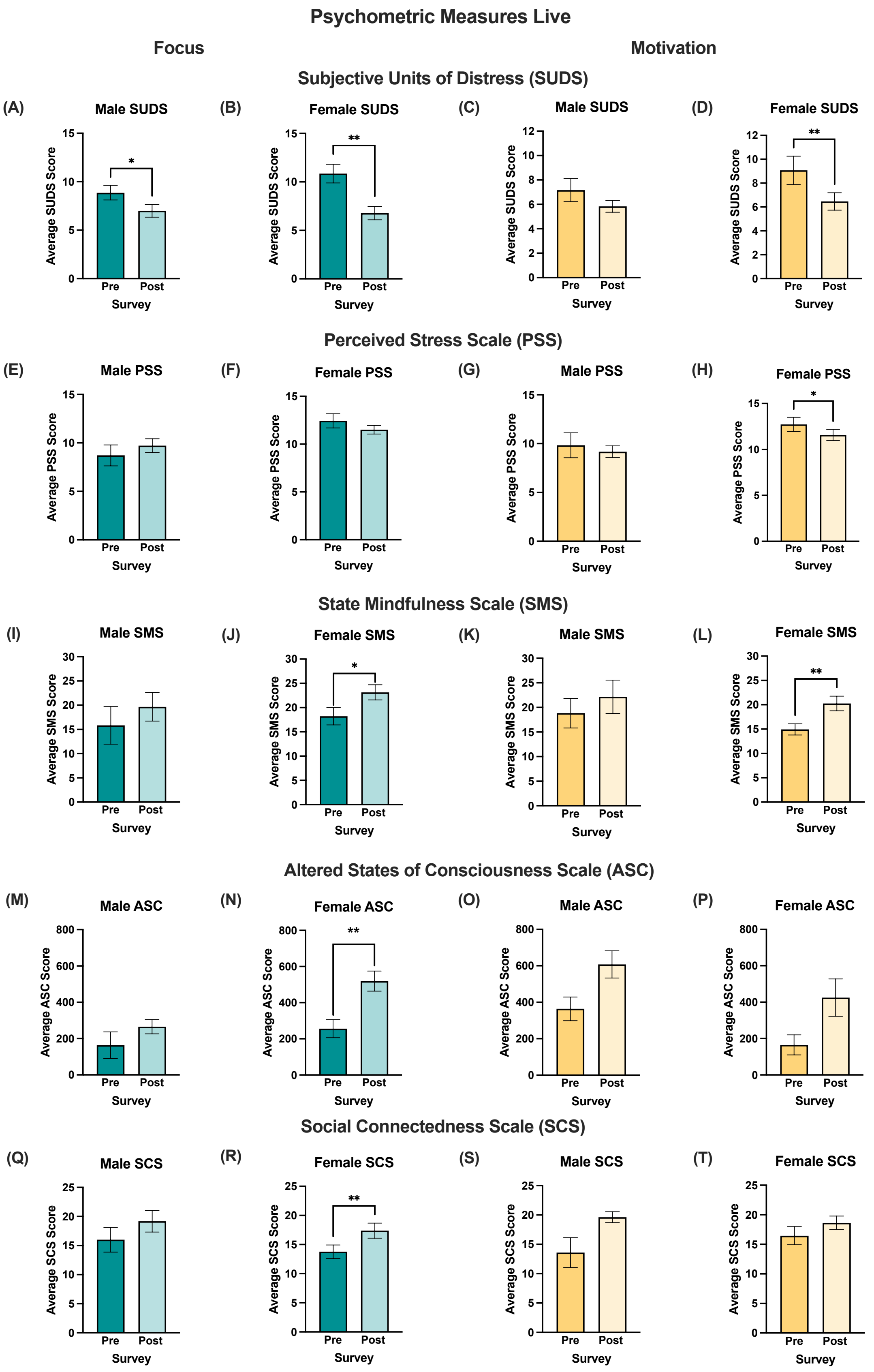

Supplementary Figure 6. **Comparing average pre survey scores and average post survey scores during the focus meditation and motivation meditation for all psychometrics across male and female participants.**

Male participants during focus meditation are on the first left column, female participants during focus meditation are on the second column, male participants during motivation meditation are on the third column, and female participants during motivation meditation are on the fourth column,

(A)SUDS post score was significantly lower than pre score during Focus meditation in males. Wilcoxon matched pairs signed rank test showed significance (W=-21, p=0.0312).

(B)SUDS post score was significantly lower than pre score during Focus meditation in females. Wilcoxon matched pairs signed rank test showed significance of (W=-93, p=0.0021).

(C)SUDS post score was not significantly lower than pre score during Motivation meditation in males. Wilcoxon matched pairs signed rank test showed no significance (W=-8, p=0.250).

(D)SUDS post score was significantly lower than pre score during Motivation meditation in females. Wilcoxon matched pairs signed rank test showed significance of (W=-55, p=0.0020).

(E)PSS post score was not significantly lower than pre score during Focus meditation in males. Paired t-test showed no significance (t=1.025, p=0.3450).

(F)PSS post score was not significantly lower than pre score during Focus meditation in females. Paired t-test showed showed no significance (t=1.605, p=0.1325)

(G)PSS post score was not significantly lower than pre score during Motivation meditation in males. Paired t-test showed no significance (t=0.8771, p=0.4206).

(H)PSS post score was significantly lower than pre score during Motivation meditation in females. Paired t-test showed showed significance (t=2.929, p=0.0117)

(I)SMS post score was not significantly higher than pre score during Focus meditation in males. Wilcoxon matched pairs signed rank test showed no significance (W=12, p=0.1875).

(J)SMS post score was significantly higher than pre score during Focus meditation in females. Wilcoxon matched pairs signed rank test showed significance of (W=77, p=0.0125).

(K)SMS post score was not significantly higher than pre score during Motivation meditation in males. Paired t-test showed no significance (t=1.730, p=0.1441).

(L)SMS post score was significantly higher than pre score during Motivation meditation in females. Paired t-test showed significance of (t=3.195, p=0.0065).

(M)ASC post score was not significantly higher than pre score during Focus meditation in males. Paired t-test showed no significance (t=0.3848, p=0.3848).

(N)ASC post score was significantly higher than pre score during Focus meditation in females. Paired t-test showed showed significance (t=4.149, p=0.0060)

(O)ASC post score was not significantly higher than pre score during Motivation meditation in males. Wilcoxon matched pairs signed rank test showed no significance (W=6, p=0.2500).

(P)ASC post score was not significantly higher than pre score during Motivation meditation in females. Wilcoxon matched pairs signed rank test showed showed no significance (W=10, p=0.1250)

(Q)SCS post score was not significantly higher than pre score during Focus meditation in males. Paired t-test showed no significance (t=2.188, p=0.0803).

(R)SCS post score was significantly higher than pre score during Focus meditation in females. Paired t-test showed showed significance (t=4.038, p=0.0016)

(S)SCS post score was not significantly higher than pre score during Motivation meditation in males. Paired t-test showed no significance (t=1.793, p=0.1475).

(T)SCS post score was not significantly higher than pre score during Motivation meditation in females. Paired t-test showed showed no significance (t=2.110, p=0.0611)

Supplementary Figure 7

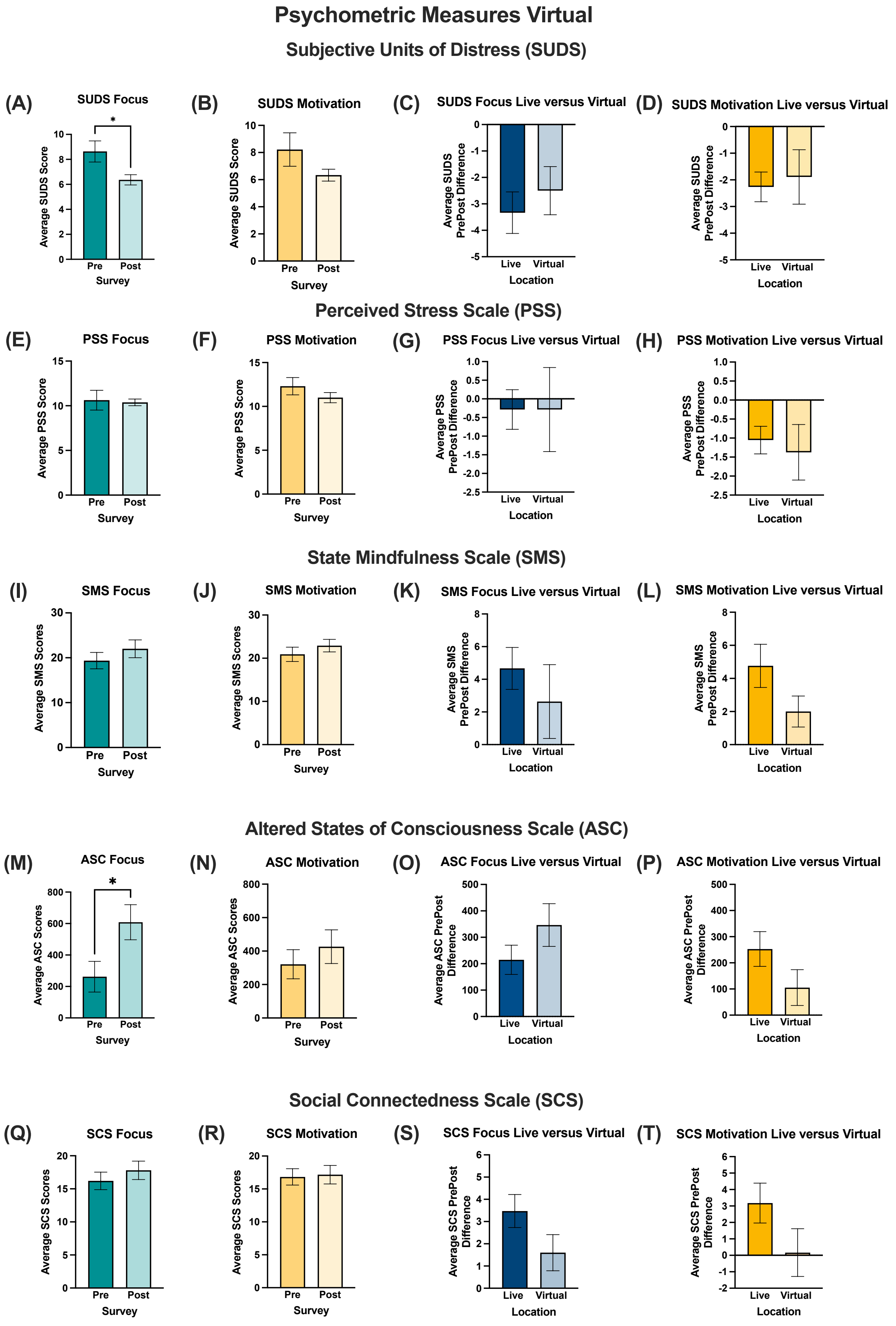

Supplementary Figure 7. Comparing average pre and post survey scores during the Focus and Motivation sessions for virtual and live participants

Third and Fourth columns compare the difference between post survey and pre survey scores (post survey minus pre survey score) sessions for all psychometrics across virtual and live participants.

(A)SUDS post score was significantly lower than pre score during Focus meditation. Paired t-test showed significance (t=3.173, *p=0.0238)

(B)SUDS post score was not significantly lower than pre score during Motivation meditation. Paired t-test showed no significance of (t=2.393, p=0.1012)

(C)SUDS PrePost difference Scores were not significant between Live (n=21) and Virtual (n=10) participants during focus meditation. Mann Whitney Test did not show significance (U=90.5, p=0.5491)

(D)SUDS PrePost difference Scores were not significant between Live (n=19) and Virtual (n=9) participants during motivation meditation. Mann Whitney Test did not show significance (U=71, p=0.4817).

(E)PSS post score was not significantly different than pre score during Focus meditation. Wilcoxon matched pairs signed rank test showed no significance (W=-2, p=0.9062).

(F)PSS post score was not significantly different than pre score during Motivation meditation. Wilcoxon matched pairs signed rank test showed no significance (W=-16, p=0.1562)

(G)PSS PrePost difference Scores were not significant between Live (n=21) and Virtual (n=7) participants during focus meditation. Unpaired t-test showed no significance (t=0.000, p>0.99)

(H)PSS PrePost difference Scores were not significant between Live (n=19) and Virtual (n=8) participants during motivation meditation. Unpaired t-test showed no significance (t=0.4421, p=0.6622).

(I)SMS post score was not significantly different than pre score during Focus meditation. Wilcoxon matched-pairs sign rank test did not show significance (W=33, p=0.1572).

(J)SMS post score was not significantly different than pre score during Motivation Meditation. Wilcoxon paired test showed significance (W=30, p=0.0859).

(K)SMS PrePost difference Scores were not significant between Live (n=21) and Virtual (n=11) participants during focus meditation. Mann Whitney test showed no significance (U=98.50, p=0.5111).

(L)SMS PrePost difference Scores were not significant between Live (n=21) and Virtual (n=11) participants during motivation meditation. Mann Whitney test showed no significance of (U=75.50, p=0.1146).

(M)ASC post score was significantly higher than pre score during Focus meditation. Paired t-test showed significance (t=4.291, *p=0.0233).

(N)ASC post score was not significantly different than pre score during Motivation meditation. Paired t-test showed no significance (t=1.537, p=0.1990).

(O)ASC PrePost difference Scores were not significant between Live (n=10) and Virtual (n=4) participants during focus meditation. Unpaired t-test showed no significance (t=1.298, p=0.2187).

(P)ASC PrePost difference Scores were not significant between Live (n=7) and Virtual (n=5) participants during motivation meditation. Unpaired t-test showed no significance (t=1.508, p=0.1625).

(Q)SCS post score was not significantly different than pre score during Focus meditation. Paired t-test showed no significance (t=1.969, p=0.1202).

(**R**)SCS post score was not significantly lower than pre score during Motivation meditation. Paired t-test showed no significance (t=0.2193, p=0.8351).

(S)SCS PrePost difference Scores were not significant between Live (n=19) and Virtual (n=5) participants during focus meditation. Unpaired t-test showed no significance (t=1.230, p=0.2315).

(T)SCS PrePost difference Scores were not significant between Live (n=17) and Virtual (n=6) participants during motivation meditation. Unpaired t-test showed showed no significance (t=1.347, p=0.1925)

Focused Attention and Motivation Instructions and Script

Instructions to tell participants before: Thank you for coming! Before we begin, we ask if you could please remain seated and minimize interactions for two minutes. More specifically, we ask if your knees are facing at a 90 degree angle, both feet flat on the floor, hands on thighs, eyes closed and palms facing upward. The sessions will be bilingual in English and Spanish. You will be guided in what to do when the meditation is not in your native language. The session begins when you hear a sound or a voice. You will be prompted when the session is over. If you feel uncomfortable at any point, you can take off the device. We recommend if you can to try to keep it on as long as possible, but we completely understand if you have the need to take it off.

¡Gracias por venir! Antes de comenzar, si pudieran permanecer sentados y minimizar las interacciones durante dos minutos. Si pudieran sentarse de manera que sus rodillas formen un ángulo de 90 grados, ambos pies planos en el suelo, manos sobre los muslos, ojos cerrados y palmas hacia arriba. Las sesiones serán bilingües en inglés y español. Se les guiará sobre qué hacer cuando la meditación no esté en su idioma nativo. La sesión comienza cuando escuchan un sonido o una voz. Se les indicará cuando la sesión haya terminado.

-

Focus 15-minute bilingual instructions (English/Spanish)

This meditation will be a bilingual meditation in English and Spanish. As I transition from one language to the next, continue to focus on the instructions given in your language. There will be moments where we pause during the meditation to give you time to follow the guided instructions.

Esta meditación será bilingüe, en inglés y español. Cuando cambie de un idioma a otro trate de enfocarse en las instrucciones de su idioma. Habrán momentos de pausa durante la meditación para darle tiempo de seguir las instrucciones.

10,9,8,7,6…..

Good afternoon, welcome to this meditation session on focus. Buenas tardes, bienvenidos a esta sesión de meditación sobre el enfoque

Whether it’s your first time practicing or you’ve practiced a few times, see if you can begin by acknowledging that there is no right or wrong way to practice.

Si es la primera vez que está practicando o tal vez ya haya practicado varias veces, intente de comenzar con reconocer que no existe una manera correcta o incorrecta de practicar.

If distractions come up at any time, gently allow yourself to return to the guidance of my voice, your breath or the body part of focus.

Si se distrae en cualquier momento, solamente vuelva a la guía de mi voz, su respiración, o la parte del cuerpo en que nos estemos enfocando.

Begin to settle into the posture that was recommended.

Comience a adoptar la postura que fue recomendada.

You can keep the eyes closed or maybe just soften the eyelids and focus the gaze down a few inches in front of you.

Puede mantener los ojos cerrados o quizás bajar y enfocar la mirada hacia el piso en frente de usted.

Once you feel settled, begin to bring your awareness to your body as a whole. Gently scan the body from the crown of the head, shoulders, side body, legs and down to the feet…then back up from the feet, to the legs, the side body, shoulders, and back to the crown of the head. Scan your body from the crown of the head to the feet a few times on your own.

Cuando se sienta relajado en esa postura, comience a poner su atención en el cuerpo entero. Comience a escanear lentamente su cuerpo desde la cabeza, hacia los hombros, los lados del cuerpo, las piernas y los pies… y desde los pies, hacia las piernas, los lados del cuerpo, los hombros, y a la cabeza de nuevo. Escaneando el cuerpo varías veces.

Notice if your body is feeling relaxed, tense, energized, or tired. Being in observation of what the feeling in your body is without judging it. Note si su cuerpo se siente relajado, tenso, con energía, o cansado. Observando la sensación en el cuerpo, sin juzgar la sensación.

Then begin to bring your awareness and focus to the state of your mind. Noticing the headspace…are there thoughts moving in and out, fogginess, alertness, confusion, or clarity. Again, just being with a curiosity about what's present and trying to let go of the judgment that may show up as you continue to become aware.

Ahora, comience a llamar su atención y enfoque al estado de su mente. Quizás hay pensamientos presentes o la mente se siente nublada, alerta, hay confusión o claridad. Tratar de estar en curiosidad de lo que está presente sin juzgar.

Now begin to shift the focus of your attention to the sensation of your breath. Beginning to notice the natural pattern of your breath.

Comience a enfocar su atención a la sensación de su respiración. Notando el patrón natural de su respiración.
Notice the sensation of the nostrils as you inhale and exhale, maybe sensing for the coolness of the breath as it enters the nostrils and a bit of warmth of the breath as it exits the nostrils.

Noté la sensación en la nariz durante la inhalación y exhalación, tal vez sintiendo la temperatura de la inhalación un poco fresca y la temperatura de la exhalación un poco más tibia.

Now begin to follow the breath even more deeply into your body. Focus on the sensation of the breath as it begins its path entering the nostrils, filling the lungs and the expansion of the belly. Keeping your awareness on the inhale.

Ahora comience a seguir la respiración aún más profundamente en el cuerpo. Enfóquese en la respiración entrando por la nariz, llenando los pulmones, y la expansión del estómago. Poniendo atención en la sensación de la inhalación solamente.
Now focus on the sensations of the exhale as the belly softens,lungs empty and the breath exiting through the nostrils.
Ahora comience a enfocarse en la sensación de la exhalación. Notando la forma en que el estómago se relaja, los pulmones se vacían, y la respiración sale por la nariz.

Begin to focus on the way the breath enters and leaves the body. Notice each inhale and exhale.

Comencé a enfocarse en la forma en que la respiración entra y sale del cuerpo. Notando cada inhalación y exhalación.

If the mind wanders, without judging the thoughts or distractions, see if you can bring your focus back to the awareness of your breath entering and leaving the body. Knowing that you can redirect your focus back to the breath as many times as you need to.

Si la mente se distrae en cualquier momento, sin juzgar los pensamientos, o distracciones, trate de volver a enfocarse en la forma en que la respiración entra y sale del cuerpo. Sabiendo que puede dirigir el enfoque nuevamente a la respiración todas las veces que sea necesario.

Begin to move the awareness of the breath just to the belly.

As you breathe in, notice how the belly expands and lifts, and as you breathe out notice how the belly softens and releases. See if you can just keep the focus of your awareness right here, the rise and the fall of the belly as you breathe in and as you breathe out.

Comience a mover su atención hacia el estómago. Mientras inhala, notando como el estómago se expande y se eleva. Y mientras exhala note como el estómago se relaja y baja. Trate de enfocarse aquí por unos momentos, con el movimiento del estómago expandiéndose y relajándose con cada respiración.

And once more bringing your awareness back up to your nostrils. Being with the sensation of the breath entering and exiting the nose…noticing the coolness…the warmth for a few rounds.

Y una vez más comience a mover la atención hacia la nariz. Por unos momentos notando la sensación de la respiración entrando y saliendo de la nariz…Notando la frescura y calidez de cada respiración.

Gently begin to release the awareness of the breath and expand the awareness back to your body. From the nose to the face, head, down to shoulders, side body, the legs, all the way down to the feet. And from the feet, back up to the legs, the side body, and back up to the head. Do this a few times at your own pace.

Lentamente comience a soltar la atención de la respiración. Y comenzar a expandir y seguir la atención hacia su cuerpo. De la nariz hacia la cara, la cabeza, los hombros, los lados del cuerpo, las piernas, y hacia los pies. Y de los pies, de nuevo hacia las piernas,los lados del cuerpo, los hombros y hacia la cabeza.

Begin to release the scan of the body and maybe invite deeper breaths in and out.

Dejando de escanear el cuerpo, y tal vez comenzar a invitar una respiración más profunda al cuerpo. When you feel ready, begin to invite subtle movements back into your body. Small movements into the fingers and toes.

Y cuando se sienta listo, comience a invitar pequeños movimientos al cuerpo. Pequeños movimientos a los dedos de las manos y los pies.

If the eyes were closed, beginning to open them, or lifting the gaze.

Take your time.

Si los ojos están cerrados comience a abrirlos o a levantar la mirada. Tome su tiempo.

10,9,8,7,6…

Motivation 15-minute bilingual instructions (English/Spanish)

This meditation will be a bilingual meditation in English and spanish. As I transition from one language to the next, continue to focus on the instructions given in your language. There will be moments where we pause during the meditation to give you time to follow the guided instructions.

Esta meditación será bilingüe, en inglés y español. Cuando cambie de un idioma a otro trate de enfocarse en las instrucciones de su idioma. Habrán momentos de pausa durante la meditación para darle tiempo de seguir las instrucciones.
10,9,8,7……

Good evening, welcome to this meditation on motivation. Whether it's your first time practicing or you've practiced a few times,we begin by acknowledging that there is no right or wrong way to practice and my voice is here just as a guide.
Buenas tardes, bienvenidos a esta meditación enfocada en la motivación. Si es la primera vez que práctica o ya ha practicado varias veces, comenzamos reconociendo que no existe una forma correcta o incorrecta de practicar y que mi voz es solamente un guía.

Begin to settle into the posture that was recommended.

Comience a adoptar la postura que fue recomendada.

You can keep the eyes closed or maybe just soften the eyelids and focus the gaze down a few inches in front of you.

Puede mantener los ojos cerrados o quizás bajar y enfocar la mirada hacia el piso en frente de usted.

Begin to notice your natural breathing pattern, noticing how the breath enters and exits the body. Cultivating a little more relaxation with each passing breath but still fully alert.
Comience a notar su patrón natural de respiración, notando como la respiración entra y sale del cuerpo. Con cada respiración que pase, cultivando un poco más de relajación en su cuerpo pero manteniéndose alerta.

During the meditation if you notice thoughts or distractions come up, you can return to noticing your natural breathing pattern or the tone of my voice as many times as you need to.

Durante la meditación, si la mente se distraer puede regresar a notar su patrón de respiración o al tono de mi voz las veces que sean necesarias.

Now , begin thinking about what motivates you in your daily life and how this inspiration moves you towards your desires. This may feel hard to do or unnatural but take a moment to just notice what comes up for you without judgment as you breathe in and out.

Ahora, comience a pensar en las cosas que lo motivan en su vida diaria y como esa inspiración le ayuda a acercarse a sus deseos. Hacer esto se puede sentir difícil o innatural, pero trate de estar presente con estos pensamientos sin juzgar mientras inhala y exhala.

Sometimes we get stuck, feeling unmotivated due to many different factors, whether it's a need to slow down or there are feelings getting in your way of engaging with daily tasks.
This meditation can help you focus on what matters to help keep you moving forward.

A veces nos sentimos desmotivados. Puede ser por una necesidad de tomar una pausa o pueden haber sentimientos que se interpongan en la forma en que participe en las tareas diarias. Esta meditación le puede ayudar a enfocarse en las cosas que son importantes para ayudarle a seguir adelante.

Motivation helps us stay focused, present and actively involved in our daily lives.

La motivación nos ayuda a enfocarnos, a sentirnos presentes y a participar activamente en nuestras vidas diarias.

In the moments where motivation does not seem accessible, know that there is so much value and courage in taking a step back to connect to the balance and creativity in the process.

En los momentos que la motivación no se siente accesible, sepa que hay mucho valor y valentía en tomar un paso hacia atrás para reconectar con el balance y creatividad del proceso.

Sometimes thoughts are overwhelming while planning and trying to visualize the final outcome.

Right here in this moment, see if you can take a pause to come back to noticing your breath and the sensations that this may bring up in your body, without judgment… noticing each passing breath entering and exiting the body.

A veces los pensamientos pueden ser abrumadores, especialmente mientras está tratando de planear o visualizar el resultado final.En este momento, tome una pausa para reconectar con su respiración, notando la sensaciones que pueden estar presente aquí en su cuerpo sin juzgar…notando la respiración entrando y saliendo de su cuerpo.

When you nourish these moments of slowing down, connection and commitment, you foster creativity, and it helps complete tasks with intention.

Momentos como estos de descanso, conexión y compromiso pueden brindar creatividad y ayudar a completar tareas con intención.

Motivation can be cultivating mindful awareness of your values, intentions, and aspirations.
La motivación puede ser cultivando una conciencia hacia sus valores, intenciones, y aspiraciones.

Motivation can also be rest, compassion, love and honesty. La motivación también puede ser descanso, compasión, amor y honestidad.

Take another pause to connect to yourself and to notice the sensations or emotions that may be coming up for you at this moment. Using your breath as a tool to help keep you present.

Tome otra pausa para conectar con si mismo y notar las sensaciones o emociones que están presente para usted en este momento. Usando su respiración como una herramienta para ayudarlo a mantenerse presente.

If at this moment you are not connecting with any particular thought i invite you to maybe connect with love and compassion through the process of motivation.

Si en este momento no se está conectado con ningún pensamiento, lo invito a conectarse con compasión y amor en el proceso de la motivación.

Try to keep connection to this feeling as you begin to reconnect to your breath. Holding this feeling of love and compassion as you breathe in and breathe out.

Trate de mantener esta conexión con este sentimiento mientras comienza a reconectarse con su respiración.

Sosteniendo este sentimiento de amor y compasión mientras inhala y exhala.

Notice the connection that moments of rest like these can be the bridge between where you are and where you want to be in life.

Note lo importante que son los momentos de descanso como estos en conexión con donde está y donde quiere estar en su vida.

Begin to deepen your breath and bring awareness to your body being in this space.

Comience a respirar más profundo y a traer su conciencia a este espacio de nuevo.

When you feel ready, begin to invite subtle movements back into your body. Small movements into the fingers and toes.

Y cuando se sienta listo, comience a invitar pequeños movimientos al cuerpo. Pequeños movimientos a los dedos de las manos y los pies.

If the eyes were closed, beginning to open them, or lifting the gaze. Take your time.

Si los ojos están cerrados comience a abrirlos o a levantar la mirada. Tome su tiempo.
